# Design and characterization of novel pyridylbenzimidazole-based ruthenium(II) complexes, and their evaluation in singlet oxygen generation

**DOI:** 10.3762/bjoc.22.126

**Published:** 2026-11-04

**Authors:** Benjamin Riss Yaw, Anna Porcher, Alice Saunier, Jean-Christophe Cintrat, Eugénie Romero

**Affiliations:** 1 Département Médicaments et Technologies pour la Santé (DMTS) SCBM, Université Paris Saclay, CEA, INRAE Gif-sur-Yvette 91191, France

**Keywords:** energy transfer, photoredox catalysis, ruthenium polypyridyl complexes, single electron transfer

## Abstract

Six new ruthenium(II) polypyridyl complexes incorporating pyridinyl, quinolinyl, or isoquinolinyl-benzimidazole ligands were synthesized to investigate the influence of π-conjugated mono- and bis-chelating ligands on their photophysical and electrochemical properties, and compared to the frontrunner [Ru(bpy)_3_](PF_6_)_2_. Their evaluation reveals that ligand π-extension modulates the ILCT and MLCT transitions, leading to red-shifted absorption and tunable excited-state redox potentials. The mononuclear complexes were further evaluated as visible-light photocatalysts through reactive oxygen species (ROS) generation and a model α-arylation reaction. These findings demonstrate that benzimidazole-based ligand engineering provides an effective strategy to tune the excited-state properties and photocatalytic behavior of ruthenium complexes.

## Introduction

Among photoactivated metal complexes, ruthenium polypyridyl complexes represent a major and versatile family of coordination compounds [1–3]. These complexes are appreciated for their rich photophysical and electrochemical properties [4–6]. One of the many reasons for their high interest lies in their photophysical behavior [7–8]. Ruthenium polypyridyl complexes exhibit absorption bands in the UV–visible region with high molar extinction coefficients, mainly attributed to metal-to-ligand charge transfer (MLCT) transitions. The corresponding excited states possess relatively long lifetimes and emit high phosphorescence at room temperature. Importantly, these properties can be modulated by ligand modification, allowing fine control over absorption and emission wavelength, lifetime, and quantum yield [1–3]. In addition to their high chemical stability and accessible redox potentials (oxidative/reductive quenching and energy transfer), these features make them robust systems in many domains [4,9–12].

Ruthenium polypyridyl complexes are investigated in biomedicine as photosensitizers in photodynamic therapy (PDT), where the excitation of ruthenium complexes allows the production of reactive oxygen species, leading to cytotoxic effects [13]. Their easy modular structure via ligand modification further allows the incorporation of targeting carriers or functional groups, improving their solubility, expanding their potential as therapeutic and diagnostic tools [14]. Beyond biology, these complexes play a key role in photoredox catalysis and in materials science with solar energy conversion, due to their favorable redox behavior and photostability [10,15–18].

**Figure 1:**
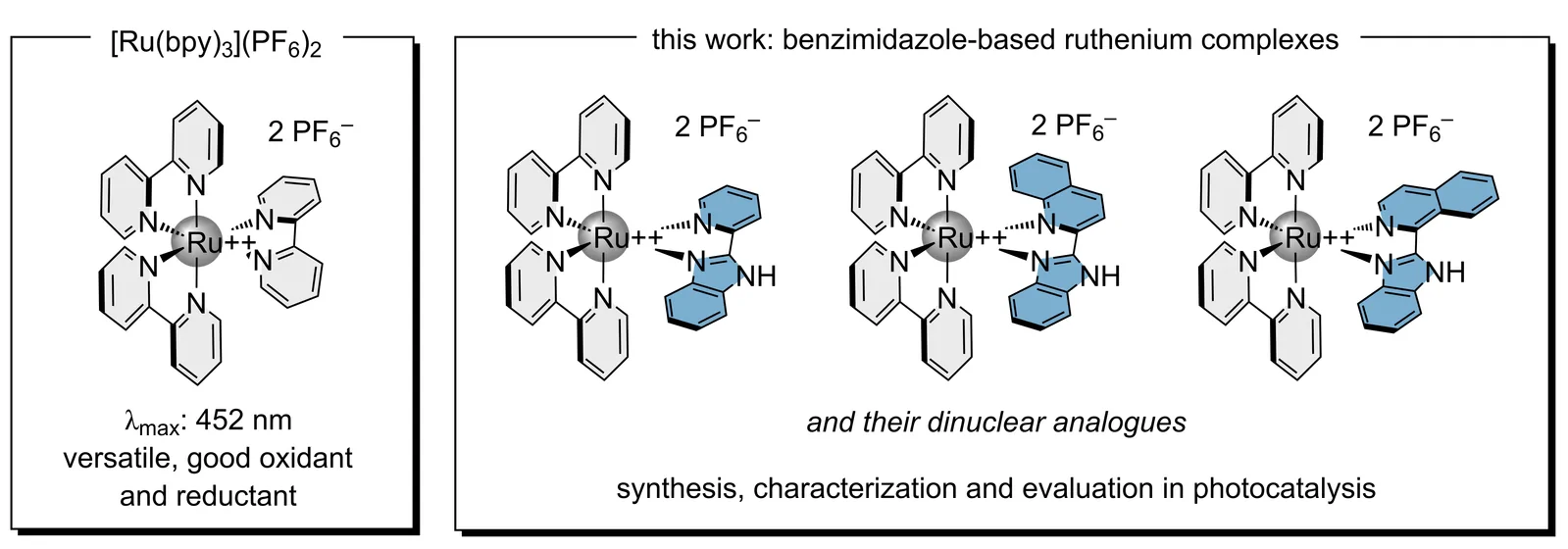
Structures of [Ru(bpy)_3_](PF_6_)_2_ as a widely used versatile photocatalyst and the complexes evaluated in this study.

Among ruthenium polypyridyl complexes, [Ru(bpy)_3_](PF_6_)_2_ is the frontrunner and exhibits a strong MLCT absorption at ≈450 nm and emits at ≈620 nm with an excited-state lifetime of ≈600 ns. In its excited state, [Ru(bpy)_3_]^2+^* exhibits an estimated oxidation potential of approximately −1.31 V vs Fc/Fc^+^ and a reduction potential around +0.41 V vs Fc/Fc^+^, enabling it to act as both a good photo-reductant and photo-oxidant (Figure 1) [19–20].

Due to their modular ligand structure, particular attention has been paid to systems with π-conjugated, electron-accepting ligands containing a benzimidazole unit, such as pyridinyl-benzimidazole [21–22], quinolinyl-benzimidazole [13,23–24], pyridyl-benzobisimidazole [25–26], pyridyl-quinonebisimidazole [27–28] pyrenyl-bisimidazole [29], and related derivatives [30–31]. However, the optical and electronic properties of systems based on quinolinyl/pyridinyl-imidazole and symmetric bisimidazole ligands remain largely unexplored (Figure 1).

The first studies on complexes coordinated with pyridyl-benzimidazole ligands, described by Haga and Tanaka in 1979 [32], showed that these ligands, L, had an excellent ability to chelate and form [Ru(bpy)_2_L]^2+^ complexes. Furthermore, their studies demonstrated that the donor nature of these ligands had a strong impact on the redox potential, as well as on the metal-ligand charge transfer (MLCT) bands responsible for the optical properties of Ru(II) polypyridyl complexes [22,32–33].

In recent years, several studies have explored complexes containing pyridyl-benzazole as a third ligand, including pyridyl-benzimidazole ligands, which have demonstrated their usefulness in various applications such as catalysis [24,34–36], cancer phototherapy [37–46] and photochemistry [44,47–49]. However, only two examples exist in the literature exploring [Ru(bpy)_2_L]^2+^ complexes where L corresponds to pyridyl-benzimidazole and quinolinyl-benzymidazole in such applications [24,44].

Following on from mono-chelating ligands, bis-chelating ligands such as 2,6-bis(benzimidazol-2-yl)pyridine and its derivatives have been widely used for the synthesis of bis-nuclear ruthenium(II) complexes with tunable photophysical and electrochemical properties [27–28,50–54]. These complexes often exhibit greater intensity and red shift of the MLCT absorption bands as well as the corresponding emission band [54–58]. Other studies have also shown that these benzobisimidazole ligands exhibit interesting electronic properties that can be adjusted by modifying the ligand's π system, in particular by introducing substituents that extend electronic conjugation and modify the energy levels of orbitals involved in MLCT transitions [59], such as quinoline or isoquinoline. Although polypyridyl systems have been extensively studied, ligands based on a benzodiimidazole structure chelated with two ruthenium(II) metals remain relatively unexplored in the literature [50–54,60–61]. Furthermore, the electronic and optical properties of quinolinyl and isoquinolinyl analogues have not yet been studied to date.

Imidazole derivatives incorporated into heavy metal complexes (such as Ir(III)) have been studied for their luminescent properties, particularly in OLED devices [62–64]. Varying the substituents on the imidazole or combining it with π-conjugated ligands can modulate the emission color, excited state lifetime, and quantum efficiency. However, very few studies specifically cover symmetrical bisimidazole ligands or quinolinyl/pyridinyl-imidazole combinations with lighter transition metals such as ruthenium, leaving a gap in our understanding of the fundamental properties of these structures. This represents a significant opportunity for investigation to understand the influence of electronic transitions (π–π*, n–π*, CT) and the potential applications of such complexes in photocatalysis.

Indeed, quinolinyl/pyridinyl-imidazole and symmetric bisimidazole ligands are of interest in the design of new metal complexes due to the combination of a strong N-centered chelating ability with extended π-conjugation and tunable electronic properties [54,65–66]. On one hand, the imidazole/benzimidazole core provides a solid coordination site that can stabilize the metal oxidation states, and on the other hand the quinolinyl or pyridinyl substituents act as electron-donating or electron-withdrawing units, enabling the modification of the redox potential and frontier orbital energies. The π-conjugated framework is likely to improve light absorption and facilitate charge transfer, or MLCT (metal-to-ligand charge transfer) lifetimes. In the case of symmetric bisimidazoles, the possibility of chelating two ruthenium metal centers offers the possibility of cooperative or synergistic properties. Indeed, when bridging two metals, such ligands can promote electronic communication between the two metal centers, leading to effects on redox processes [67], as they can display broadened or shifted absorption profiles, or modification of redox potentials compared to their mononuclear analogues [68]. The ability to tune the distance between the two metal sites has also been utilized to mimic metalloenzyme active sites, design catalysts with multi-electron reactivity, or create artificial photosynthetic systems capable of charge separation and long-lived excited states [69]. Due to these structural features, quinolinyl/pyridinyl-imidazole and bisimidazole ligands are valuable ligands for the development of new ruthenium complexes with fine-tuned functionalities for diverse applications such as photocatalysis. It is indeed of interest to examine how such π-conjugated quinolinyl and pyridinyl-imidazole or bisimidazole ligands, when coordinated to one or two ruthenium centers, influence the electronic structure and photoluminescent behavior of the resulting complexes [70].

**Scheme 1:**
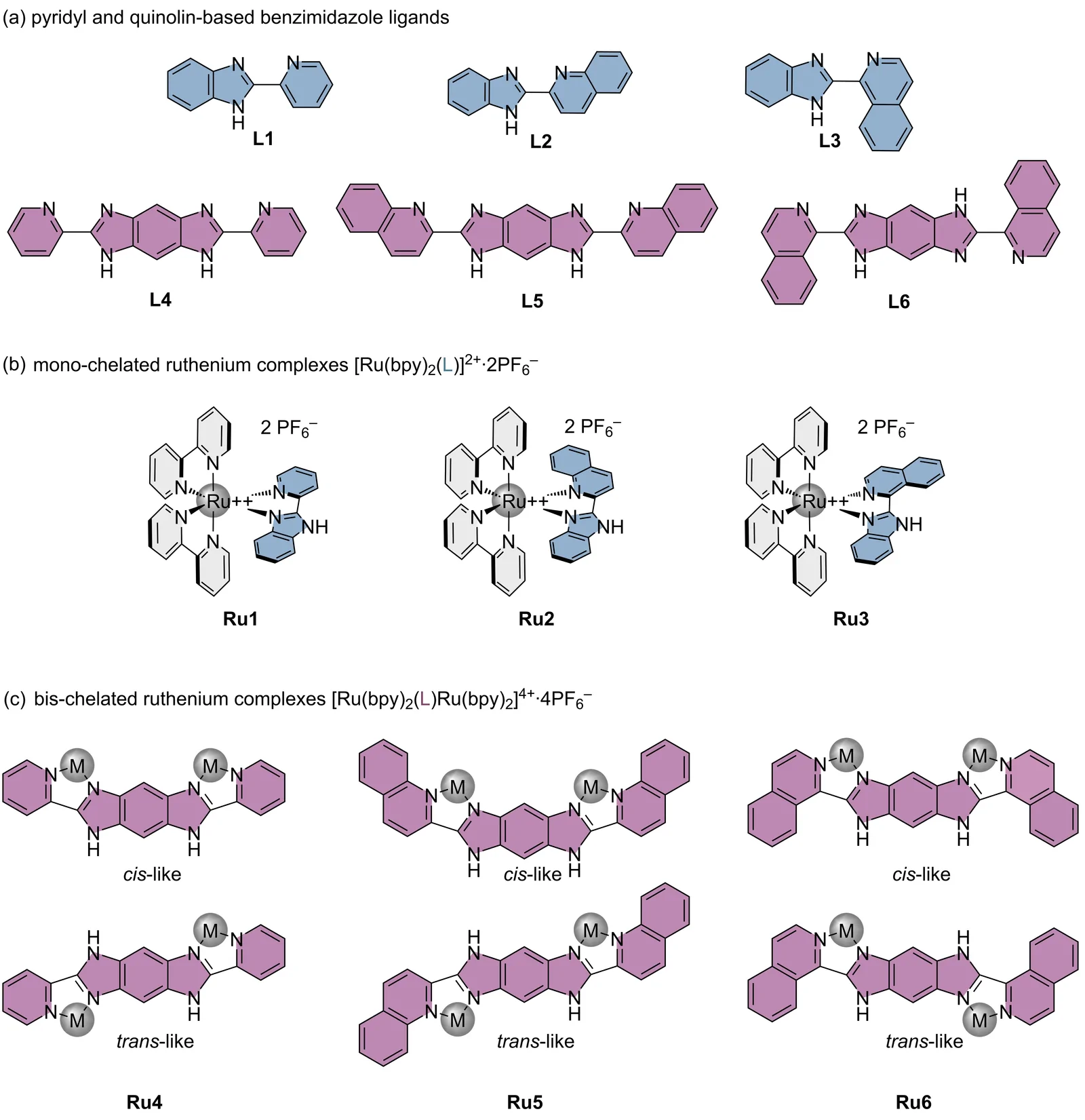
Schematic representation of (a) **L1**–**6**, (b) **Ru1**, **Ru2**, **Ru3** mono-ruthenium, and (c) **Ru4**, **Ru5**, and **Ru6** bis-ruthenium complexes. M = [Ru(bpy)_2_]^2+^·2PF_6_^−^.

In this work, we report on the design and synthesis of six new ruthenium complexes obtained using 2-(pyridin-2-yl)-1*H*-benzo[*d*]imidazole, 2-(1*H*-benzo[*d*]imidazol-2-yl)quinoline, and 1-(1*H*-benzo[*d*]imidazol-2-yl)isoquinoline ligands. Depending on their architecture, these ligands enable the formation of either mononuclear or dinuclear (bis-chelated) ruthenium complexes **Ru1**–**3** and **Ru4**–**6**, respectively, providing a platform to systematically investigate the influence of ligand structure on the optical and electronic properties of the resulting assemblies (Scheme 1).

A series of analyses, i.e, cyclic voltammetry, UV–visible absorption spectroscopy, and photoluminescence measurements, was applied to evaluate their optical and electronic behavior. Beyond fundamental insights, these new complexes will also be explored as photosensitizers for photocatalytic transformations, via energy transfer and electron-transfer processes.

## Results and Discussion

To evaluate the potential of new pyridyl-benzimidazole based ruthenium(II) complexes, we have focused our efforts on 2-(pyridin-2-yl)-1*H*-benzo[*d*]imidazole (**L1**), 2-(1*H*-benzo[*d*]imidazol-2-yl)quinoline (**L2**), and 1-(1*H*-benzo[*d*]imidazol-2-yl)isoquinoline (**L3**) for the design of new mono-chelated ruthenium complexes, to evaluate the influence of such ligands in the optical and electronical properties of the complexes [71–72].

**Scheme 2:**
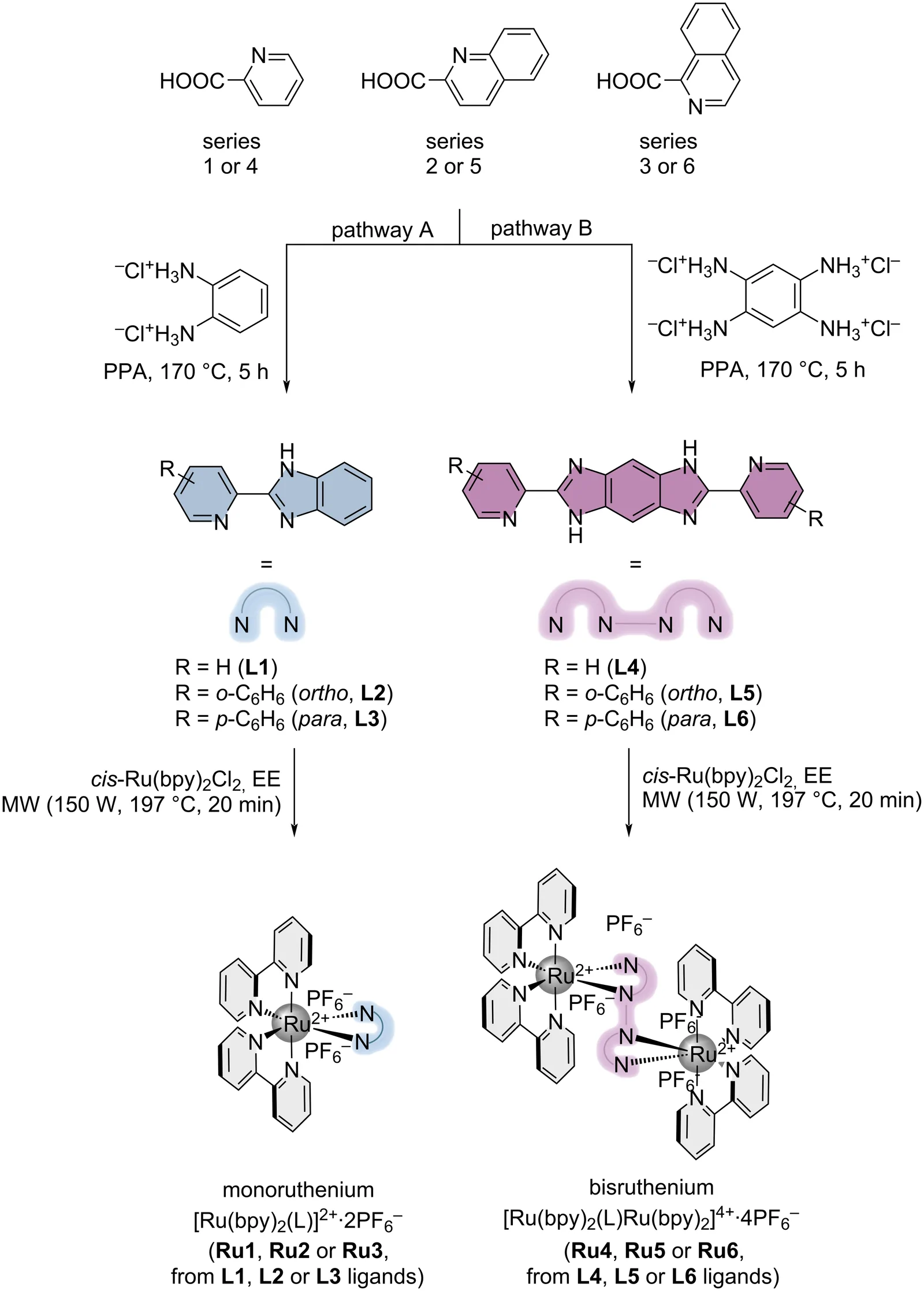
Synthesis of monoruthenium (**Ru1**, **Ru2**, **Ru3**) and bisruthenium (**Ru4**, **Ru5**, **Ru6**) complexes from the pyridinyl/quinolinyl-benzimidazole (**L1**, **L2**, **L3**) or bisimidazole ligands (**L4**, **L5**, **L6**). EE = 2-ethoxyethanol.

The synthesis of ligands was performed, followed by a cyclizing condensation reaction, under strongly dehydrating conditions, between a diamine and an aromatic carboxylic acid derivative, providing access to two distinct families of benzo-imidazole ligands via two reaction pathways (pathways A and B, Scheme 2). The starting amino compounds, picolinic acid (for **L1** and **L4**), quinaldic acid (for **L2** and **L5**), and isoquinoline-1-carboxylic acid (for **L3** and **L6**) corresponding to the desired ligands were subjected to cyclization conditions in the presence of polyphosphoric acid (PPA) at 170 °C for 5 hours to promote both the activation and condensation of the carboxylic acid on the amine to form the corresponding nitrogen-containing cyclic products. To obtain the benzimidazole ligands **L1** to **L3**, the reaction was carried out between the corresponding carboxylic acid and 1,2-benzenediamine hydrochloride according to reaction pathway A (Scheme 2).

The ligands thus obtained were isolated with yields of 24%, 83%, and 73%, respectively. In the case of the benzobisimidazole ligands, **L4** to **L6**, they were obtained under the same conditions as above, according to reaction pathway B. Thus, condensation was carried out between the corresponding carboxylic acid and 1,2,4,5-tetraaminobenzene tetrahydrochloride to obtain the isolated benzobisimidazole ligands with quantitative yields for **L4** and **L5** versus 40% for **L6**. The increase in structural rigidity and steric hindrance caused by the *para* substituent could explain this low yield.

The synthesis of mono (**Ru1**–**3**) and bis-chelated (**Ru4**–**6**) complexes was performed by a coordination reaction of ligands and 1 or 2 equivalents of the dichlororuthenium dibipyridine complex following pathway A for **L1**–**3**, and pathway B for **L4**–**6**. All reactions were performed in 2-ethoxyethanol (EE) using a microwave process. Subsequently, the complexes are directly isolated following a salt metathesis reaction involving the exchange of the chloride salt by the PF_6_ counterion (Scheme 2).

With these complexes in hand, we have evaluated their optical properties and performed the absorption and emission spectra of the whole series.

**Figure 2:**
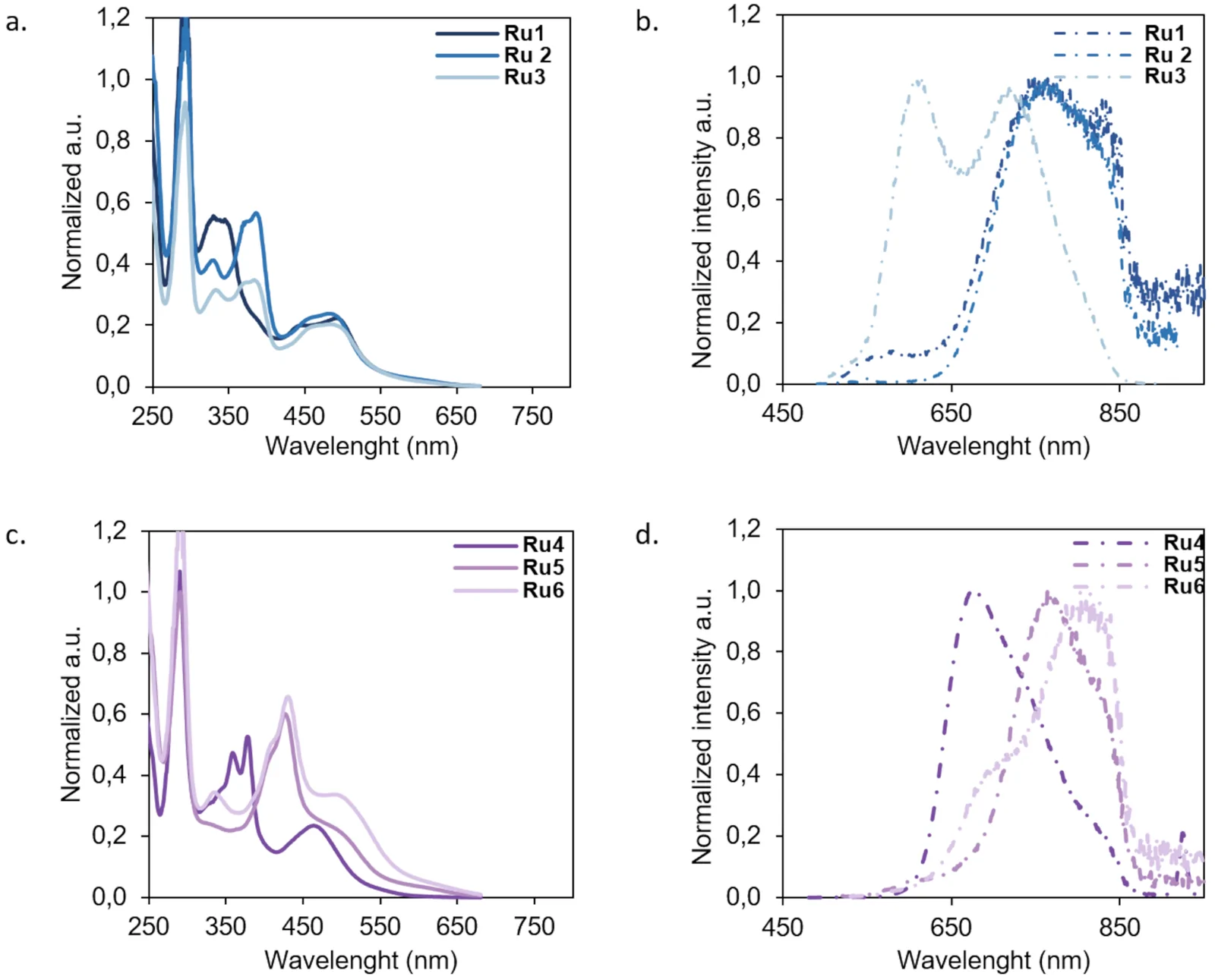
Normalized (a) absorbance and (b) emission spectra excited at λ_max_ (MLCT Ru → L*) at 100 µM in CH_3_CN at room temperature of **Ru1** (drak blue line), **Ru2** (blue line), **Ru3** (light blue line); (c) absorbance and (d) emission spectra excited at λ_max_ (MLCT Ru → L*) at 100 µM in CH_3_CN at room temperature of **Ru4** (dark purple line), **Ru5** (purple line), and **Ru6** (light purple line) complexes.

**Optical properties:** The absorption and emission spectra of complexes **Ru1**–**6**, together with the associated photophysical data, recorded at 100 μM in CH₃CN at 25 °C, are presented in Figure 2 and Table 1. All six complexes display absorption features characteristic for ruthenium polypyridyl systems. In general, the spectra exhibit an intense band between 270 and 300 nm, attributed to the π→π* intraligand transition of the bipyridine moiety, corresponding to an interligand charge transfer (ILCT) transition. A second broad absorption band is observed between 385 and 650 nm, with distinct maxima at 462, 483, 492, 504, and 508 nm, assigned to MLCT transitions from the Ru d orbitals to the ligand π* orbitals (Figure 2a–d). Compared to the well-studied [Ru(bpy)_3_]^2+^ complex, the MLCT band of **Ru1**–**6** is broader, a difference that can be explained by the presence of two overlapping excited states, Ru→bpy* and Ru→L*. In addition, a third absorption band, characteristic of ILCT transitions within the quinolinyl- and pyridinyl-imidazole ligands, is detected between 300 and 430 nm for the mononuclear complexes **Ru1**–**3** and the dinuclear complex **Ru4**, and between 375 and 450 nm for the dinuclear complexes **Ru5** and **Ru6** (Figure 2a–d).

**Table 1:** Photopysical data for **Ru1–6**.^a^

complexes	λ MLCT (nm) (ε in 10^−3^ M^−1^·cm^−1^)	λ emission^b^ (nm)	Δ Stokes (nm)	Φ^c^ (%)	*E*_00_ (eV)

**Ru1**	446 (11.48), 491 (12.60)^d^	749	121, 260^d^	0.95	2.36
**Ru2**	457 (12.84), 483 (13.64)^d^	762	279^d^	0.55	2.00
**Ru3**	462 (11.30), 484 (11.68)^d^	719	145, 245^d^	0.22	2.32
**Ru4**	462 (13.80)^d^	676	214^d^	0.57	2.14
**Ru5**	504 (8.30)^d^	764	258^d^	0.38	2.11
**Ru6**	508 (18.10)^d^	803	273, 294^d^	0.17	2.02

^a^All measurements were performed at 50 μM for **Ru1–6** in CH_3_CN at 293 K; ^b^values with λ excitation = 450 nm (absorption wavelength; ^c^Φ quantum yield was calculated according to the equation, using [Ru(bpy)_3_]^2+^ (Φ = 1.8%) as the standard [73]; ^d^values corresponding at λ_max_ of MLCT.

For the mono-chelated complexes **Ru1**–**3**, the ILCT absorption band undergoes a progressive red shift from 340 nm in **Ru1** to 388 and 390 nm in **Ru2** and **Ru3**, respectively. A slight bathochromic shift is also observed in the MLCT Ru→bpy* band, with maxima at 446 nm (**Ru1**), 457 nm (**Ru2**), and 462 nm (**Ru3**) (Figure 2a and Figure 2b). This effect is attributed to differences in the aromatic extension of the π-conjugated systems in the quinolinyl- versus pyridinyl-imidazole ligands. Moreover, the withdrawal of electron density from the third ligand away from the metal center contributes to a decrease in the Ru→bpy* MLCT transition energy. In parallel, a small hypsochromic shift is observed for the Ru→L* MLCT band, with maxima at 491 nm (**Ru1**), 483 nm (**Ru2**), and 484 nm (**Ru3**), further supporting the role of ligand electronic density in modulating MLCT transitions (Figure 2a, Figure 2b, and Table 1).

In the dinuclear complexes **Ru4**–**6**, the bathochromic shift of the ILCT band is more pronounced. The ILCT maximum shifts from 340 nm in **Ru1** to 379 nm in **Ru4** (Δ = 39 nm), and up to 432 nm in **Ru6** (Δ = 92 nm compared to **Ru1**, and Δ = 42 nm compared to its mononuclear analogue **Ru3**) (Table 1). This trend reflects the extension of the π-conjugated framework in the bisimidazole ligands, which induces a significant red shift of the ILCT transition. In contrast, the red shift of the MLCT bands in the dinuclear complexes relative to their mononuclear counterparts is modest. For instance, the MLCT maximum at 483 nm in **Ru2** shifts to 504 nm in **Ru5** (Δ = 21 nm), while the 484 nm band in **Ru3** shifts to 508 nm in **Ru6** (Δ = 24 nm). Thus, coordination of a quinolinyl-imidazole ligand to two [Ru(bpy)_2_]^2+^ units does not induce a major red shift of the Ru→L* MLCT transition (Table 1). Furthermore, the bis-coordination of quinolinyl-bisimidazole in **Ru5** and **Ru6** appears to result in overlap between the ILCT band of the bisimidazole ligand and the Ru→bpy* MLCT band, likely due to the energetic proximity of these two transitions, which merge into a broad envelope spanning 375–470 nm.

Interestingly, in **Ru4** the Ru→L* MLCT band undergoes a significant hypsochromic shift from 492 nm (**Ru1**) to 462 nm, accompanied by band sharpening. This feature suggests closer energetic alignment of the Ru→bpy* and Ru→L* MLCT states. The resulting increase in the Ru→L* transition energy points to an electronic environment around the ruthenium centers in **Ru4** that is highly similar to that provided by the bipyridine ligands. Overall, these results indicate that the number of coordinated ruthenium units is not the primary determinant of MLCT red shifts in these systems. Instead, the effect arises from a combination of the close spatial proximity of the two ruthenium centers and the localized electron density of the bis-coordinated ligands, which generate an electronic environment comparable to that of the bipyridine ligands, as exemplified by **Ru4**.

Fluorescence spectra of the mononuclear (**Ru1**–**3**) and dinuclear (**Ru4**–**6**) complexes were recorded at different excitation wavelengths (Figure 2), revealing diverse emission profiles. When excited at the maximum absorption wavelength of the Ru→L* MLCT transition, the complexes exhibit two principal emission bands: one of moderate intensity in the 550–650 nm range (**Ru1**, **Ru3**, and **Ru5**) and a broader, more intense band between 650 and 900 nm (**Ru1**–**6**).

In particular, **Ru1** and **Ru3** display sharp emission maxima at 580 and 613 nm, respectively. These narrow bands are attributed to radiative relaxation from the triplet ^3^MLCT Ru→bpy* state to the ground state (S_0_). In addition, all complexes show a second, broader emission band of higher intensity extending from 630 to 900 nm, with maxima at 676 nm (**Ru4**), 762 and 764 nm (**Ru2** and **Ru5**), and 803 nm (**Ru6**) (Table 1). This feature is assigned to nonradiative relaxation from the ^3^MLCT Ru→L* excited state.

Overall, the varied emission profiles of **Ru1**–**6** reveal the existence of closely spaced triplet states capable of mediating relaxation between multiple pathways. Both mononuclear and dinuclear complexes thus exhibit extended excited-state lifetimes, consistent with efficient electronic communication between the ruthenium centers and the π-conjugated quinolinyl- or pyridinyl-imidazole/bisimidazole ligands, ultimately leading to phosphorescent emission.

**Figure 3:**
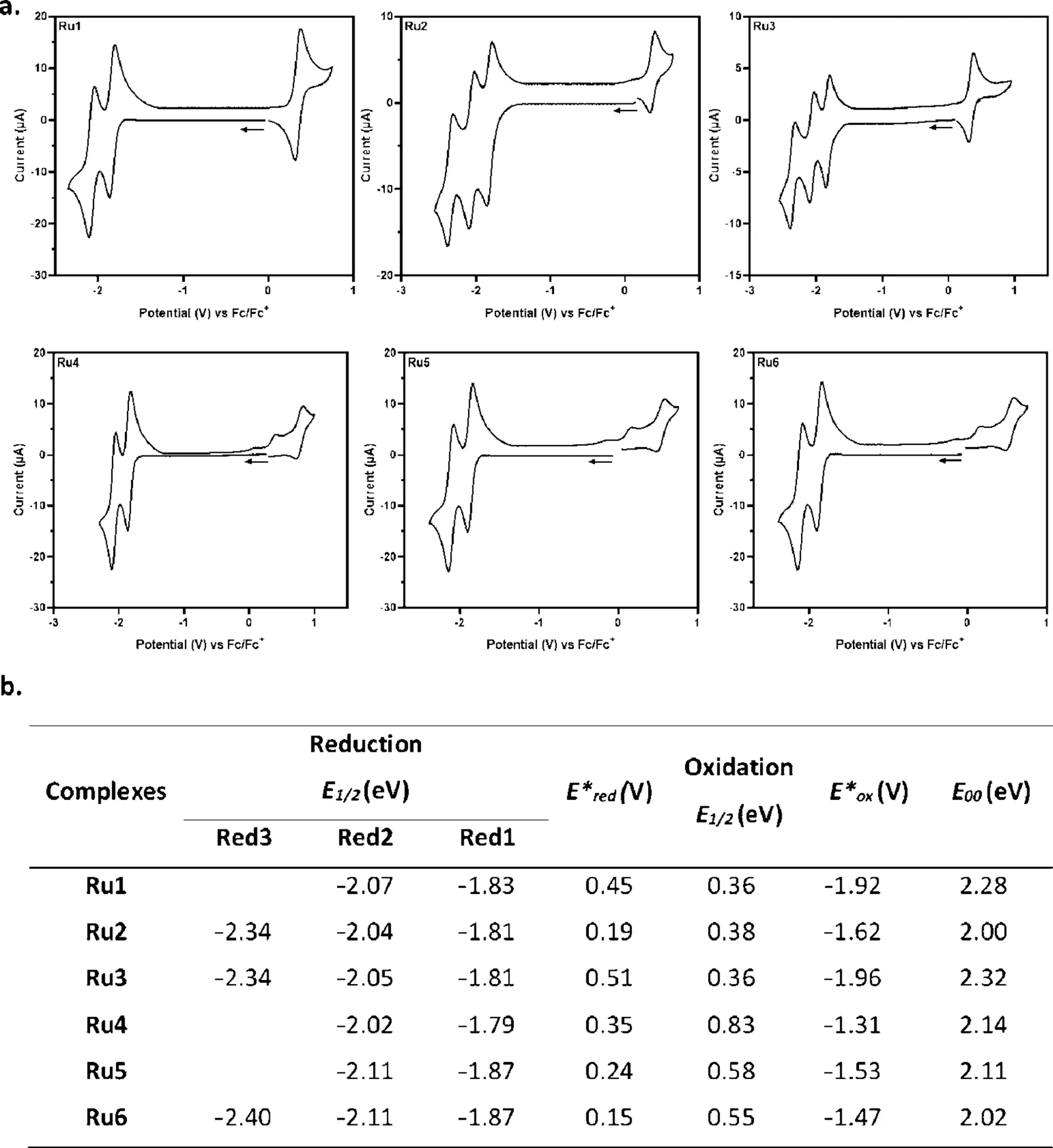
All measurements were performed in 0.1 M *n*-Bu_4_NPF_6_/MeCN. The potential is reported as *E*_1/2_ versus Fc/Fc^+^. A) Graph of the cyclic voltammetry of **Ru1***–***6**; b) all measurements summarized in a table. The excited state redox potential (E*) of complexes was calculated based on the Rehm*–*Weller theory [74–75].

**Electronic properties:** The electronic properties of complexes **Ru1**–**6** were evaluated by cyclic voltammetry analysis and compared to the frontrunner [Ru(bpy)_3_](PF_6_)_2_. The observed trend can be rationalized by considering the electronic properties of the substituted benzimidazole ligands and their influence on the MLCT excited state. The excited-state redox potential is related to the ground-state metal oxidation or reduction potentials and the zero–zero excitation energy *E*_00_ according to the Rehm–Weller relationship (Figure 3). The excited-state reduction potentials, E*_red_, and oxidation potentials, E*_ox_, of the three heteroleptic Ru(II) complexes **Ru1–3**, as well as the dinuclear complexes **Ru4–6**, were determined from the corresponding ground-state redox potentials and the zero–zero excitation energy, *E*_00_. For comparison, the benchmark complex [Ru(bpy)_3_](PF_6_)_2_ displays an excited-state reduction potential of approximately +0.41 V vs Fc/Fc^+^.

As such, both the metal-centered oxidation/reduction potentials and the energy of the MLCT transition determine the overall driving force for the excited state. Among these factors, variations in the ligand π* energy, which dominates *E*_00_ are typically most influential for polypyridyl-type complexes. The investigated complexes exhibit E*_red_ values ranging from +0.15 to +0.51 V. Among the mononuclear complexes, **Ru3** displays the most positive E*_red_ value (+0.51 V), followed by **Ru1** (+0.45 V), whereas **Ru2** exhibits the lowest value (+0.19 V). Thus, **Ru3** is the strongest excited-state oxidant within the series, although **Ru1** and **Ru3** complexes are stronger oxidants than the benchmark [Ru(bpy)_3_](PF_6_)_2_. The excited-state oxidation potentials, E*_ox_, show a complementary trend, ranging from −1.31 to −1.96 V. Among the mononuclear complexes, **Ru3** exhibits the most negative value (−1.96 V), followed by **Ru1** (−1.92 V) and **Ru2** (−1.62 V). Consequently, **Ru3** also possesses the strongest excited-state reducing power of the mononuclear series, whereas **Ru2** is the weakest. These results demonstrate that modification of the ancillary ligand framework provides substantial control over both the oxidative and reductive properties of the excited Ru(II) complexes. Indeed, in **Ru1**, the coordinating ligand contains a relatively small aromatic fragment (pyridine–benzimidazole), providing less extended π-conjugation and a higher-lying π* orbital compared with the quinoline- and isoquinoline-based analogues. This results in a higher-energy MLCT transition and a larger *E*_00_ (2.28 eV). Consequently, **Ru1** displays a relatively positive E*_red_ (+0.45 V) and a strongly negative E*_ox_ (−1.92 V), indicating substantial excited-state oxidizing and reducing capabilities. In contrast, **Ru2** incorporates a quinoline moiety, whose extended π-system stabilizes the ligand-centered π* orbital. This leads to a lower MLCT energy and a smaller ***E_00_*** (2.00 eV), resulting in a less positive E*_red_ (+0.19 V) and a less negative E*_ox_ (−1.62 V). Accordingly, **Ru2** is both the weakest excited-state oxidant and the weakest excited-state reductant among the three mononuclear complexes. The isoquinoline ligand in **Ru3** provides a π-system comparable in size to that of quinoline but with a different orbital arrangement and conjugation pathway through the benzimidazole fragment. **Ru3** exhibits the largest *E*_00_ of the mononuclear series (2.32 eV), together with the most positive E*_red_ (+0.51 V) and the most negative E*_ox_ (−1.96 V). Thus, **Ru3** combines the strongest excited-state oxidizing and reducing powers within the mononuclear series.

For the bis-chelated complexes **Ru4–Ru6**, both E*_red_ and E*_ox_ are shifted toward less extreme values compared with their mononuclear analogues. In particular, E*_red_ decreases from +0.45/+0.19/+0.51 V for **Ru1**–**3** to +0.35/+0.24/+0.15 V for **Ru4**–**6**, while E*_ox_ becomes substantially less negative, ranging from −1.31 to −1.53 V. This indicates that the dinuclear complexes possess both reduced excited-state oxidizing and reducing abilities relative to the mononuclear analogues. The observed changes can be rationalized, at least in part, by the lower *E*_00_ values associated with the red-shifted absorption and emission of the dinuclear complexes. Because *E*_00_ contributes positively to E*_red_ but negatively to E*_ox_, a decrease of *E*_00_ necessarily shifts both excited-state potentials toward less extreme values, thereby reducing both the excited-state oxidizing and reducing power.

With all these elements in hand, the ligand-dependent modulation of the MLCT energy appears to be an important factor governing the excited-state redox properties. The potential of **Ru1**–**3** will be evaluated and confirmed in photocatalyzed mediated transformations.

### Generation of type I and type II ROS

**Figure 4:**
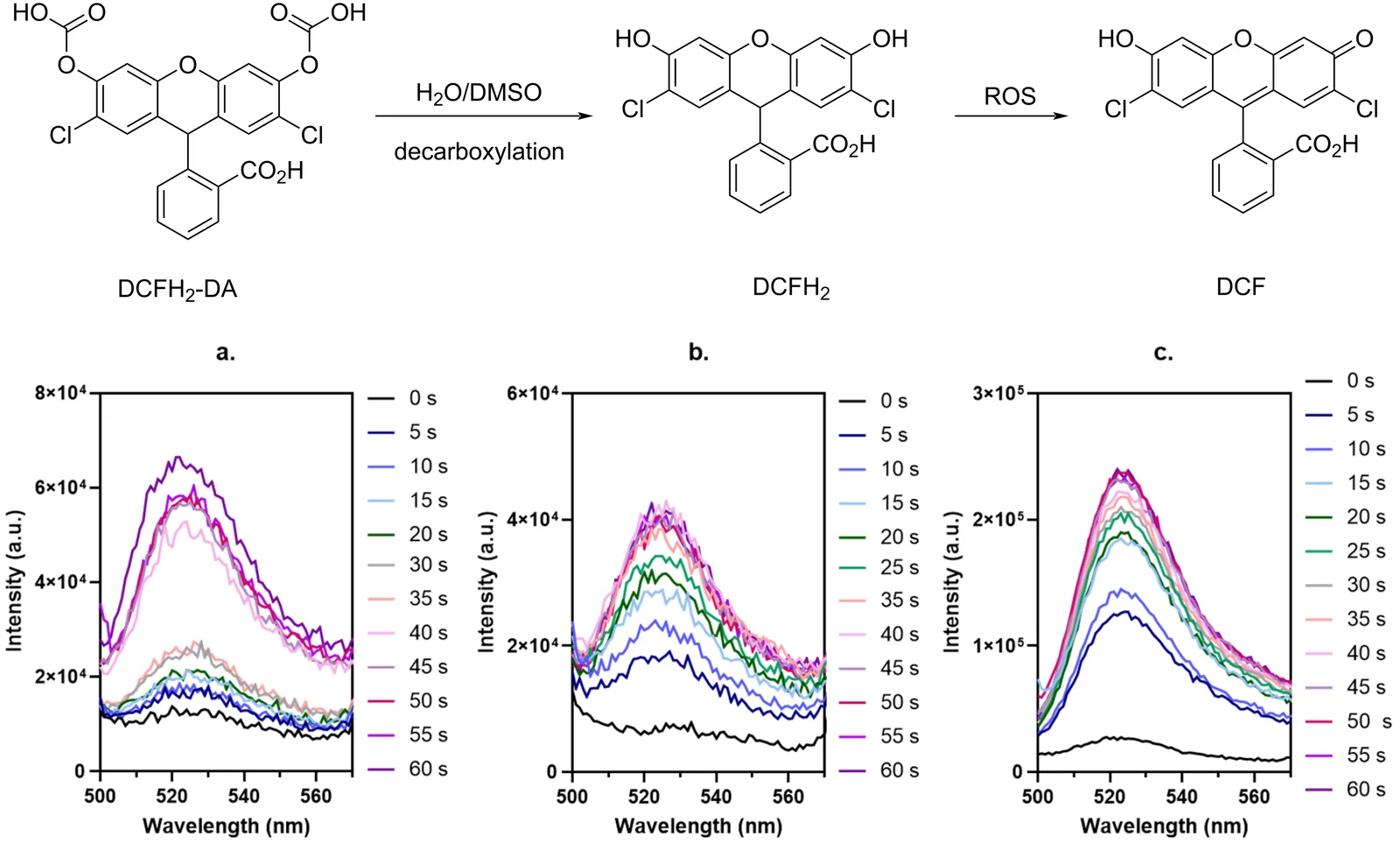
Emission spectra at 550 nm obtained by the measurement of the fluorescence intensity resulting from the oxidation of DCFH to DCF corresponding to the ROS generation by (a) **Ru1**, (b) **Ru2**, and (c) **Ru3**. Each measurement was monitored under irradiation at 456 nm (40 W) of a solution containing 5 µM of DCFH and 5 µM of [Ru] in a mixture of H_2_O/DMSO (199:1, v/v).

**Type I:** The ROS generation capacity of type I was studied under irradiation at 457 nm for each complex in the presence of a photosensitizer, 2,7-dichlorodihydrofluorescein (DCFH). DCFH was selected for its ability to oxidize to green fluorescent DCF species, which significantly increases green fluorescence in this form. Each photo-transformation of DCFH to DCF was carried out in an H₂O/DMSO (99.5:0.5) solution containing 5 µM of DCFH and the complex under study (Figure 4) [76].

As shown in the graph, the evolution of the signal correlated with ROS generation by electron transfer was monitored at 5-second intervals between each measurement for 60 seconds under irradiation of 457 nm at 40 W of power. Comparison of each complex with the control demonstrates the generation, to varying degrees, of ROS by a process of electron transfer (SET). The DCFH signal appears to decrease slightly to an *I*/*I*_0_ ratio of 3 after 60 seconds of irradiation. This is due to the natural auto-oxidation of DCFH to DCF. [Ru(bpy)_3_](PF_6_)_2_ shows linear and continuous growth up to a ratio of 6.7, which reflects a gradual and constant production of ROS. The **Ru1** study shows a moderate increase at the beginning, followed by a more marked increase after 35 seconds of irradiation, reaching a ratio of approximately between 4.5 to 4.7 after 60 seconds of irradiation. Furthermore, ROS production remains lower than [Ru(bpy)_3_](PF_6_)_2_. In contrast, analysis of **Ru2** and **Ru3** shows a kinetically faster and more intense production of ROS from the very first seconds compared to [Ru(bpy)_3_](PF_6_)_2_. This indicates a more favorable electron transfer from these structures compared to [Ru(bpy)_3_](PF_6_)_2_. Nevertheless, the ROS progression of **Ru2** seems to stabilize at a ratio of around 6 to 6.3. **Ru3** appears to be the most effective at generating ROS through a SET mechanism. Indeed, the signal suddenly increases to a ratio of 7 after 15 seconds of irradiation, reaching a plateau between 8.5 and 9. Consequently, **Ru3** appears to be the most promising photocatalyst in the series for ROS production via SET, with the highest kinetics and plateau in the series. This indicates a strong ability to transfer these electrons to a photosensitizer, as well as to produce ROS. These observations demonstrate a significant influence of the structure of the third ligand on the SET process for ROS generation. Furthermore, this seems to suggest a close correlation with the electron density around the ruthenium nucleus. A highly localized electron density around the nucleus would be unfavorable for a SET process. Thus, the **Ru3** complex would be the most promising candidate for the emergence of applications involving a SET reaction process or strong ROS generation of type I.

**Figure 5:**
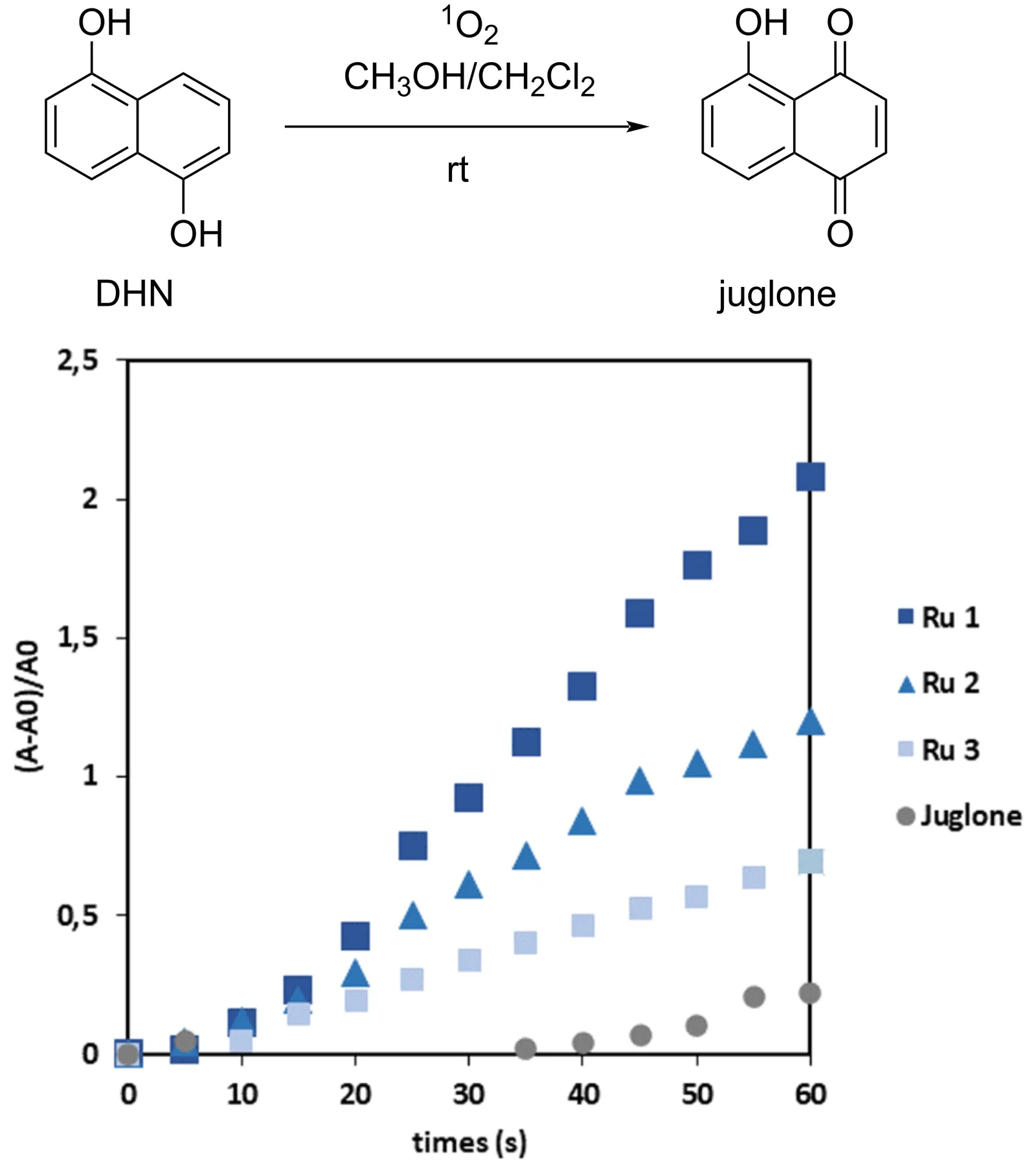
Graph showing the total singlet O_2_ generation measured for each complex: **Ru1**, **Ru2**, and **Ru3**. Each measurement was monitored in a solution containing 310 µM of DHN and 31 µM of Ru in a mixture of MeOH/DCM (1:9, v/v), previously bubbled with O_2_, under irradiation at 456 nm.

**Type II:** The ability of **Ru1**, **Ru2**, and **Ru3** to generate type II ROS through energy transfer (EnT) was also studied for each of these complexes in the presence of 1,5-dihydronaphthalene (DHN) under irradiation at 456 nm. DHN is a probe that absorbs between 260 and 350 nm and is sensitive to the presence of singlet oxygen, which produces an oxidized species, juglone, that absorbs at 420 nm (Figure 5) [77].

Each photooxidation of DHN was carried out in a MeOH/DCM (1:9) solution, previously saturated by oxygen bubbling for 10 min, containing 310 µM of DHN and 31 µM of the complex under study. As shown in the graph (Figure 5), the generation of singlet oxygen ^1^O_2_ correlates with the growth of the absorption band corresponding to the appearance of juglone at 420 nm after a 5-second interval of irradiation by analysis for 60 seconds, according to the following Equation 1:


[1] c[O12]=A(420 nm)−A0(420 nm)A0(420 nm)

*A* = the absorbance intensity of the complex at 420 nm and *A*0 = the initial absorbance intensity of the complex at 420 nm.

Comparison of the curves corresponding to the experiments with and without complexes highlights the generation of singlet oxygen for all the complexes studied. Furthermore, the fact that in the experiment without complexes, growth begins after 35 to 40 seconds from 0.05 to reach 0.2 in juglone after 60 seconds of irradiation indicates that the complex plays an essential role in the generation of ^1^O_2_ following the photo-oxidation of DHN. In the case of [Ru(bpy)_3_](PF_6_)_2_, the curve appears to show low ^1^O_2_ production during the irradiation time (0.3 to 60 seconds). This indicates moderate efficiency of the complex for singlet oxygen generation under these conditions. On the other hand, **Ru3** appears to be more effective than [Ru(bpy)_3_](PF_6_)_2_, increasing from a ratio of 0.3 to 0.7 after 60 seconds of irradiation (comparing [Ru(bpy)_3_](PF_6_)_2_ and **Ru3**, respectively), but remains significantly less effective than the **Ru1** and **Ru2** complexes. The curve for **Ru2** shows a significant increase in ^1^O_2_ production, rising from 0.7 to 1.2 after 60 seconds of irradiation. The curve for **Ru1** shows faster kinetics, reaching its highest value after 60 seconds of irradiation (2.1). Thus, **Ru1** is the most efficient complex for generating ^1^O_2_ via a type II mechanism. This study demonstrated the variable efficiency of our complexes with an order of ^1^O_2_ generation capacity (**Ru1** > **Ru2** > **Ru3** >> [Ru(bpy)_3_](PF_6_)_2_) via energy transfer, which reverses ROS production via electron transfer. Consequently, these results highlight that a third ligand structure with an extended electron density close to the nucleus is favorable for the development of structures that allow the emergence of EnT processes.

**Application to reactions under SET mechanism:** The study of complexes **Ru1** to **Ru3** demonstrated the effectiveness of the complexes in carrying out reactions based on a SET mechanism, with **Ru3** showing greater effectiveness. The ability of the complexes to carry out photocatalyzed reactions via a SET mechanism was evaluated through an alpha-arylation reaction (Table 2).

**Table 2:** Conditions of reactions catalyzed by complexes **Ru1–3** under irradiation at 456 nm. The conversion was determined by LCMS analysis, using of an internal standard, biphenyl, at 0.1 M.

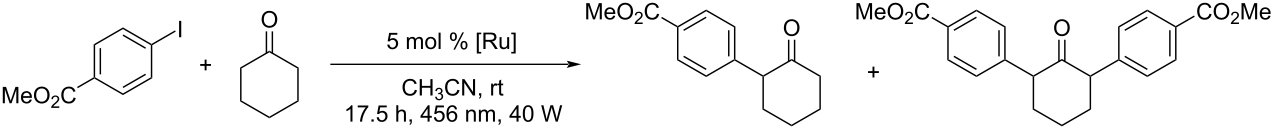			

entry	conditions	% iodide SM^a^	% global conversion

1	w/o catalyst	100	0
2	[Ru(bpy)_3_](PF_6_)_2_	11	89
3	**Ru1**	71	29
4	**Ru1**, w/o light	100	0
5	**Ru2**	76	24
6	**Ru2**, w/o light	100	0
7	**Ru3**	14	86
8	**Ru3**, w/o light	100	0

^a^The starting material is methyl 4-iodobenzoate ester. w/o corresponds to without. Global conversion refers to conversion in mono and di-functionalized ketone.

Alpha-arylation of ketones by photocatalysis is a reaction described in 2022 by Hossain under green light irradiation [78]. In 2024, this reaction was monitored in the presence of a photosensitizer irradiated under blue light [79]. The α-arylation of cyclohexanone with methyl 4-iodobenzoate ester was carried out at 0.1 M in the presence of 5 mol % of [Ru] catalysts under irradiation at 456 nm (Table 2). After 17.5 hours of irradiation, comparison of the experiments monitored under irradiation with the control and without irradiation confirms the ability of the complexes to carry out SET-photocatalyzed reactions. Furthermore, **Ru3** achieved the best conversion rates of 22% and 64% in mono- and disubstituted products, respectively, under irradiation at 456 nm. This confirms the effectiveness of **Ru3** vs **Ru1** and **Ru2** as a photocatalyst in reactions monitored by a SET mechanism.

## Conclusion

In this paper, six new ruthenium polypyridyl complexes based on a third benzimidazole or benzobisimidazole ligand were successfully synthesized. The study of their optical and electronic properties revealed that the structural nature of these ligands significantly influences the MLCT-type excited states as well as the electronic delocalization within the complexes. In particular, the absorption and emission spectra highlighted the impact of the ligand structure on the photophysical properties of the systems studied. Furthermore, the introduction of bridging bisimidazole ligands allowed for a more detailed exploration of binuclear architectures and showed that ligands **L4** to **L6** strongly influence the luminescence and quantum yield of the corresponding complexes.

The study of type I and II reactive oxygen species (ROS) generation also highlighted the ability of complexes **Ru1** to **Ru3** to efficiently produce ROS. The results suggest that the electronic distribution within the complexes plays a decisive role in the mechanism involved in the generation of these species, either by energy transfer to oxygen leading to the formation of singlet oxygen (^1^O_2_), or by electron transfer to an acceptor molecule. In an EnT energy transfer mechanism, the **Ru1** complex proved to be particularly effective in generating ^1^O_2_, while **Ru3** appears to be the most promising candidate in SET mechanisms.

## Supporting Information

File 1 General materials and methods, general synthesis procedures of ligands and complexes, a description of the optical and electrochemical characterization of ligands and complexes, reaction conditions for the ROS generation, as well as all analytical data related to the study.

## Data Availability

All data that supports the findings of this study is available in the published article and/or the supporting information of this article.
