# Supplementary material for: Design and characterization of novel pyridylbenzimidazole-based ruthenium(II) complexes, and their evaluation in singlet oxygen generation
Source: Beilstein J Org Chem. 2026 Nov 4;22:1553–66. doi: 10.3762/bjoc.22.126 (PMC13746521; doi:10.3762/bjoc.22.126)
Supplement: Supplementary file 1 [file Beilstein_J_Org_Chem-22-1553-s001.pdf]

BEILSTEIN JOURNAL OF ORGANIC CHEMISTRY

Supporting Information

for

Design and characterization of novel pyridylbenzimidazole-based ruthenium(II) complexes, and their evaluation in singlet oxygen generation

Benjamin Riss Yaw, Anna Porcher, Alice Saunier, Jean-Christophe Cintrat and Eugénie Romero

Beilstein J. Org. Chem. **2026,** *22,* 1553–1566. doi:10.3762/bjoc.22.126

General materials and methods, general synthesis procedures of ligands and complexes, a description of the optical and electrochemical characterization of ligands and complexes, reaction conditions for the ROS generation, as well as all analytical data related to the study

License and Terms: This is a supporting information file under the terms of the Creative Commons Attribution License (https://creativecommons.org/licenses/by/4.0). Please note that the reuse, redistribution and reproduction in particular requires that the author(s) and source are credited and that individual graphics may be subject to special legal provisions.
The license is subject to the *Beilstein Journal of Organic Chemistry* terms and conditions: (https://www.beilstein-journals.org/bjoc/terms)

Table of content

Materials and equipment S2

Synthesis of ligands S3

Synthesis of ligand **L1** S3

Synthesis of ligand **L2** S4

Synthesis of ligand **L3** S4

Synthesis of ligand **L4** S5

Synthesis of ligand **L5** S5

Synthesis of ligand **L6** S6

Synthesis of ruthenium complexes S6

Ru1, $[Ru(bpy)_2L1](PF_6)_2$ S7

Ru2, $[Ru(bpy)_2L2](PF_6)_2$ S7

Ru3, $[Ru(bpy)_2L3](PF_6)_2$ S8

Ru4, $[Ru(bpy)_2(L4)Ru(bpy)_2](PF_6)_4$ S9

Ru5, $[Ru(bpy)_2(L5)Ru(bpy)_2](PF_6)_4$ S9

Ru6, $[Ru(bpy)_2(L6)Ru(bpy)_2](PF_6)_4$ S10

Cyclic voltammetry S11

Absorbance and fluorescence spectra S14

Ligands S14

Ruthenium complexes S16

Study of the photostability S19

Study of the ROS generation S21

The singlet oxygen generation S21

The ROS generation by a SET mechanism S22

Model photo-reaction by SET mechanism S24

Spectra S26

Materials and equipment

Reactants and solvents

Unless otherwise noted, all reactions were carried out in oven-dried glassware.

Commercially available chemicals were purchased from Acros Organics, Sigma-Aldrich, Alfa Aesar, BLDpharm, Fluorochem, and TCI Europe and used as received unless otherwise stated.

Reactions were monitored by thin-layer chromatography (TLC) carried out on silica gel (60 F254, Merck) using UV light as developer. For staining, the TLC plates were dipped in a basic aqueous permanganate solution (1 g $KMnO_4$, 6 g K_2CO_3 and 0.1 g KOH in 100 mL H_2O) and developed with a heat gun.

Purifications

Column chromatography was performed on silica gel (60 Å, particle size 40–63 µm) or alumina neutral gel.

NMR analysis

^{1}H NMR (400 MHz) and ^{13}C NMR (100 MHz) were recorded at 295 K on a Brucker Avance 400 MHz spectrometer. Chemical shifts (δ) were reported in parts per million (ppm) and coupling constants were reported as Hertz (Hz). The following abbreviations were used for the proton signal multiplicities: s: singulet, d: doublet, t: triplet, q: quartet, p: quintet, h: sextet, m: multiplet, br: broad.

LC-MS analysis

LC-MS spectra were recorded on a Waters Acquity UPLC® equipped PDA eλ Detector and SQ Detector 2, mobile phase A: H_2O + 0.1% formic acid, mobile phase B: acetonitrile + 0.1% formic acid.

HRMS analysis

High-resolution mass spectra (HRMS) were performed on a Waters Xevo® G2-XS QTof mass spectrometer.

Photophysical analysis

UV-Visible absorption spectra were measured on a Cary 50 spectrophotometer from VARIAN in quartz cuvette 1 cm or on a Molecular Device SpectraMax M5e in a 96-well plate.

Fluorescence analysis were performed on a Fluoromax-4 from Horiba Jobin Yvon in quartz cuvette 1 cm.

Electrochemical analysis

The oxidation–reduction behaviour was determined by cyclic voltammetry using a potentiostat (Origaflex OGF500). A glass carbon working electrode, a platinum wire counter electrode, and an $Ag/AgNO_3$ reference electrode were used for CV studies. All solutions were deoxygenated with N_2 and measurements were made in $TBABF_4$ dissolved in acetonitrile and calibrated using ferrocene as a reference (vs couple Fc/Fc^+).

Photobox set-up

The photocatalytic reactions were run using a Kessil® PR160-427 nm LED. The photobox set-up is composed of a plexiglass box covered with an inactive orange lab film. Magnetic stirring plates were placed inside the photobox, which is fitted with fans. The top and back of the box were left open to allow for optimal air circulation. The reactions were run in 4 mL vials equipped with magnetic stir bar, placed at 3 cm from the LEDs.

Synthesis of ligands

General procedure of synthesis of **L1**-**6** ligands:

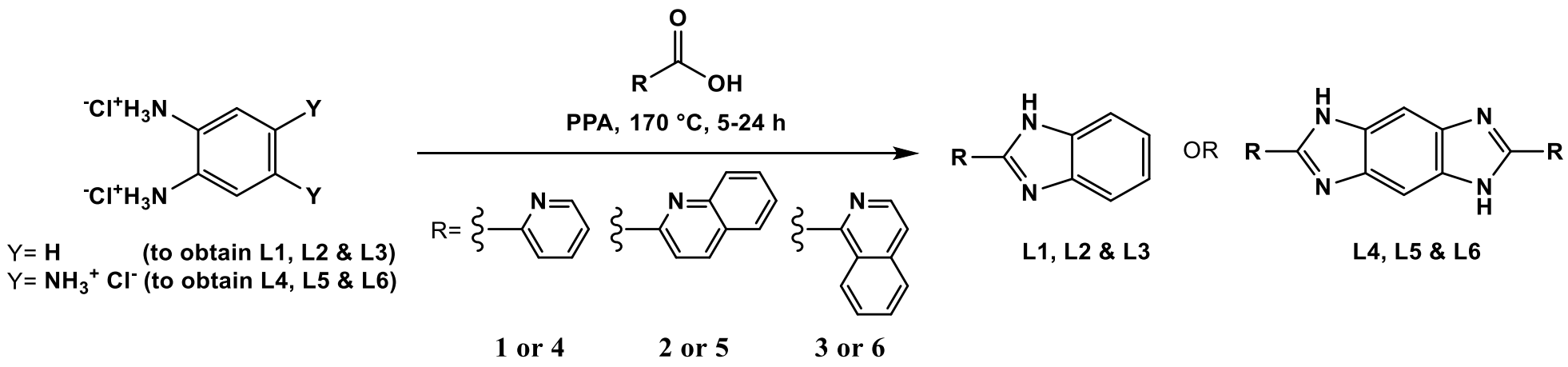

Scheme S1. Synthetic scheme of Ligands (**L1–L6**).

Carboxylic acid (1.0 to 4.0 equiv) and *o*-phenylenediamine chloride (1 equiv, 0.6 mM) dissolved in syrupy phosphoric acid, were stirred at 170 °C for 5–24 hours. The mixture was poured into a beaker of cold water (125 mL/mmol diamine) under vigorous stirring. After cooling, the bulky precipitate was collected by filtration, washed with water (3 times), then with a minimal amount of methanol, before being dried under reduced pressure. The resulting product was suspended in a minimum of 10% aqueous sodium carbonate solution until a neutral pH was achieved. It was then filtered via a fritted glass funnel, washed with water (3 times), with a minimal amount of methanol (3 times), and with dichloromethane (3 times), before being dried under reduced pressure to give ligands **L** as a white or brown powder.

Synthesis of ligand L1

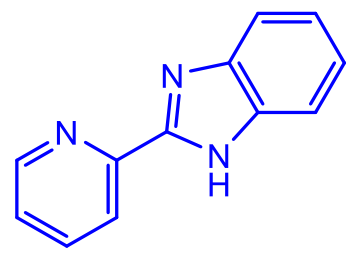

2-(pyridin-2-yl)-1*H*-benzo[*d*]imidazole
L1

L1 was prepared from 103.7 mg of quinoline-2-carboxylic acid (0.84 mmol, 1.1 equiv) and 143.1 mg of *o*-phenylenediamine chloride (0.79 mmol, 1 equiv) in 1.2 mL of syrupy phosphoric acid according to general procedure to yield **L1** as a white powder (25 mg, 16% yield) after 5 h. Data are consistent with the literature [1].

R_f: 0.37 (3/7 Hexane/EtOAc).

^{1}H NMR (400 MHz, DMSO-d$_6$): 13.10 (br s, 1H), 8.74 (br d, 1H, ^3J= 3.7 Hz), 8.34 (br d, 1H, ^3J= 7.8 Hz), 8.00 (br t, 1H, ^3J = 7.8 Hz, ^3J = 7.4 Hz), 7.71 (br d, 1H, ^3J = 7.4 Hz), 7.59-7.46 (m, 2H), 7.29-7.16 (m, 2H).

^{13}C NMR (101 MHz, DMSO-d$_6$): 150.7 (C$_Q$), 149.4 (C), 148.5 (C$_Q$), 143.9 (C$_Q$), 137.6 (C), 134.9 (C$_Q$), 124.7 (C), 123.1 (C), 121.9 (C), 121.4 (C), 119.3 (C), 112.1 (C).

LC-MS (ESI+) m/z: calculated for $C_{12}H_{10}N_3$ [M+H]$^+$: 196, found: 196.

[1] Schiffman, R.; Neugebauer, A.; Klein, C. D. *J. Med. Chem.* **2006**, *49 (2)*, 511-522.

Synthesis of ligand L2

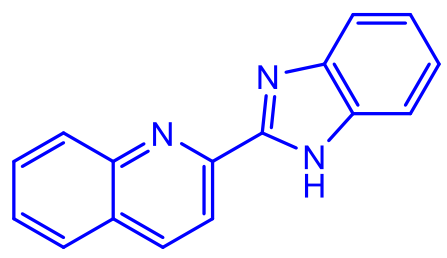

2-(1*H*-benzo[*d*]imidazol-2-yl)quinoline
L2

L2 was prepared from 117.2 mg of quinoline-2-carboxylic acid (0.679 mmol, 1.2 equiv) and 104.5 mg of *o*-phenylenediamine chloride (0.577 mmol, 1 equiv) in 1 mL of syrupy phosphoric acid according to general procedure to yield **L2** as a white powder (118 mg, 83% yield) after 24 h. Data are consistent with the literature [2].

R_f: 0.41 (3/7 Hexane/EtOAc).

^{1}H NMR (400 MHz, DMSO-d_6): 13.24 (br s, 1H), 8.55 (d, 1H, ^3J = 8.6 Hz), 8.49 (d, 1H, ^3J = 8.6 Hz), 8.17 (br d, 1H), 8.07 (br d, 1H), 7.87 (br t, 1H), 7.76-7.62 (m, 3H), 7.33-7.21 (2br d, 2H).

^{13}C NMR (101 MHz, DMSO-d_6): 150.8 (C_Q), 148.8 (C_Q), 147.2 (3C_Q), 137.4 (C), 130.5 (C), 128.8 (C), 128.3 (C), 128.1 (C_Q), 127.3 (3C), 122.8 (2C), 119.3 (C).

LC-MS (ESI+) m/z: calculated for $C_{16}H_{12}N_3$ $[M+H]^+$: 246, found: 246.

Synthesis of ligand L3

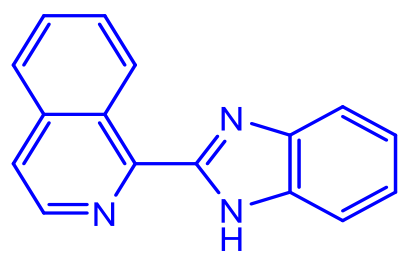

1-(1*H*-benzo[*d*]imidazol-2-yl)isoquinoline
L3

L3 was prepared from 98.2 mg of isoquinoline-2-carboxylic acid (0.550 mmol, 1 equiv) and 99.5 mg of *o*-phenylenediamine chloride (0.567 mmol, 1 equiv) in 0.6 mL syrupy phosphoric acid according to general procedure to yield **L3** as a yellow powder (98.4 mg, 73% yield) after 24 h. Data are consistent with the literature [3].

R_f: 0.62 (3/7 Hexane/EtOAc).

^{1}H NMR (400 MHz, DMSO-d_6): 13.23 (br s, 1H), 10.14-10.08 (br ddd, 1H), 8.71 (d, 1H, ^3J = 8.6 Hz), 8.118.07 (br ddd, 1H), 8.01 (d, 1H, ^3J = 8.6 Hz), 7.90-7.81 (m, 3H), 7.61 (br dt, 1H, ^3J = 8.0 Hz), 7.32 (td, 1H, ^3J = 8.0 Hz), 7.26 (td, 1H, ^3J = 8.0 Hz).

^{13}C NMR (101 MHz, DMSO-d_6): 151.2 (C_Q), 146.7 (C_Q), 144.1 (C_Q), 141.7 (C), 136.8 (C_Q), 134.0 (C_Q), 130.7 (C), 128.7 (C), 127.8 (C), 127.3 (C), 126.0 (C_Q), 123.7 (C), 122.4 (C), 122.0 (C), 119.7 (C), 112.1 (C).

LC-MS (ESI+) m/z: calculated for $C_{16}H_{12}N_3$ $[M+H]^+$: 246, found: 246.

---

[2] Nguyen, T. B.; Ermolenko, L.; Al-Mourabit, A. *J. Am. Chem. Soc.* **2013**, *135 (1)*, 118-121.
[3] Cosimelli, B.; Taliani, S.; Greco, G.; Novellino, E.; Sala, A.; Severi, E.; Da Settimo, F.; La Motta, C.; Pugliesi, I.; Antonioli, L.; Fornai, M.; Colucci, R.; Blandizzi, C.; Daniele, S.; Trincavelli, M. L.; Martini, C. *ChemMedChem.* **2011**, *6 (10)*, 1909-1918.

Synthesis of ligand L4

syn **anti**

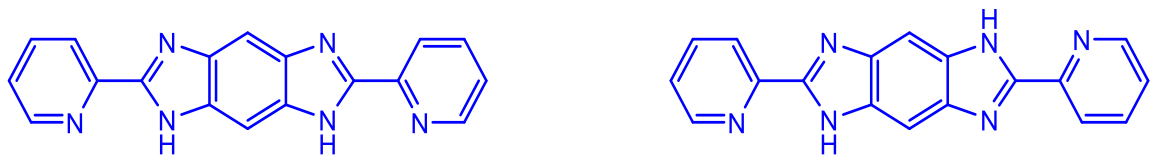

2,6-di(pyridin-2-yl)-1,7-dihydrobenzo[1,2-*d*:4,5-*d'*]diimidazole
L4 (ratio: 0.5/0.5 syn/anti)

L4 was prepared from 88.4 mg of quinaldic acid (0.72 mmol, 2.2 equiv) and 101.0 mg of *o*-phenylenediamine chloride (0.356 mmol, 1 equiv) in 2 mL of syrupy phosphoric acid according to general procedure to yield **L4** as a dark pink/orange powder (114 mg, quantitative yield) after 24 h. Data are consistent with the literature [4].

^{1}H NMR (400 MHz, DMSO-d$_6$): 12.92 (br s, 2H), 8.74 (br d, 2H, ^3J = 4.4 Hz), 8.35 (br d, 2H, ^3J = 7.7 Hz), 8.01 (br dd, 2H, ^3J = 7.7 Hz, ^3J = 7.4 Hz), 7.93 (s, 0.5xH), 7.74 (s, 1H), 7.59 (s, 0.5xH), 7.56-7.49 (m, 2H).

^{13}C NMR (101 MHz, DMSO-d$_6$): 151.6 (2C$_Q$), 151.2 (2C$_Q$), 149.4 (C), 148.6 (2C$_Q$), 142.7 (C$_Q$), 141.5 (C$_Q$), 137.6 (C), 137.5 (C), 133.5 (C), 124.6 (C), 121.4 (C), 121.3 (C), 107.6 (C), 99.4 (C), 92.4 (C).

LC-MS (ESI+) m/z: calculated for $C_{18}H_{13}N_6$ $[M+H]^+$: 313, found: 313.

Synthesis of ligand L5

syn **anti**

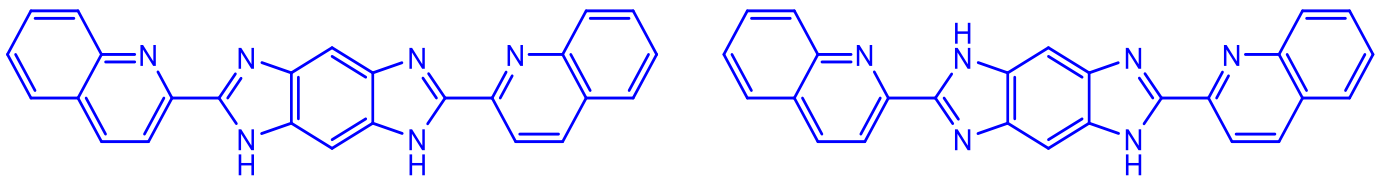

2,6-di(quinolin-2-yl)-1,7-dihydrobenzo[1,2-*d*:4,5-*d'*]diimidazole
L5 (ratio: 0.5/0.5 syn/anti)

L5 was prepared from 128 mg of quinaldic acid (0.81 mmol, 2.3 equiv) and 104.9 mg of *o*-phenylenediamine chloride (0.37 mmol, 1 equiv) in 2.2 mL of syrupy phosphoric acid according to general procedure to yield **L5** as a dark pink/orange powder (122 mg, quantitative yield) after 5 h.

^{1}H NMR (400 MHz, DMSO-d$_6$): 13.07 (s, 2H), 8.58 (d, 1H, $^3J_{H11\text{-}H12}$ = 8.6 Hz), 8.53(dd, 2H, ^3J = 8.6 Hz, ^3J = 3.0 Hz), 8.18 (br d, 2H), 8.08 (m, 3H), 7.88 (m, 3H), 7.75 (s, 0.5xH), 7.69 (br td, 2H).

^{13}C NMR (101 MHz, DMSO-d$_6$): 151.4 (C$_Q$), 148.8 (C$_Q$), 148.7 (C$_Q$), 147.3 (C$_Q$), 142.7 (C$_Q$), 141.7 (C$_Q$), 137.4 (2C), 134.2 (C$_Q$), 133.2 (C$_Q$), 130.5 (2C), 128.8 (2C$_Q$), 128.3(C), 128.1 (C), 127.3 (2C), 119.3 (2C), 108.1 (C), 100.1 (2C), 92.7 (C).

LC-MS (ESI+) m/z: calculated for $C_{26}H_{17}N_6$ $[M+H]^+$: 413, found: 413.

[4] Edzang, J. A.; Chen, Z.; Audi, H.; Canard, G.; Siri, O. *Org. Lett.* **2016**, *18 (20)*, 5340-5343.

Synthesis of ligand L6

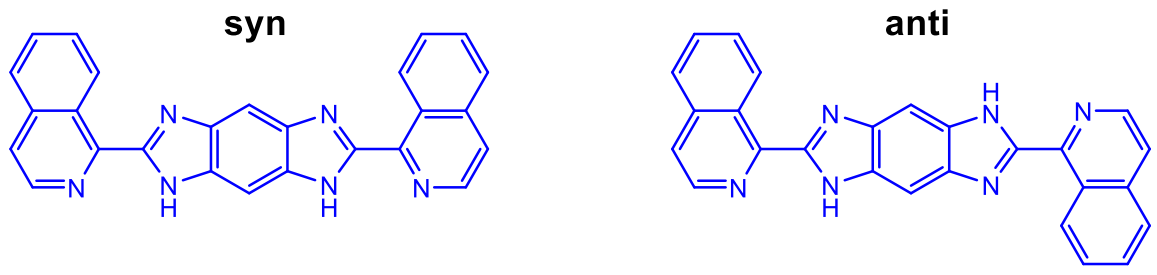

2,6-di(isoquinolin-1-yl)-1,7-dihydrobenzo[1,2-*d*:4,5-*d'*]diimidazole
L6 (ratio: 0.5/0.5 syn/anti)

L6 was prepared from 138.6 mg of isoquinoline-1-carboxylic acid (0.80 mmol, 4.0 equiv) and 57.0 mg of *o*-phenylenediamine chloride (0.20 mmol, 1 equiv) in 2mL of syrupy phosphoric acid according to general procedure to yield **L6** as a dark brown powder with a purple glint (33.2 mg, 40 % yield) after 5 h.

^{1}H NMR (400 MHz, DMSO-d$_6$): 13.10 (s, 2H), 10.28-10.17 (m, 2H), 8.73 (d, 2H, ^3J = 5.5 Hz), 8.24 (s, 0.5xH), 8.13-8.08 (m, 2H), 8.01 (br d, 2H, ^3J = 5.5 Hz), 7.94 (s, 1H), 7.92-7.86 (m, 4H), 7.69 (s, 0.5xH).

^{13}C NMR (101 MHz, DMSO-d$_6$): 152.3 (C$_Q$), 151.9 (C$_Q$), 146.6 (C$_Q$), 143.0 (C$_Q$), 142.0 (C$_Q$), 141.8 (C), 141.7 (C), 136.8 (C$_Q$), 133.1 (C$_Q$), 130.7 (C), 128.7 & 128.6 (2C), 128.0 (C), 127.3 (2C), 126.1 (C$_Q$), 126.0 (C$_Q$), 122.3 (2C), 108.5 (C), 100.0 (C), 92.1 (C).

LC-MS (ESI+) m/z: calculated for $C_{26}H_{17}N_6$ $[M+H]^+$: 413, found: 413.

Synthesis of ruthenium complexes

General procedure of synthesis for **Ru1-6** complexes:

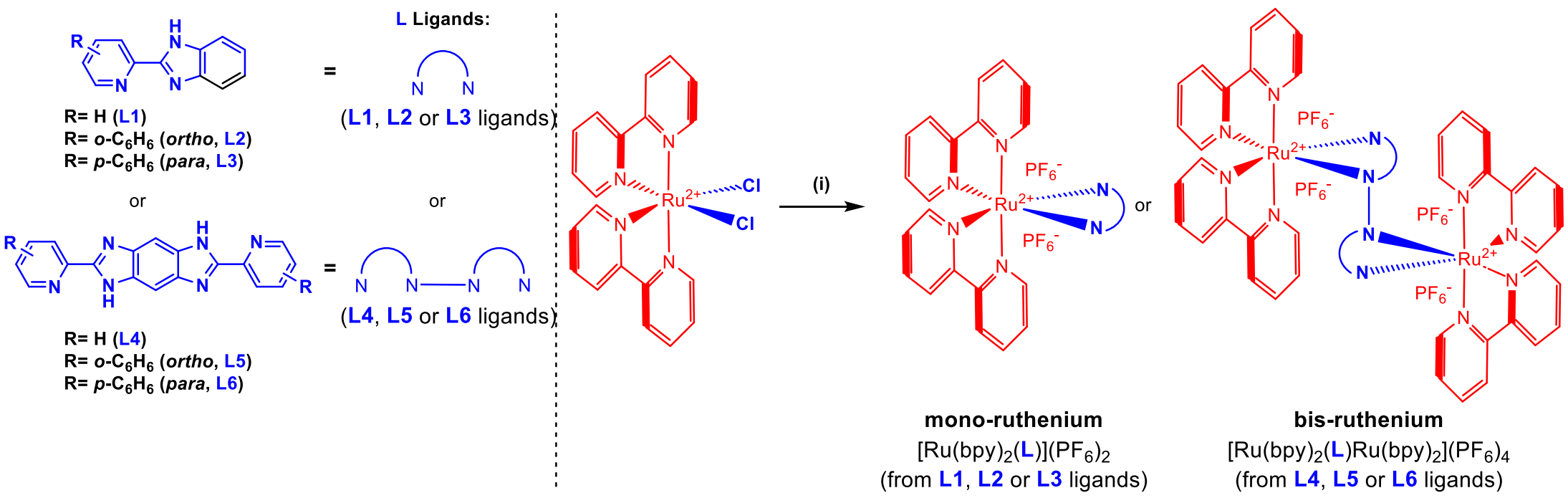

Scheme S2. Synthetic scheme of ruthenium complexes (**Ru1-Ru6**). (i) Ligands (**L1-L6**), 2-ethoxyethanol, microwave, 190 W, 197 °C, 20 minutes.

1 equiv of ligand **L** (0.060 mM) and 1 equiv (to afford mono-ruthenium) or 2 equiv (to afford bisruthenium) of $[Ru(bpy)_2]Cl_2$ were suspended in 2-ethoxyethanol before being heated in a micro-wave at 197 °C during 20 min. After 20 minutes of middle stirring, the red/orange solution was evaporated before being suspended in water and precipitated by adding a cold saturated aqueous solution of KPF_6. The red precipitate was filtered via a fritted glass funnel, washed with water (3 times) and rinsed with diethyl ether (3 times). The dark red precipitate was dissolved in DCM and the DCM filtrate was evaporated under reduced pressure. The resulting product was purified by chromatography on neutral alumina column (elution with ACN/DCM, 30/70) to give ruthenium complexes **Ru** (26 to 70 % yields) as a red/orange ochre powder after lyophilization.

Ru1, $[Ru(bpy)_2L1](PF_6)_2$

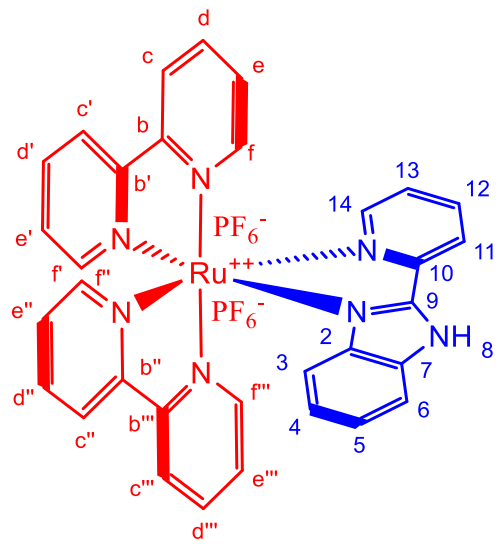

Ru1

Ru1 was prepared from 30.2 mg of **L1** (0.155 mmol, 1 equiv) and 89 mg of $[Ru(bpy)_2]Cl_2$ (0.184 mmol, 1.2 equiv) suspended in 2.6 mL of 2-ethoxyethanol according to general procedure to yield **Ru1** as a red/orange ochre powder (97.6 mg, 70% yield) after 20 minutes.

R_f: 0.25 (3/7 CH_3CN/CH_2Cl_2).

^{1}H NMR (400 MHz, DMSO-d$_6$): 8.84-8.77 (m, 3H), 8.72 (d, 1H, ^3J = 8.0 Hz), 8.33 (br d, 1H, ^3J = 8.0 Hz), 8.18 (td, 1H, ^3J = 8.0 Hz, ^4J = 1.5 Hz), 8.12 (td, 1H, ^3J = 8.0 Hz, ^4J = 1.5 Hz), 8.06 (td, 1H, ^3J = 8.0 Hz, ^4J = 1.5 Hz), 8.00-7.96 (m, 1H), 7.97-7.92 (m, 1H), 7.89 (br d, 1H, ^3J = 8.0 Hz), 7.83 (br d, 1H, ^3J = 8.0 Hz), 7.57-7.45 (m, 5H), 7.56-7.50 (m, 1H), 7.50-7.45 (m, 1H), 7.28 (td, 1H, ^3J = 7.3 Hz, ^4J = 1.5 Hz), 6.90 (td, 1H, ^3J = 8.1 Hz, ^4J = 1.0 Hz), 6.61 (td, 1H, ^3J = 8.1 Hz, ^4J = 1.0 Hz), 5.45 (dt, 1H, ^3J = 8.1 Hz, ^4J = 1.0 Hz).

^{13}C NMR (101 MHz, DMSO-d$_6$): 159.0 (C_Q), 158.0 (C_Q), 157.7 (C_Q), 157.0 (2C_Q), 155.1 (C_Q), 151.8 (C), 151.4 (C), 150.9 (C), 150.8 (C), 150.3 (C), 147.4 (C_Q), 144.9 (C_Q), 137.4 (C), 136.8 (2C), 136.7 (2C), 127.7 (C), 127.4 (2C), 126.9 (C), 124.7 (C), 124.3 (C), 124.0 (C), 123.8 (C), 123.7 (C), 121.6 (C), 120.8 (C), 120.0 (C), 119.3 (C), 112.4 (C).

HRMS (ESI-TOF) m/z: calculated for $C_{32}H_{24}N_7Ru$ $[M-2PF_6]^{2+}$: 304.5612, found 304.5613.

HRMS (ESI-TOF) m/z: calculated for $C_{32}H_{23}N_7Ru$ $[M-H-2PF_6]^{+}$: 608.1145, found 608.1144.

Ru2, $[Ru(bpy)_2L2](PF_6)_2$

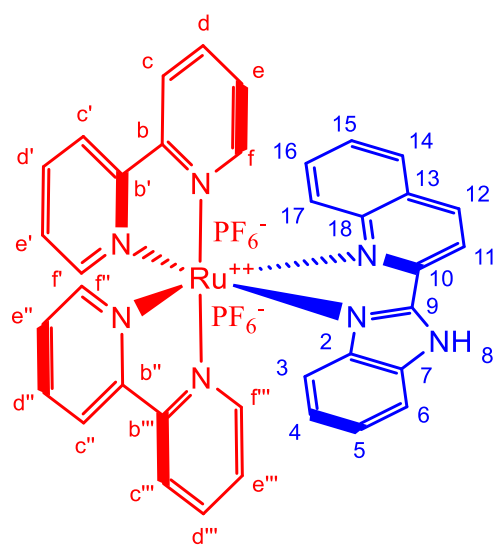

Ru2

Ru2 was prepared from 26.0 mg of **L2** (0.106 mmol, 1 equiv) and 85.0 mg of $[Ru(bpy)_2]Cl_2$ (0.175 mmol, 1.65 equiv) was suspended in 2 mL of 2-ethoxyethanol according to general procedure to yield **Ru2** as an orange powder (46.8 mg, 46% yield) after 20 minutes.

R_f: 0.68 (3/7 CH_3CN/CH_2Cl_2).

1**H NMR (400 MHz, DMSO-d$_6$):** 15.14 (br s, 1H), 8.92 (d, 1H, ^3J = 8.0 Hz), 8.85 (d, 1H, ^3J = 8.0 Hz), 8.76 (2d, 2H, ^3J = 8.1 Hz, ^3J = 8.0 Hz,), 8.66 (2d, 2H, ^3J = 8.1 Hz, ^3J = 8.0 Hz), 8.27 (t, 1H, ^3J = 8.0 Hz), 8.21-8.15 (t, 1H, ^3J = 8.0 Hz), 8.19-8.12 (m, 1H), 8.15-8.08 (m, 2H), 8.06 (d, 1H, ^3J = 8.0 Hz), 7.99 (t, 1H, ^3J = 8.0 Hz), 7.85 (d, 1H, ^3J = 8.0 Hz), 7.81 (d, 1H, ^3J = 8.1 Hz), 7.66-7.58 (m, 2H), 7.53 (t, 1H, ^3J = 8.0 Hz), 7.48-7.43 (m, 2H), 7.48-7.43 (m, 1H), 7.32 (m, 2H), 7.23 (d, 1H, ^3J = 8.8 Hz), 6.94 (t, 1H, ^3J = 8.4 Hz, ^3J = 7.3 Hz), 5.42 (d, 1H, ^3J = 8.4 Hz).

13**C NMR (101 MHz, DMSO-d$_6$):** 158.3 (C$_Q$), 157.5 (2C$_Q$), 156.8 (2C$_Q$), 153.3 (C), 152.4 (C), 151.1 (C), 150.8 (C), 149.5 (C$_Q$), 142.7 (C$_Q$), 139.6 (C), 139.5 (C), 138.2 (C), 137.6 (C), 137.3 (C), 137.2 (C), 136.0 (C$_Q$), 131.5 (C), 129.8 (C), 128.8 (C$_Q$), 128.4 (C$_Q$), 128.3 (C$_Q$), 128.1 (C), 127.8 (C), 127.6 (C), 127.6 (C), 124.6 (C), 124.4 (C), 124.2 (C), 124.0 (C), 123.9 (C), 123.8 (C), 119.5 (C), 115.8 (C), 114.3 (C).

HRMS (ESI-TOF) m/z: calculated for $C_{36}H_{27}N_7Ru$ [M-2PF$_6$]$^{2+}$: 329.5691, found 329.5688.

HRMS (ESI-TOF) m/z: calculated for $C_{36}H_{26}N_7Ru$ [M-H-2PF$_6$]$^{+}$: 658.1302, found 658.1306.

Ru3, [Ru(bpy)$_2$L3](PF$_6$)$_2$

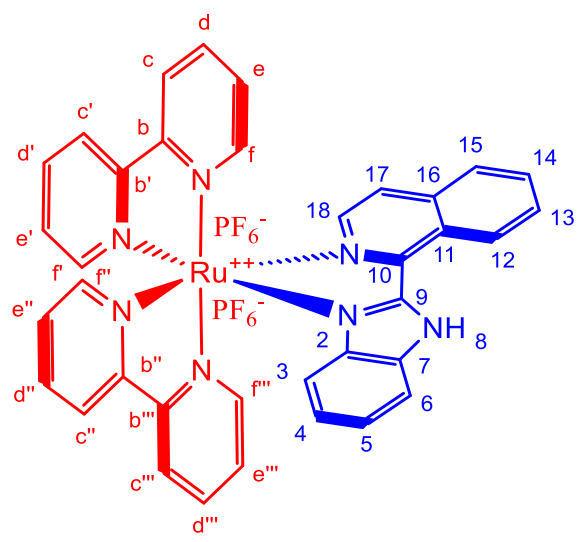

Ru3

Ru3 was prepared from 26.0 mg of **L3** (0.106 mmol, 1 equiv) and 54.0 mg of [Ru(bpy)$_2$]Cl$_2$ (0.111 mmol, 1.05 equiv) were suspended in 2 mL of 2-ethoxyethanol according to general procedure to yield **Ru3** as an orange ochre powder (40.9 mg, 43% yield) after 20 minutes.

R$_f$: 0.34 (3/7 CH_3CN/CH_2Cl_2).

^{1}H NMR (400 MHz, DMSO-d$_6$): 10.70 (d, 1H, ^3J = 8.5 Hz), 8.87-8.81 (2d, 2H, ^3J = 8.2 Hz), 8.79 (d, 1H, ^3J = 8.2 Hz), 8.73 (d, 1H, ^3J = 8.2 Hz), 8.20 (br td, 1H, ^3J = 8.2 Hz), 8.15 (br td, 1H, ^3J = 8.2 Hz), 8.04-7.97 (m, 3H), 7.96-7.92 (m, 2H), 7.88-7.83 (m, 3H), 7.71-7.66 (2d, 2H, ^3J = 8.0 Hz), 7.56 (2br td, 2H, ^3J = 8.2 Hz), 7.46-7.38 (m, 4H) , 6.99 (br dd, 1H, ^3J = 7.4 Hz), 6.67 (br t, 1H, ^3J = 8.2 Hz, ^3J = 7.4 Hz), 5.53 (d, 1H, ^3J =

8.2 Hz).

^{13}C NMR (101 MHz, DMSO-d$_6$): 160.3 (C$_Q$), 157.8 (C$_Q$), 157.5 (C$_Q$), 157.0 (C$_Q$), 156.9 (C$_Q$), 153.8 (C$_Q$), 152.8 (C$_Q$), 151.6 (C), 151.3 (C), 151.1 (C), 150.8 (C), 147.5 (C$_Q$), 144.1 (C$_Q$), 141.8 (C), 136.9 (2C), 136.7 (C), 136.2 (C), 135.5 (C$_Q$), 131.5 (C), 129.2 (C), 127.9 (C), 127.5 (C), 127.3 (C), 127.1 (C), 127.0 (C), 126.6 (C$_Q$), 124.3 (C$_Q$), 124.0 (C), 123.8 (C), 123.7 (C), 122.2 (C), 121.5 (C), 120.5 (C), 119.8 (C), 112.7 (C).

HRMS (ESI-TOF) m/z: calculated for $C_{36}H_{27}N_7Ru$ [M-2PF$_6$]$^{2+}$: 329.5691, found 329.5689.

HRMS (ESI-TOF) m/z: calculated for $C_{36}H_{26}N_7Ru$ [M-H-2PF$_6$]$^{+}$: 658.1302, found 658.1301.

Ru4, [Ru(bpy)$_2$(L4)Ru(bpy)$_2$](PF$_6$)$_4$

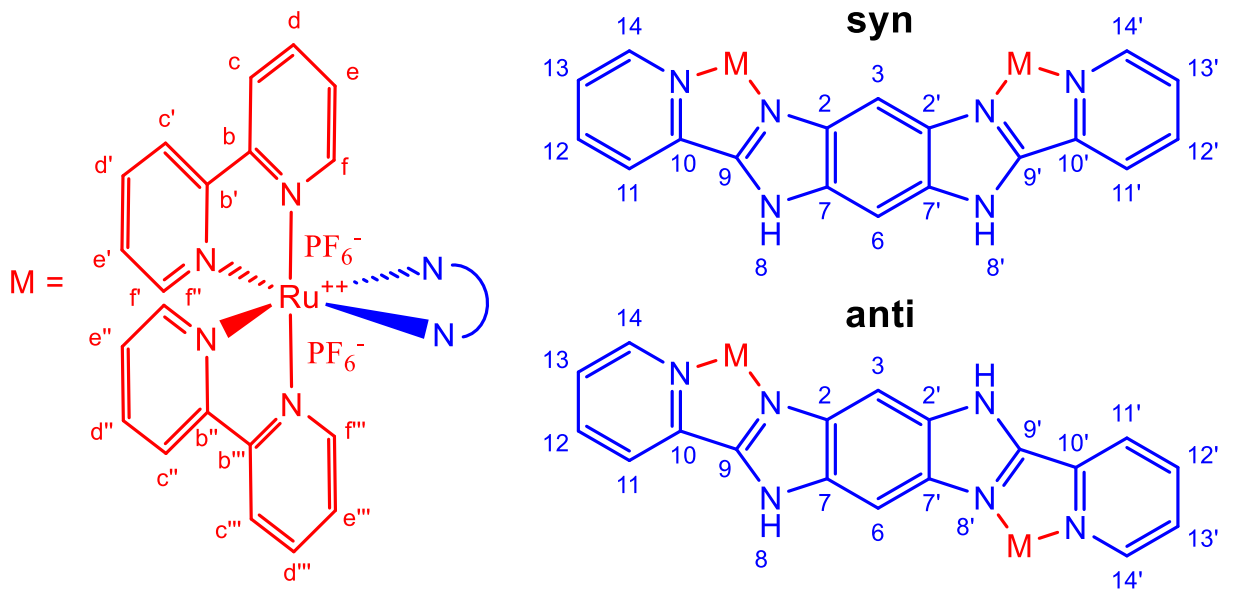

Ru4 (syn/anti)

Ru4 was prepared from 14.2 mg of **L4** (0.045 mmol, 1 equiv) and 45.2 mg of [Ru(bpy)$_2$]Cl$_2$ (0.093 mmol, 2 equiv) were suspended in 1.6 mL of 2-ethoxyethanol according to general procedure to yield **Ru4** as a dark orange solid (53 mg, 69% yield) after 20 minutes.

R$_f$: 0.25 (3/7 CH_3CN/CH_2Cl_2).

^{1}H NMR (400 MHz, DMSO-d$_6$): 14.55 (br s, 2H), 8.91 (d, 1H, ^3J = 8.2 Hz), 8.89-8.77 (m, 7H), 8.71 (br d, 2H, ^3J = 8.2 Hz), 8.57-8.47 (m, 2H), 8.28-8.06 & 8.04-7.99 (2m, 13H), 7.97 (d, 1H, ^3J = 8.2 Hz), 7.85-7.78 & 7.77-7.65 (2m, 13H), 7.57-7.48 & 7.45-7.40 (2m, 9H), 7.34 (m, 1H), 5.89 (m, 2H).

^{13}C NMR (101 MHz, DMSO-d$_6$): 157.8 (C$_Q$), 157.7 (C$_Q$), 157.4 (C$_Q$), 156.7 (C$_Q$), 156.6 (C$_Q$), 154.0 (C$_Q$), 153.9 (C$_Q$), 152.4 (C), 152.0 (C), 151.7 (C), 151.6 (C), 151.6 (C), 148.3 (C), 147.1 (C), 140.4 (C$_Q$), 140.2 (C$_Q$), 138.5 (C), 138.5 (C), 138.5 (C), 138.2 (C), 137.8 (C), 137.7 (C), 137.6 (C), 137.4 (2C$_Q$), 132.7 (C), 128.1 (2C), 128.0 (C), 127.9 (C), 127.8 (C), 127.7 (C), 127.6 (C), 124.7 (C), 124.5 (C), 124.4 (C), 124.2 (C), 124.1 (C), 98.1 (2C).

HRMS (ESI-TOF) m/z: calculated for $C_{58}H_{43}N_{14}Ru_2$ [M-H-4PF$_6$]$^{3+}$: 379.7303, found 379.7298.

HRMS (ESI-TOF) m/z: calculated for $C_{58}H_{42}N_{14}Ru_2$ [M-2H-4PF$_6$]$^{2+}$: 569.0916, found 569.0912.

Ru5, [Ru(bpy)$_2$(L5)Ru(bpy)$_2$](PF$_6$)$_4$

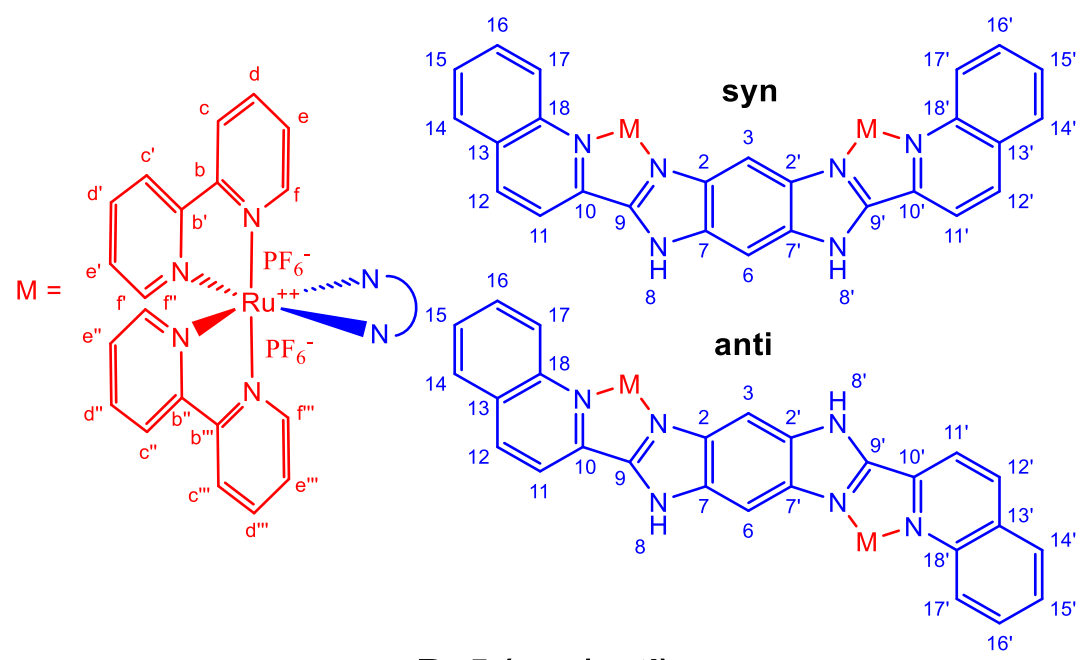

Ru5 (syn/anti)

Ru5 was prepared from 32.5 mg of **L5** (0.073 mmol, 1 equiv) and 70 mg of [Ru(bpy)$_2$]Cl$_2$ (0.145 mmol, 2 equiv) was suspended in 2.5 mL of 2-ethoxyethanol according to general procedure to yield **Ru5** as a dark orange solid (19.5 mg, 18% yield) after 20 minutes.

R$_f$: 0.34 (3/7 CH_3CN/CH_2Cl_2).

^{1}H NMR (400 MHz, DMSO-d$_6$): 9.02 (br d, 1H, ^3J = 8.0 Hz), 8.92 (br d, 2H, ^3J = 8.0 Hz), 8.77 (br d, 1H, ^3J = 8.5 Hz), 8.72 (br d, 1H, ^3J = 8.5 Hz), 8.67 (br d, 1H, ^3J = 8.5 Hz), 8.63 (br d, 1H, ^3J = 8.5 Hz), 8.55 (br d, 1H, ^3J = 8.5 Hz), 8.47-8.37 (m, 4H), 8.36-8.29 (m, 2H), 8.20 (br d, ^3J = 5.5 Hz, 1H), 8.16-8.03 (m, 7H), 8.00-7.90 (m, 5H), 7.89-7.82 (m, 2H), 7.77 (br d, 1H, ^3J = 5.7 Hz), 7.62-7.56 (m, 3H), 7.53 (br t, 1H, ^3J = 8.0 Hz), 7.49-7.42 (m, 3H), 7.42-7.37 (m, 2H), 7.36-7.31 (m, 1H), 7.31-7.28 (m, 1H), 7.28-7.25 (m, 1H), 7.21-7.12 (m, 4H), 7.02 (br d, 1H, ^3J = 5.7 Hz), 5.47 & 5.43 (2s, 2H).

^{13}C NMR (101 MHz, DMSO-d$_6$): 158.6 (C$_Q$), 158.5 (2C$_Q$), 158.4 (C$_Q$), 158.3 (C$_Q$), 157.6 (C$_Q$), 157.2 (C$_Q$), 157.1 (C$_Q$), 157.0 (C$_Q$), 156.6 (3C), 154.5 (2C), 152.8 (C), 152.5 (C), 152.0 (C$_Q$), 151.9 (C$_Q$), 149.3 (C$_Q$), 138.0 (C), 137.9 (C), 137.4 (2C), 136.8 (C), 136.7 (C), 136.5 (2C), 136.0 (C$_Q$), 131.6 (C), 131.5 (C), 130.5 (C), 128.6 (C$_Q$), 128.3 (C$_Q$), 127.9 (2C), 127.3 (C), 127.0 (3C), 126.8 (C), 124.3 (C), 124.2 (C), 124.1 (C), 124.0 (C), 123.9 (C), 123.8 (C), 123.7 (C), 123.6 (C), 123.5 (C), 119.5 (C), 99.3 (C).

HRMS (ESI-TOF) m/z: calculated for $C_{66}H_{46}N_{14}Ru_2$ [M-2H-4PF$_6$]$^{2+}$: 619.1074, found 619.1071.

HRMS (ESITOF) m/z: calculated for $C_{66}H_{47}F_6N_{14}Ru_2$ [M-H-3PF$_6$]$^{+}$: 692.0933, found 692.0931.

Ru6, [Ru(bpy)$_2$(L6)Ru(bpy)$_2$](PF$_6$)$_4$

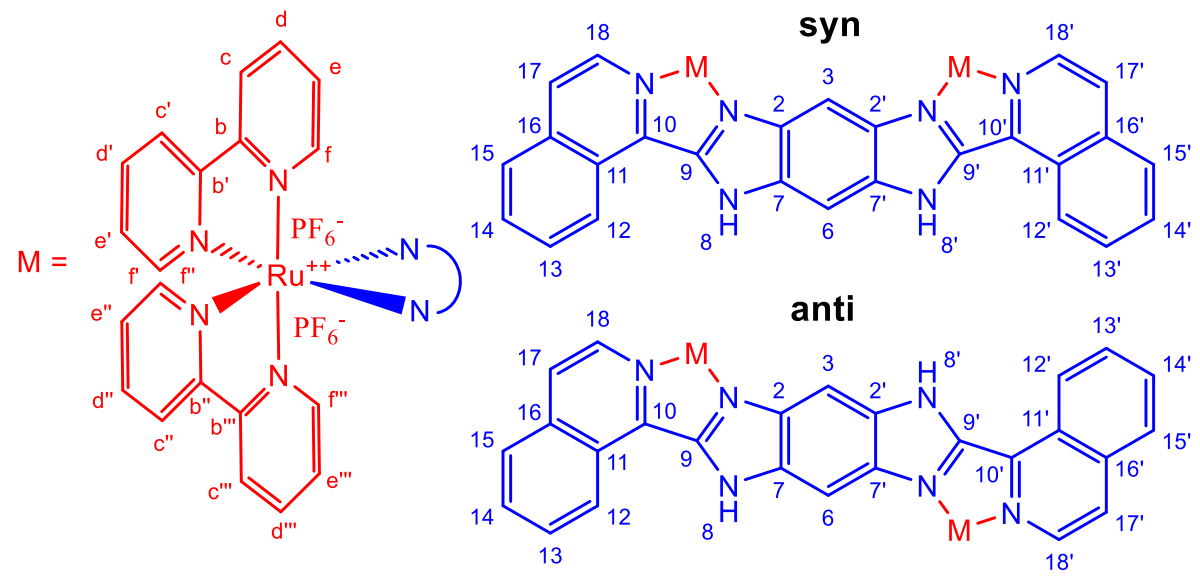

Ru6 (syn/anti)

Ru6 was prepared from 27 mg of **L6** (0.065 mmol, 1 equiv) and 70 mg of [Ru(bpy)$_2$]Cl$_2$ (0.145 mmol, 2 equiv) were suspended in 2.6 mL of 2-ethoxyethanol according to general procedure to yield **Ru6** as a dark orange solid (65 mg, 56% yield) after 20 minutes.

R$_f$: 0.46 (3/7 CH_3CN/CH_2Cl_2).

^{1}H NMR (400 MHz, DMSO-d$_6$): 10.5 (m, 2H), 8.96 (br d, 2H), 8.92-8.72 (m, 10H), 8.69 (br d, 1H), 8.36-8.27 (m, 1H), 8.18-8.11 (m, 2H), 8.07-7.99 (m, 3H), 7.99-7.93 (m, 3H), 7.93-7.77 (m, 6H), 7.77-7.72 (m, 2H), 7.72-7.63 (m, 2H), 7.63-7.52 (m, 4H), 7.48-7.38 (m, 2H), 7.38-7.25 (m, 2H), 5.86 (s, 1H), 5.79 (s, 1H).

^{13}C NMR (101 MHz, DMSO-d$_6$): not obtained as a result of poor solubility.

HRMS (ESI-TOF) m/z: calculated for $C_{66}H_{47}N_{14}Ru_2$ [M-H-4PF$_6$]$^{3+}$: 413.0742, found 413.07545.

HRMS (ESITOF) m/z: calculated for $C_{66}H_{46}N_{14}Ru_2$ [M-2H-4PF$_6$]$^{2+}$: 619.1074, found 619.1074.

Cyclic voltammetry

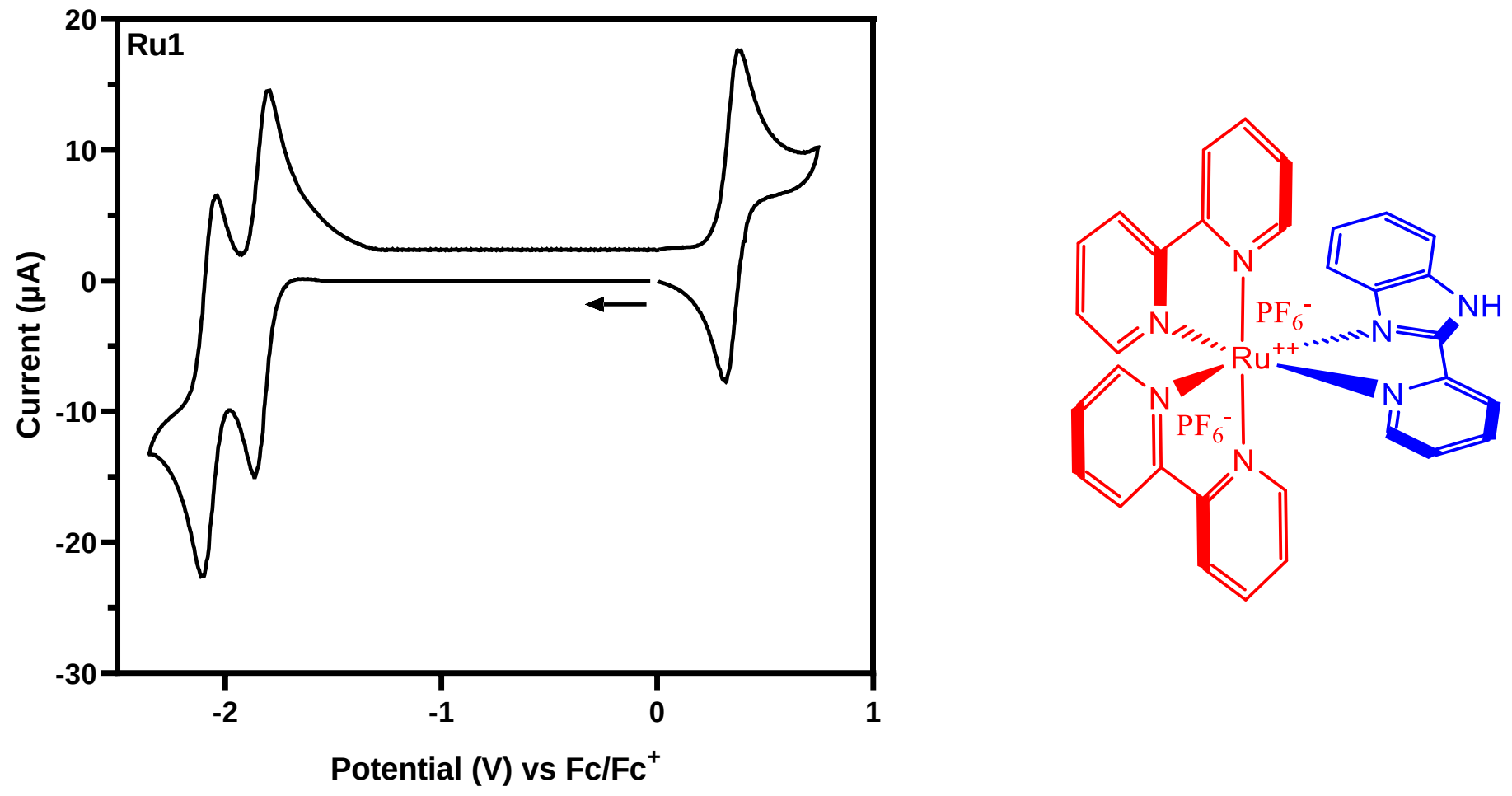

Figure S1. Cyclic voltammogram of **Ru1** (2 mM) in 0.1 M Bu_4NPF_6 ACN electrolyte, scan rate 0.3 V/s.

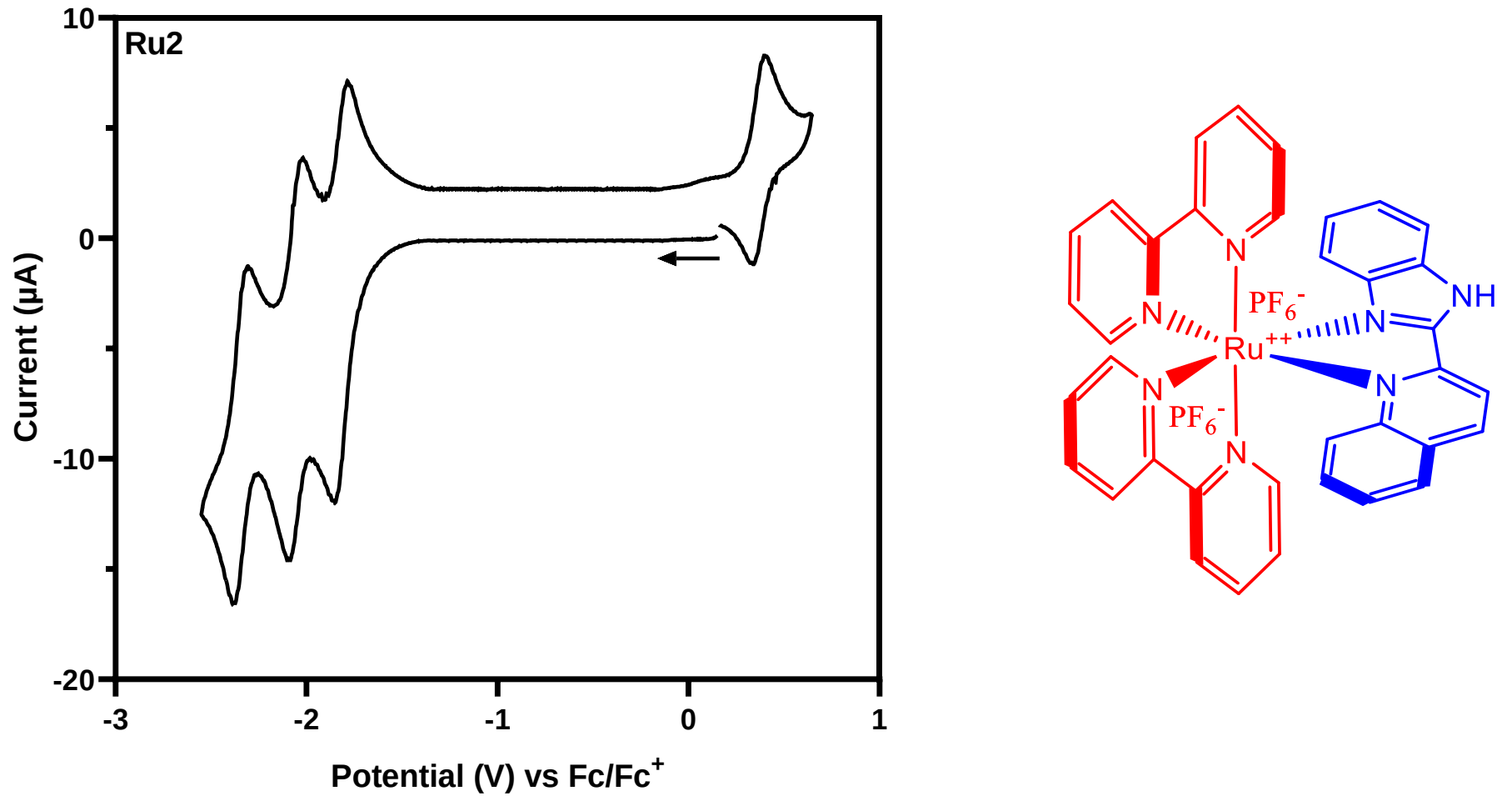

Figure S2. Cyclic voltammogram of **Ru2** (2 mM) in 0.1 M Bu4NPF6 ACN electrolyte, scan rate 0.3 V/s.

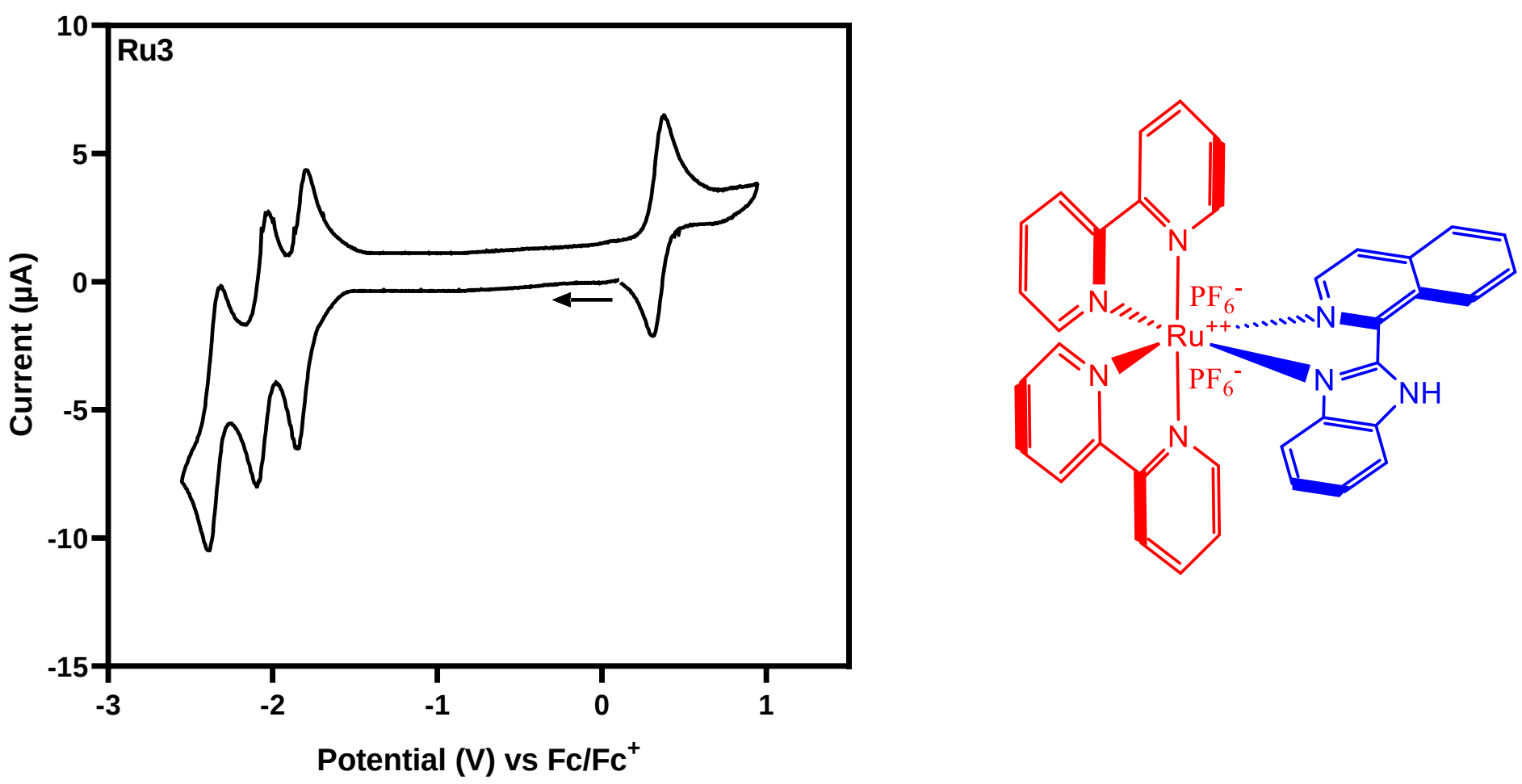

Figure S3. Cyclic voltammogram of **Ru3** (2 mM) in 0.1 M Bu4NPF6 ACN electrolyte, scan rate 0.3 V/s.

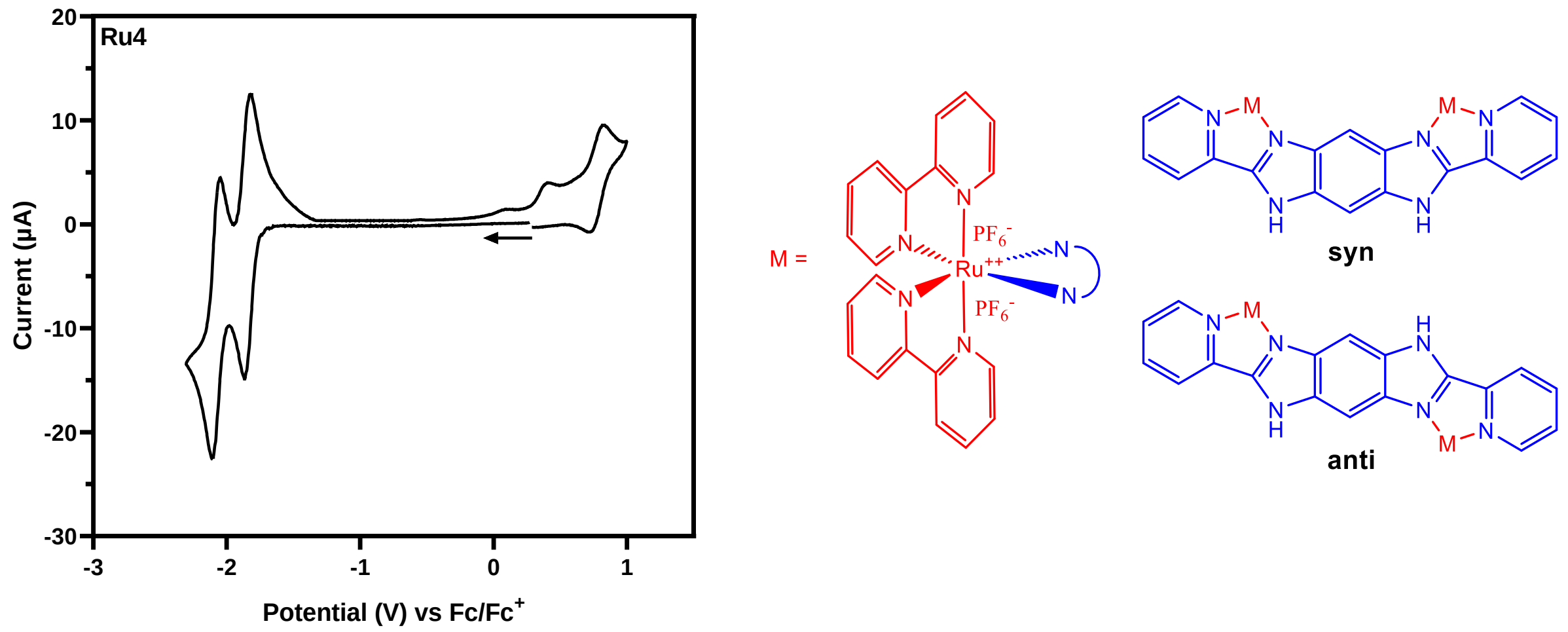

Figure S4. Cyclic voltammogram of **Ru4** (1 mM) in 0.1 M Bu4NPF6 ACN electrolyte, scan rate 0.3 V/s.

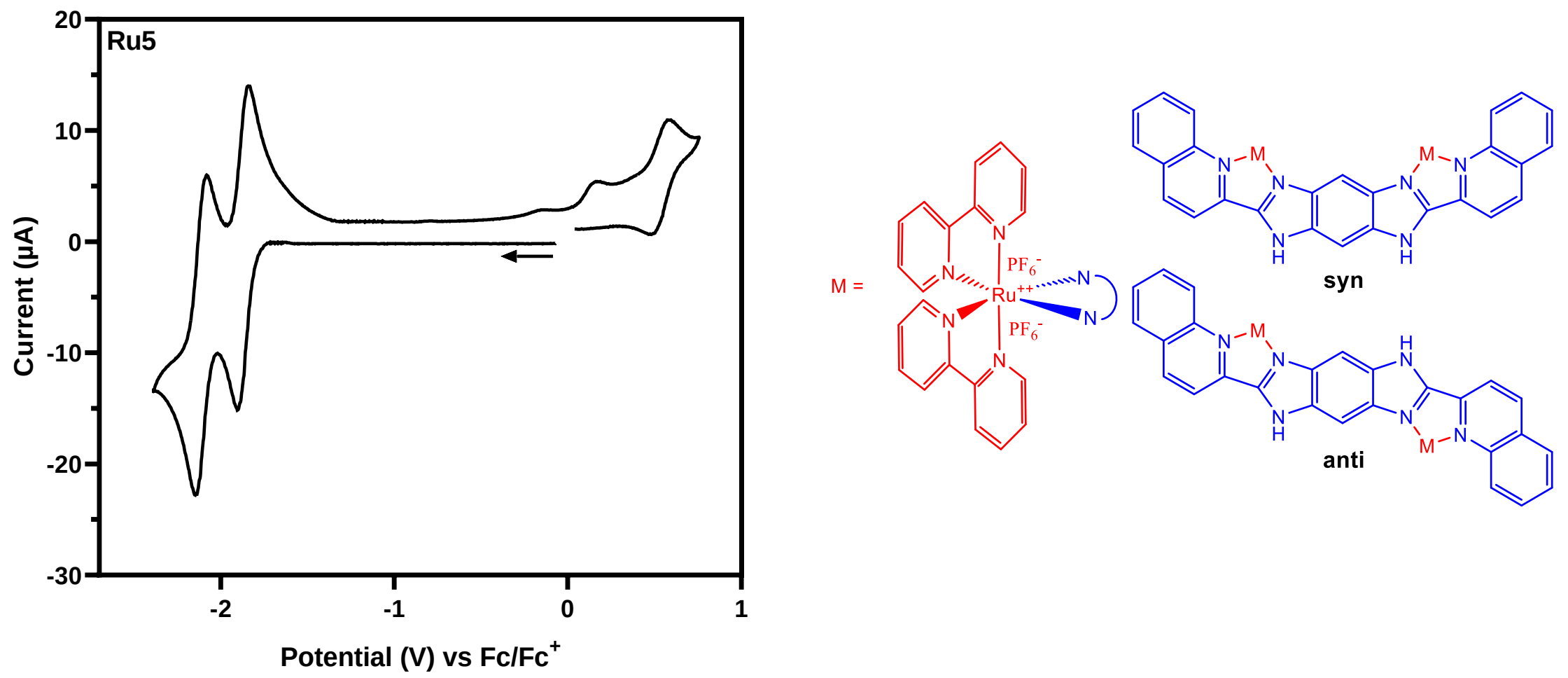

Figure S5. Cyclic voltammogram of **Ru5** (2 mM) in 0.1 M Bu4NPF6 ACN electrolyte, scan rate 0.3 V/s.

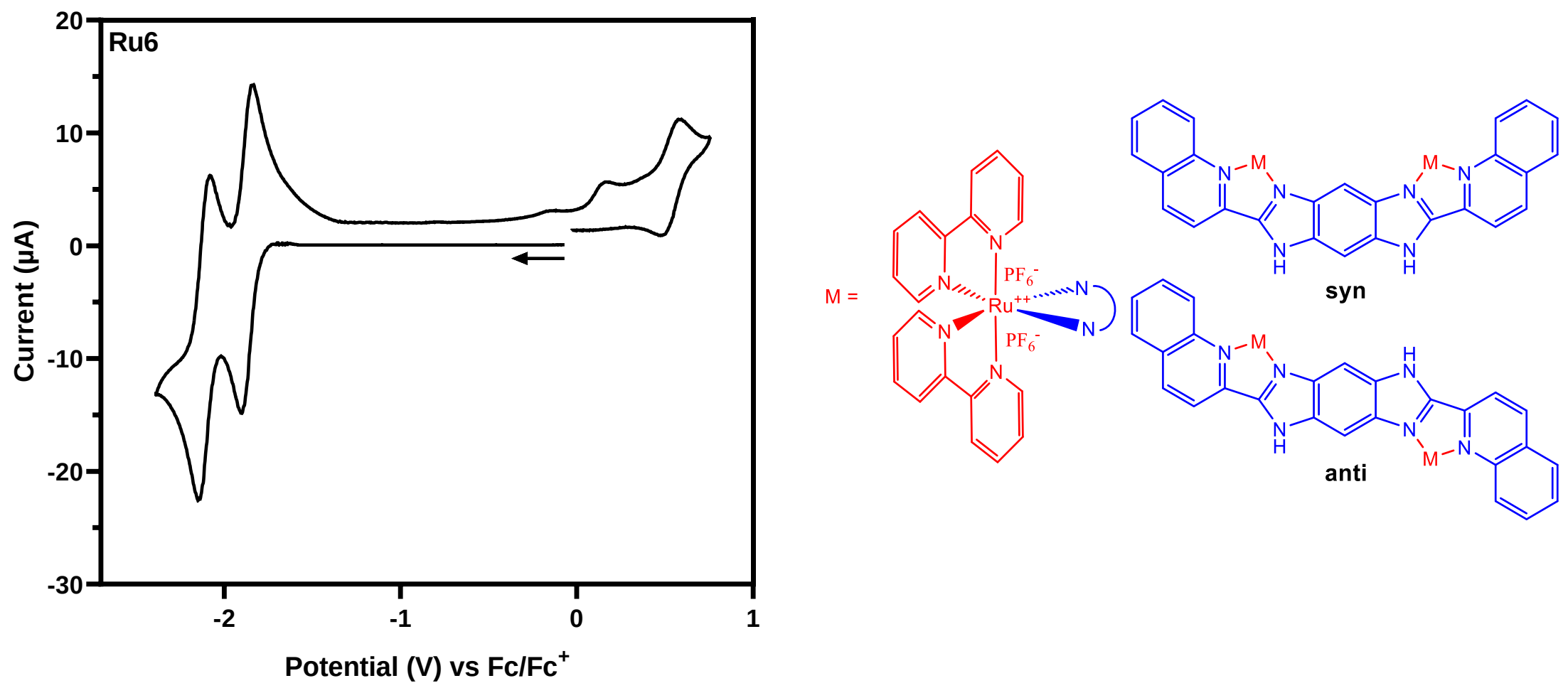

Figure S6. Cyclic voltammogram of **Ru6** (2 mM) in 0.1 M Bu4NPF6 ACN electrolyte, scan rate 0.3 V/s.

**Complexes**	**Reduction $E_{1/2}$ (eV)**			**E^*_{red} (V)**	**Oxidation $E_{1/2}$ (eV)**	**E^*_{ox} (V)**	**E_{00} (eV)**
	**Red3**	**Red2**	**Red1**				
**Ru1**		-2.07	-1.83	0.45	0.36	-1.92	2.28
**Ru2**	-2.34	-2.04	-1.81	0.19	0.38	-1.62	2.00
**Ru3**	-2.34	-2.05	-1.81	0.51	0.36	-1.96	2.32
**Ru4**		-2.02	-1.79	0.35	0.83	-1.31	2.14
**Ru5**		-2.11	-1.87	0.24	0.58	-1.53	2.11
**Ru6**	-2.40	-2.11	-1.87	0.15	0.55	-1.47	2.02

Table S1. All measurements were performed in 0.1 M nBu_4NPF_6/ACN. The potential is reported as $E_{1/2}$ versus Fc/Fc^+. The excited state redox potential (E*) of complexes was calculated based on the Rehm-Weller theory.

Absorbance and fluorescence spectra

Ligands

Each spectrum was recorded with a concentration of ligands at [30 µM] for absorbance and [7.5 µM] for fluorescence. The fluorescence spectra were recorded according to the λ_{max} of the corresponding ligands.

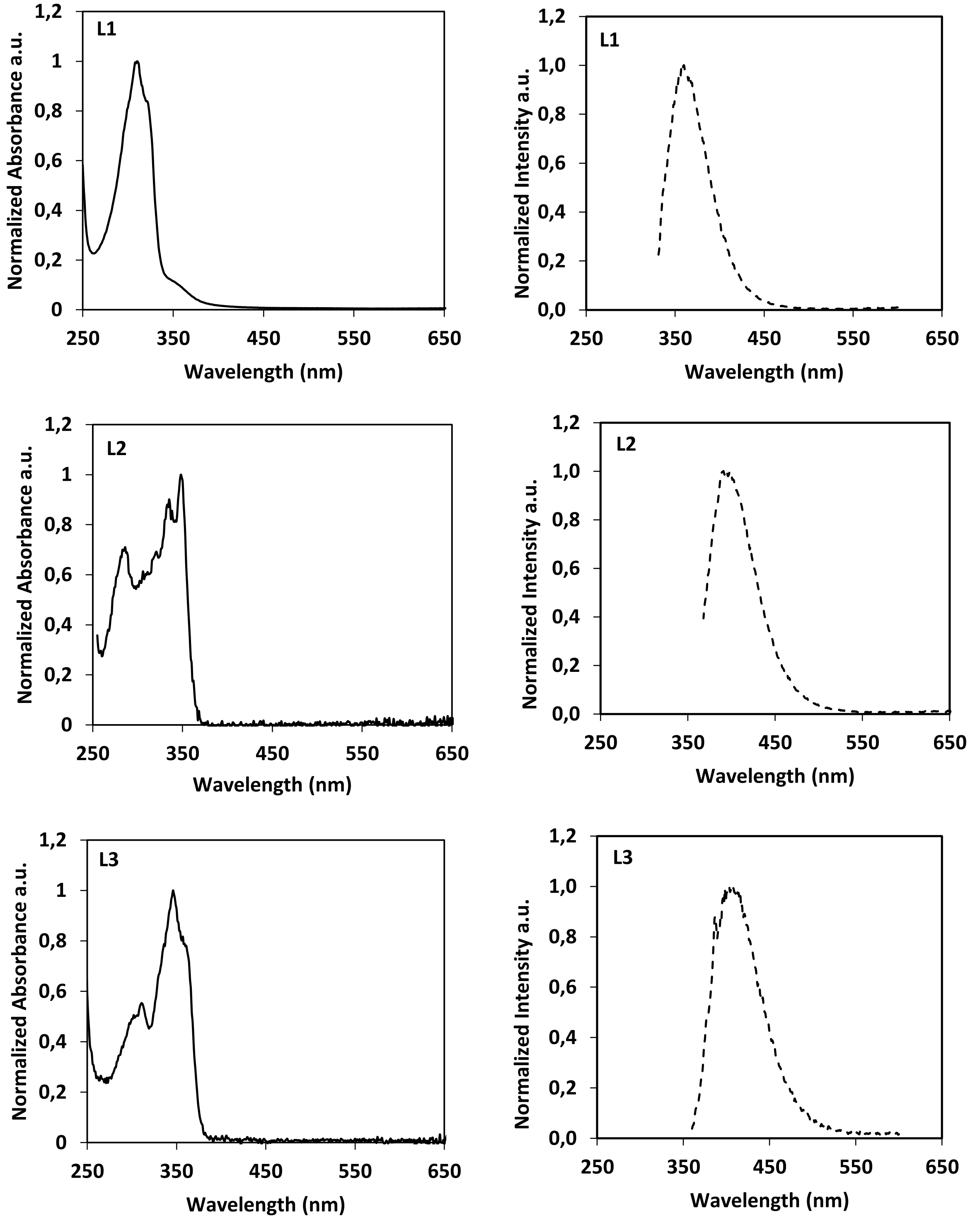

Figure S7. UV–vis spectra (absorbance, full line) at 30 µM in CH_3CN and emission spectra excited at 450 nm (intensity, dotted line) at 7.5 µM in CH_3CN of **L1–L3**.

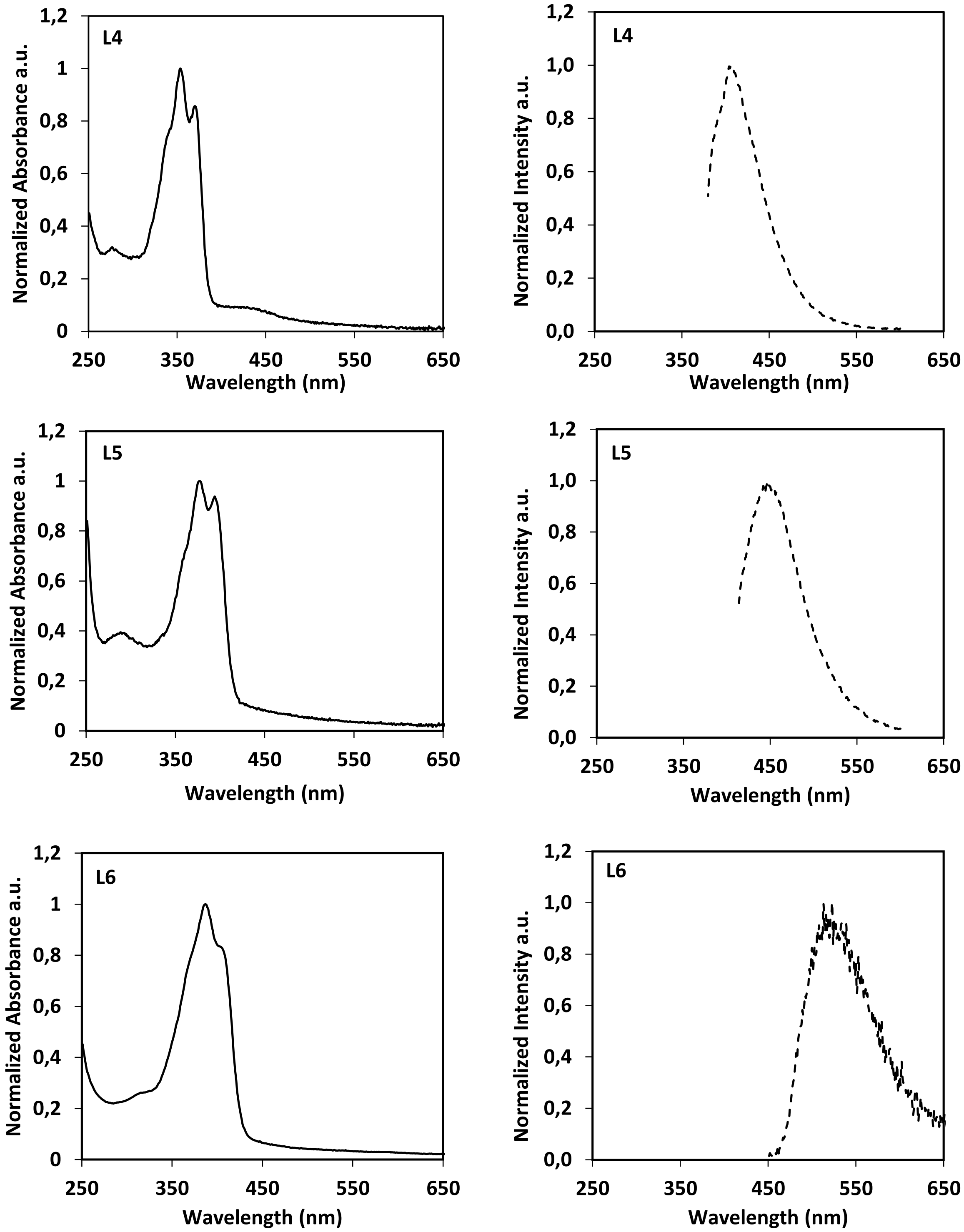

Figure S8. UV–vis spectra (absorbance, full line) at 30 µM in CH_3CN and emission spectra excited at 450 nm (intensity, dotted line) at 7.5 µM in CH_3CN of **L3–L6**.

Ruthenium complexes

Each spectrum was recorded with a concentration of ligands at [20 µM] in acetonitrile for the absorbance and the fluorescence. The fluorescence spectra were recorded at 450 nm.

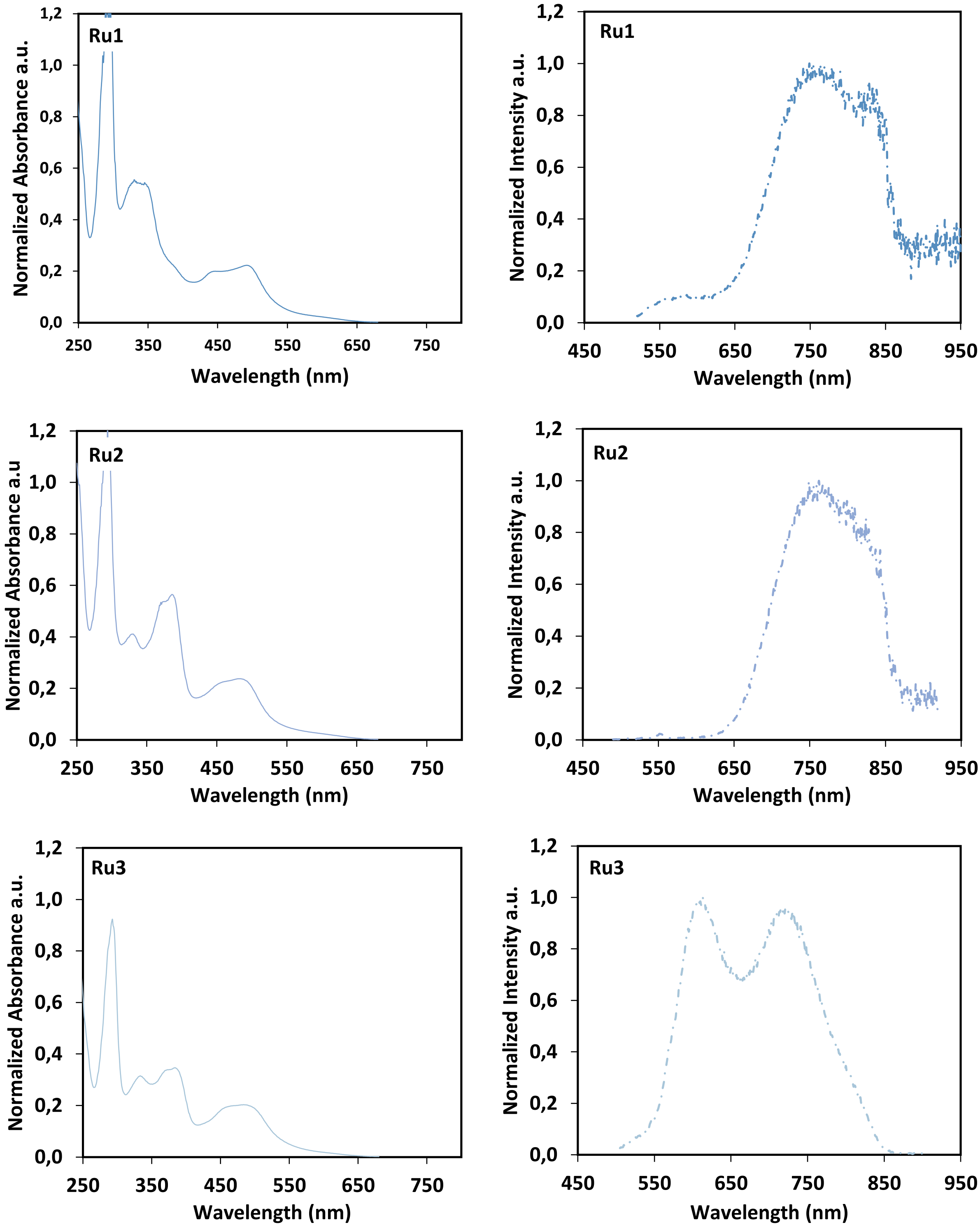

Figure S9. UV–vis spectra (absorbance, full line) and emission spectra excited at 450 nm (intensity, dotted line) of **Ru1–Ru3** at 20 µM in CH_3CN.

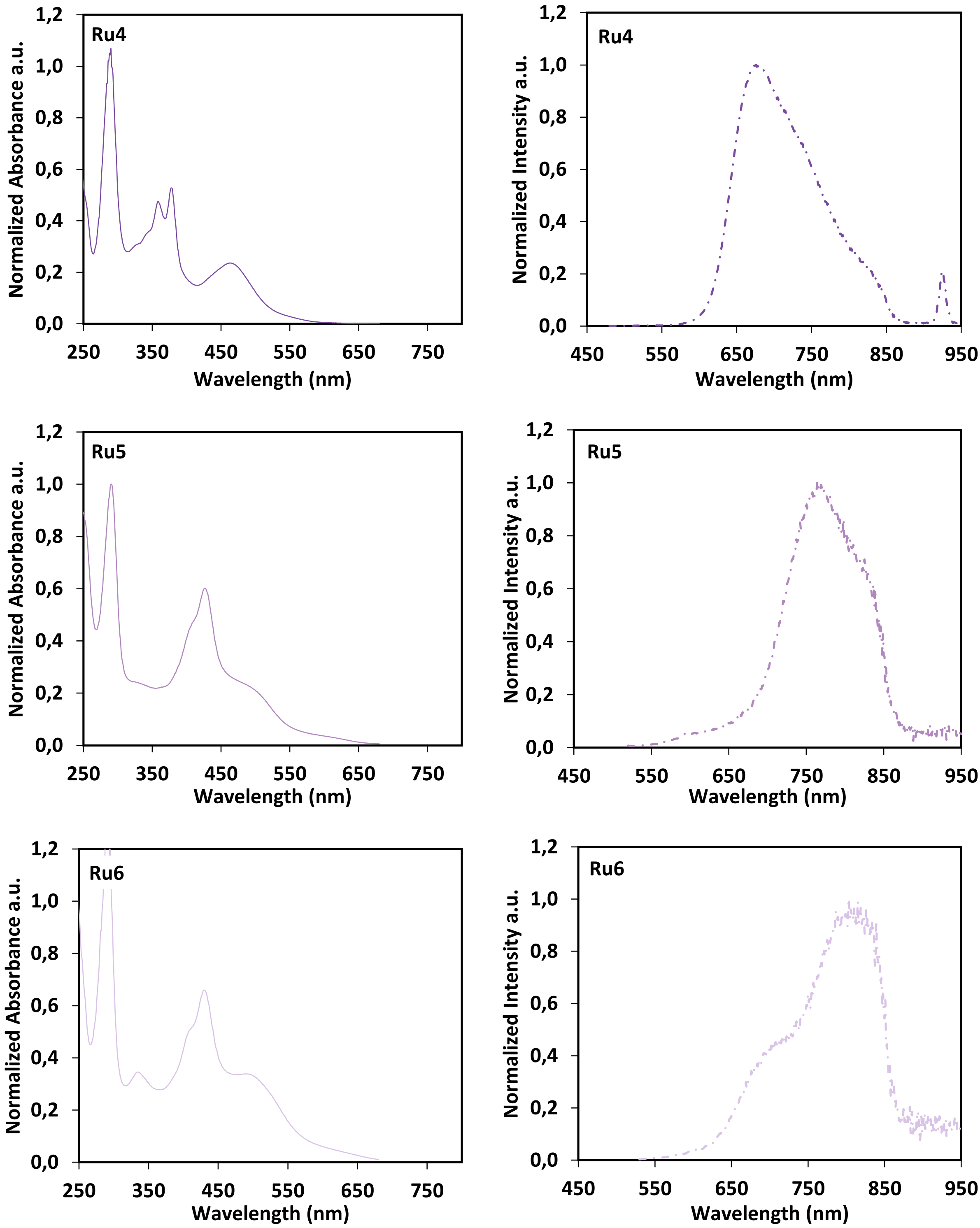

Figure S10. UV–vis spectra (absorbance, full line) and emission spectra excited at 450 nm (intensity, dotted line) of **Ru4-Ru6** at 20 µM in CH_3CN.

Compounds	Absorption λ_{max} (nm) (ε in $10^{-3}.M^{-1}.cm^{-1}$)	Emission λ_{max}[c] (nm)	Δ Stokes (nm)	Φ[d] (%)	E_{00} (eV)
L1	310 (60.0) [a]	360	50		
L2	285 (0.90), 335 (1.10), 348 (1.29) [a]	391	43		
L3	312 (1.08), 346 (1.85) [a]	403	57		
L4	279 (1.57), 354 (4.97) [a], 367 (4.27)	406	52		
L5	292 (2.00), 377 (5.03) [a], 393 (4.73)	462	85		
L6	390 (26.53) [a], 406 (21.90)	517	127		
Ru1	293 (67.34), 340 (31.20), 389 (9.34), 446 (11.48), 491 (12.60) [b]	567, 751	121; 260[b]	0.95	2.36
Ru2	295 (63.14), 332 (23.36), 368 (30.04), 388 (32.26), 457 (12.84), 483 (13.64) [b]	762	279[b]	0.55	2.00
Ru3	291 (51.74), 334 (18.10), 370 (19.34), 390 (19.00), 462 (11.30), 484 (11.68) [b]	607, 729	145, 245[b]	0.22	2.32
Ru4	287 (58.38), 306 (19.16), 379 (30.22), 462 (13.80) [b]	676	214[b]	0.57	2.14
Ru5	292 (40.88), 406 (18.82), 429 (24.80), 504 (8.30) [b]	618, 762	189, 258[b]	0.38	2.11
Ru6	289 (69.54), 408 (28.94), 432 (37.52), 508 (18.10) [b]	705, 802	273, 294[b]	0.17	2.02

Table S2. All measurements were performed at 30 µM for **L1-6** and **Ru1-6** in CH_3CN at 293 K. [a] values corresponding at λ_{max} of CT. [b] values corresponding at λ_{max} of MLCT. [c] values with λ excitation = 450 nm. [d] Quantum yield was calculated according to the equation, using $[Ru(bpy)_3]^{2+}$ (Φ = 1.8 %) as the standard [5].

[5] Rillema, D. P.; Taghdiri, D. G.; Jones, D. S.; Keller, C. D.; Worl, L. A.; Meyer, T. J.; Levy, H. A. *Inorg. Chem*. **1987**, 26, 578-585.

Study of the photostability

To study the photostability of ligands in acetonitrile, UV–visible absorption spectra were measured on a Molecular Device SpectraMax M5e in a 96-well plate at 50 µM. First, a UV analysis was performed with the plate reader at t0. Then, the plate was irradiated with Lumidox II LED Arrays (white light) for 48 h with UV analyses at different irradiation times (30 min, 1 h, 2 h and 20 h).

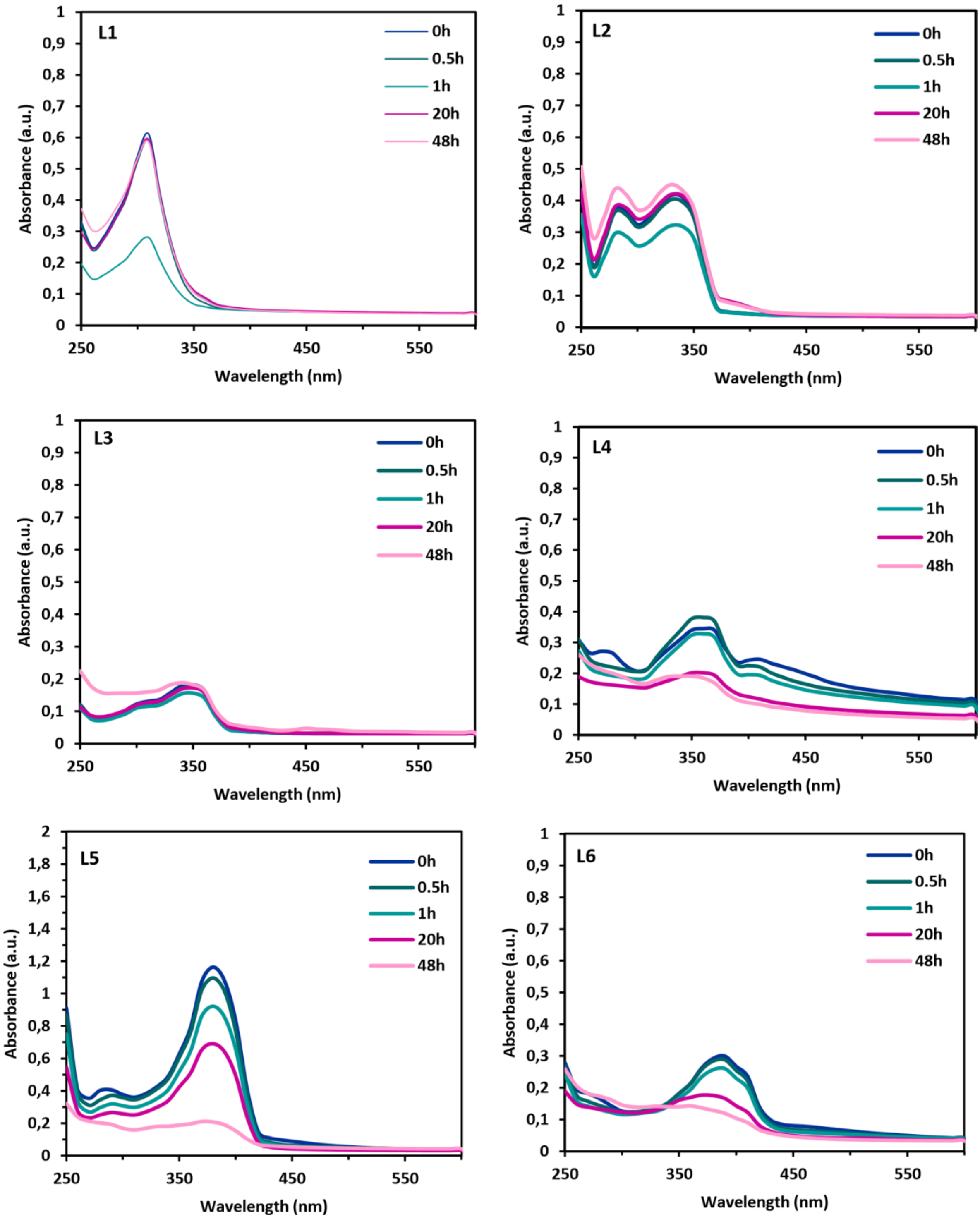

Figure S11. UV–vis spectra of **L1–L6** at 50 µM in CH_3CN after white light LED irradiation at different times.

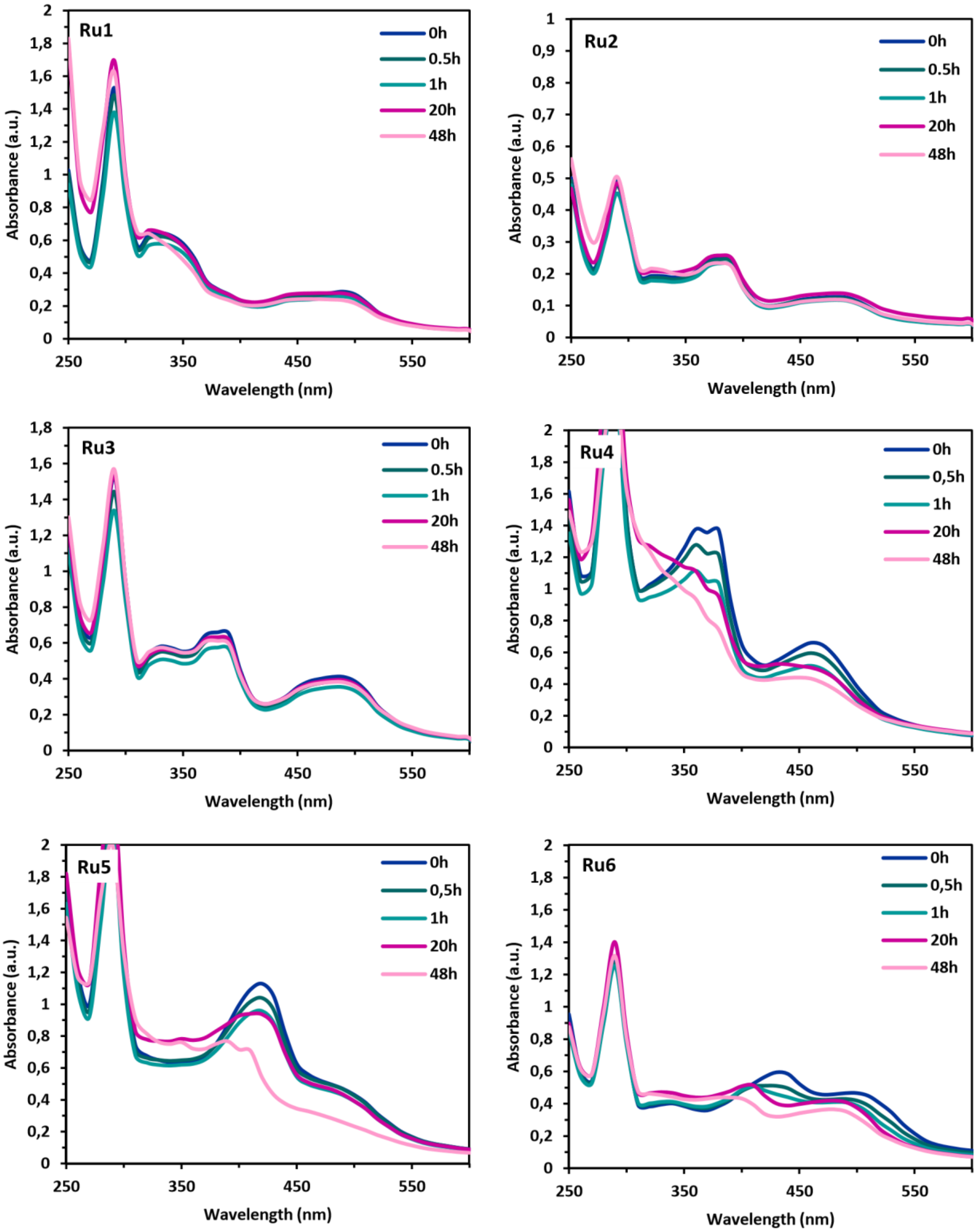

Figure S12. UV–vis spectra of **Ru1–Ru6** at 50 µM in CH_3CN after white light irradiation at different times.

Study of the ROS generation

The singlet oxygen generation

To detect singlet oxygen production by ruthenium complexes, 1,5-dihydroxynaphthalene (DHN) was used. 2.7 mL of DHN (340 µM) and 93 µL of ruthenium complexes (1 mM, 10 mol% vs DHN), dissolved in a mixture of MeOH/DCM (1/9) solution that has been pre-saturated with oxygen for 10 min, were added to a quartz cuvette 1 cm containing 207 µL of a mixture MeOH/DCM (1/9) solution. The final concentrations of DHN and ruthenium complexes were 310 µM and 31 µM, respectively. The concentration of DHN is deliberately higher than that of ruthenium because the latter absorb in the same region as DHN. Then, the mixture was illuminated with a Kessil® PR160-456 nm LED at room temperature for 60 seconds with UV analyses every 5 seconds. The $^1O_2$ generation rate was determined from the increase in absorbance of juglone at 420 nm over time. To characterize the difference in the rate of $^1O_2$ produced by different samples, the ratios $(A - A_0)/A_0$ of absorbance of juglone in presence of ruthenium A and the initial absorbance of juglone A_0 at 420 nm at different irradiation times, were calculated and plotted as the ordinate for the irradiation time.

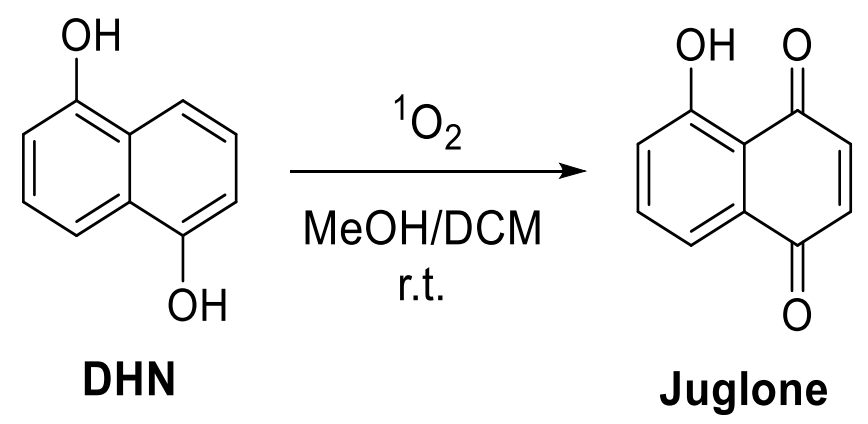

Scheme S3. Synthetic scheme of oxidation of DHN in juglone.

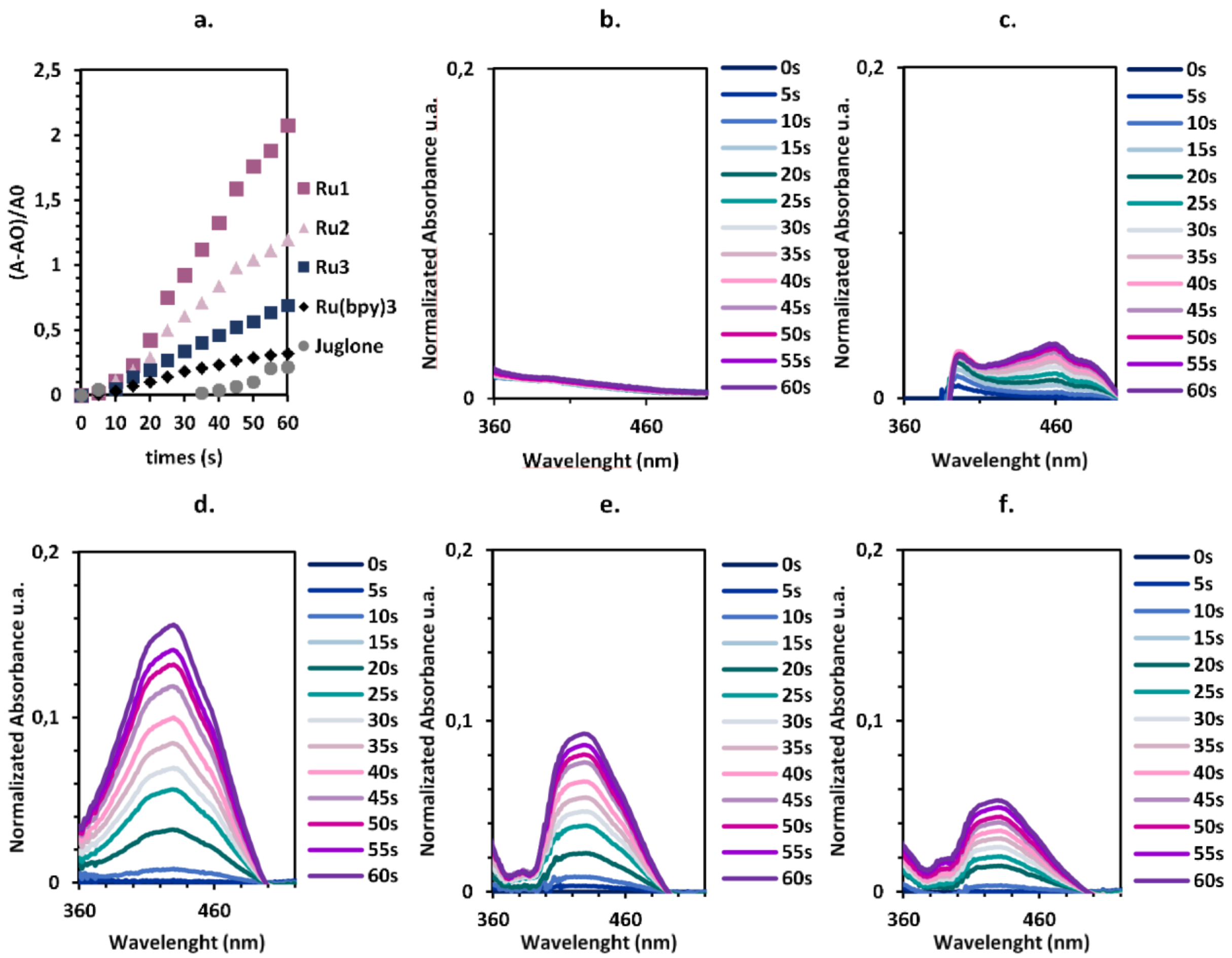

Figure S13. (a) Graphical representation of singlet oxygen production of **Ru1–Ru3** correlated with the increase of the intensity of juglone absorption band (420 nm) at different times (0 to 60 seconds). UV–vis spectra of (b) DHN, (c) $[Ru(bpy)_3](PF_6)_2$, (d) **Ru1**, (e) **Ru2** and (f) **Ru3**. **Ru** at 31 µM and DHN at 310 µM in a mixture of MeOH/DCM (1/9, v/v) after irradiation at 456 nm at different times (0 to 60 seconds).

The ROS generation by a SET mechanism

To detect ROS production via a SET mechanism by ruthenium, 2',7'-Dichlorodihydrofluorescein diacetate (DCFH) was used. 15 µL of DCFH (1 mM in DMSO) and 15 µL of ruthenium complexes (1 mM in DMSO) were added to a quartz cuvette 1 cm containing 2.97 mL of H_2O. The final concentrations of DCFH and ruthenium were 10 µM in a mixture of DMSO/H_2O (1/99) for each compound. DCFH was used as the total ROS indicator. Then, the mixture was illuminated with a Kessil® PR160-456 nm LED (under 40 mW·cm^{-2}) and at room temperature for 60 seconds with fluorescence analyses every 5 seconds. The fluorescence spectra of conversion of DCFH to DCF (excited at 488 nm) were continuously monitored during the predefined irradiation time. The ROS generation rate was determined from the increase in fluorescence of DCFH at 520 nm over time. To characterize the difference in the rate of ROS produced by different samples, the ratios I/I_0 of fluorescence of DCF in presence of ruthenium I and the initial fluorescence of DCF I_0 at 520 nm at different irradiation times, were calculated and plotted as the ordinate for the irradiation time.

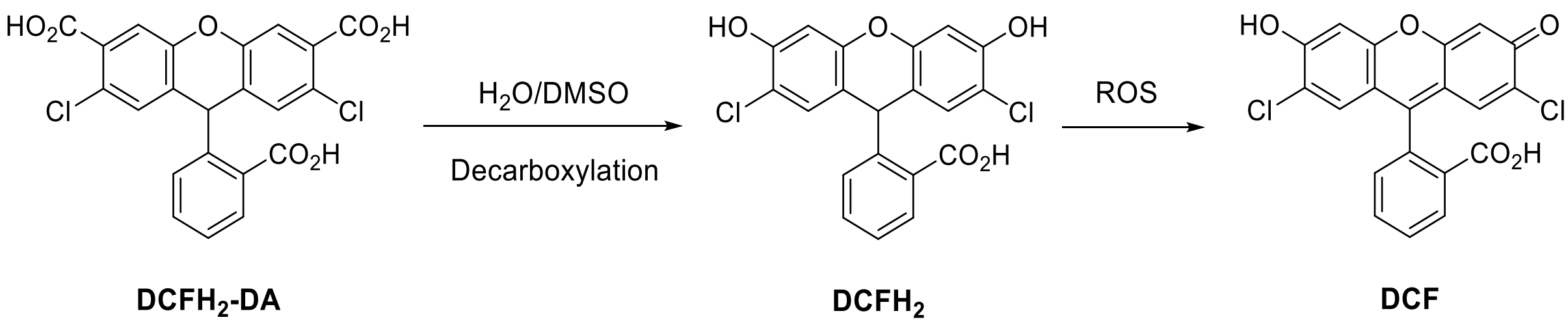

Scheme S4. Synthetic scheme of oxidation of DCFH-DA in DCF.

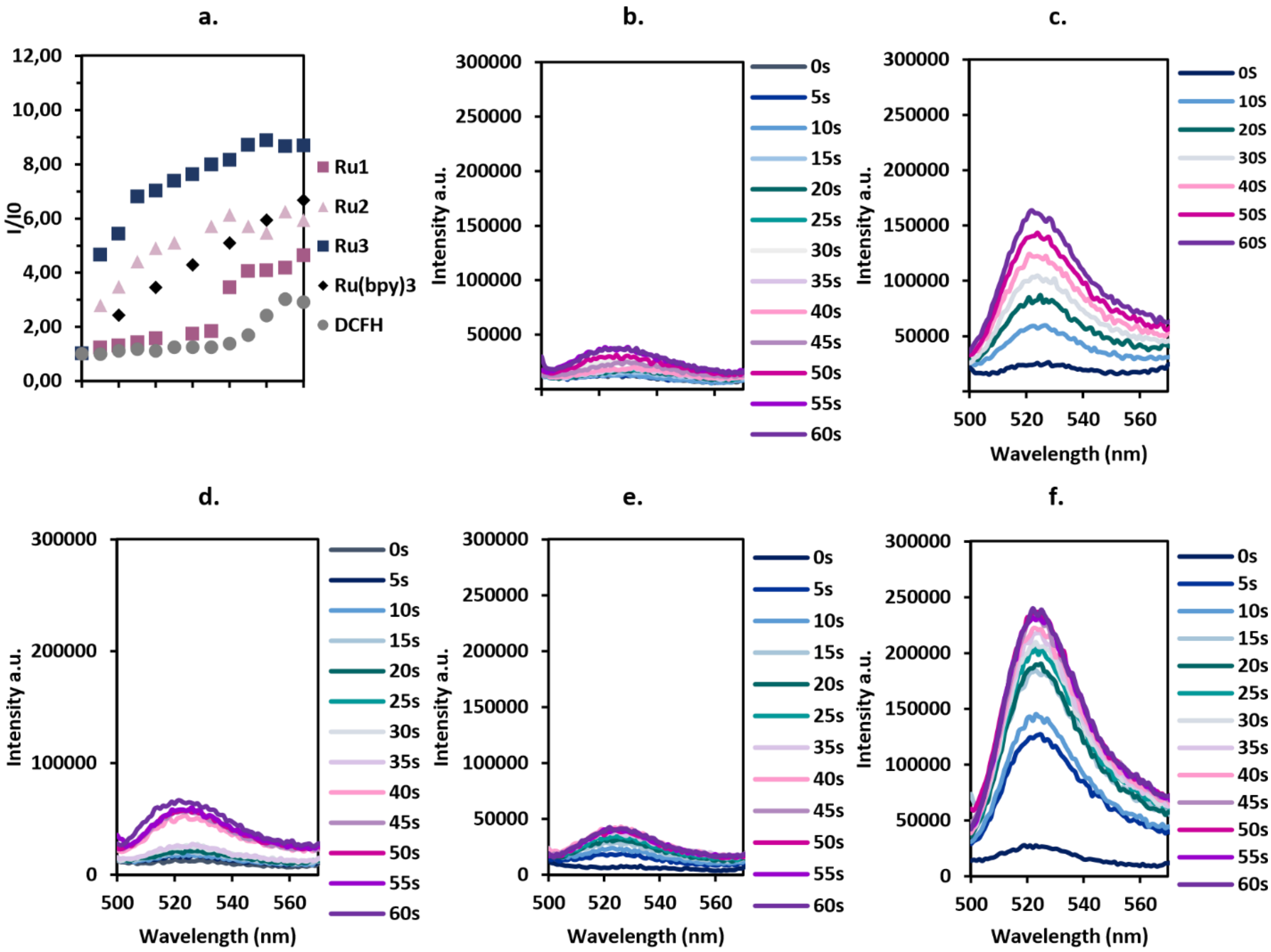

Figure S14. (a) Graphical representation of ROS production of **Ru1–Ru3** correlated with the increase of the intensity of DCF emission band at 520 nm at different times (0 to 60 seconds). Emission spectra excited at 488 nm of (b) DCFH, and DCFH in presence of (c) $[Ru(bpy)_3](PF_6)_2$, (d) **Ru1**, (e) **Ru2** and (f) **Ru3**. **Ru1–Ru3** and DCFH at 10 µM in a mixture DMSO/H2O (1/99, v/v) after irradiation at 456 nm.

Model photo-reaction by SET mechanism

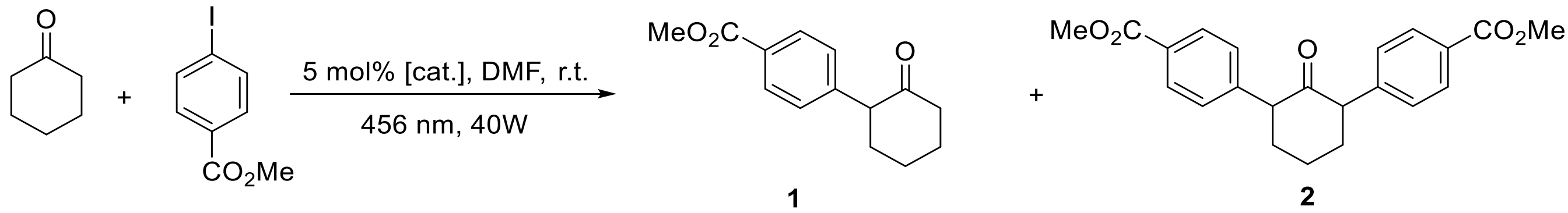

Scheme S5. Synthetic scheme of α-arylation of cyclohexanone.

Cyclohexanone (1 equiv, 0.1 M), methyl 4-iodobenzoate ester (1 equiv) and pyrrolidine (2 equiv) in acetonitrile were stirred in presence of catalysts (5 mol %, 0.005 mmol) under 456 nm irradiation for 17h30 at room temperature in a sealed vial. For each experiment, 3 vials were using (without photocatalyst, with photocatalyst **Ru** and without light). The following experiments were monitored by LC-MS analysis using an intern standard (IS = biphenyl at 1 mM).

Entry	Conditions	% Iodide SM[a]	% Monosubstituted Conversion (1)	% Disubstituted Conversion (2)
1	w/o catalyst	100	0	0
2	$[Ru(bpy)_3](PF_6)_2$	11	28	61
3	$[Ru(bpy)_3](PF_6)_2$, w/o light	100	0	0
4	Ru1	71	4	25
5	Ru1, w/o light	100	0	0
6	Ru2	76	0	24
7	Ru2, w/o light	100	0	0
8	Ru3	14	22	64
9	Ru3, w/o light	100	0	0

Table S3. Results of the α-arylation experiment realized at 0.1 M of starting material in 1 mL of acetonitrile. [a] The starting material is methyl 4-iodobenzoate ester. w/o corresponds to without.

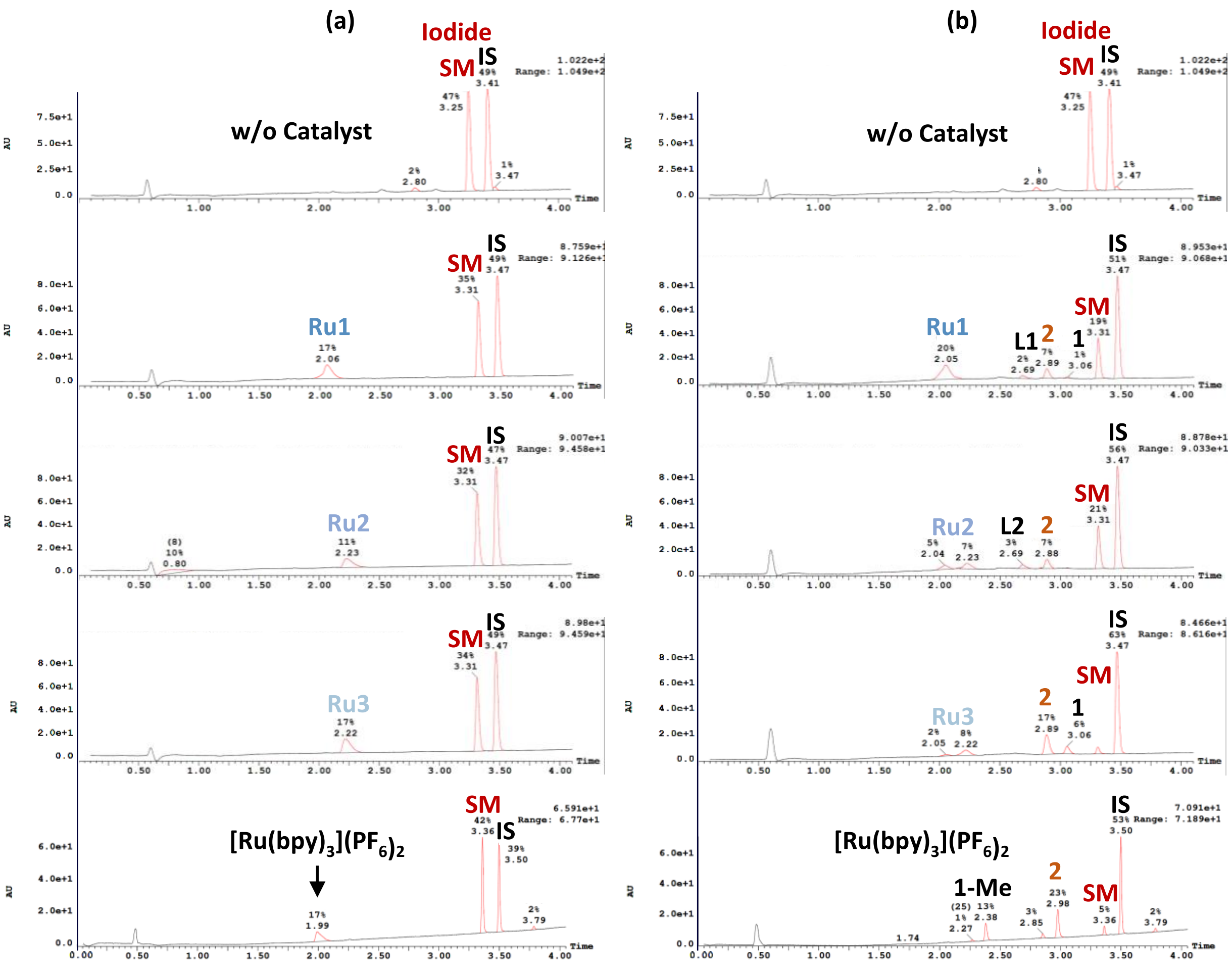

Figure S15. LC-MS spectra (a) after 17h30 of reaction at room temperature without light (w/o light) and (b) after 17h30 reaction at room temperature under irradiation at 456 nm. IS = biphenyl (3.5 min), **1** = monosubstituted (3.1 min), **1-Me** = carboxylate monosubstituted (2.4 min), **2** = disubstituted (2.9 min) and **SM** = Iodide start*ing* material (3.3 min).

Spectra

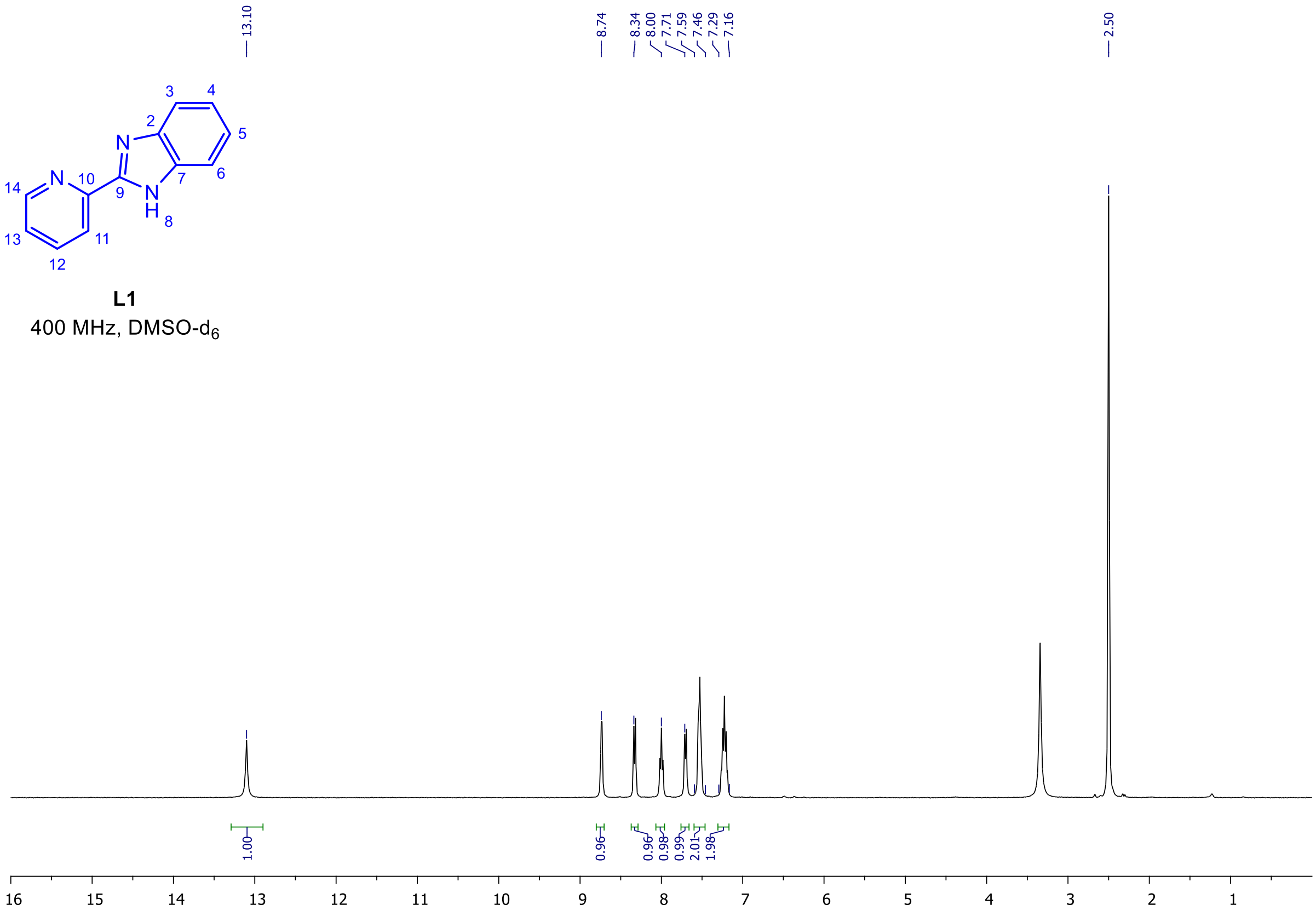
13.10
8.74
8.34
8.00
7.71
7.59
7.46
7.29
7.16
2.50
L1
400 MHz, DMSO-d6
1.00
0.96
0.96
0.98
0.99
2.01
1.98
16
15
14
13
12
11
10
9
8
7
6
5
4
3
2
1

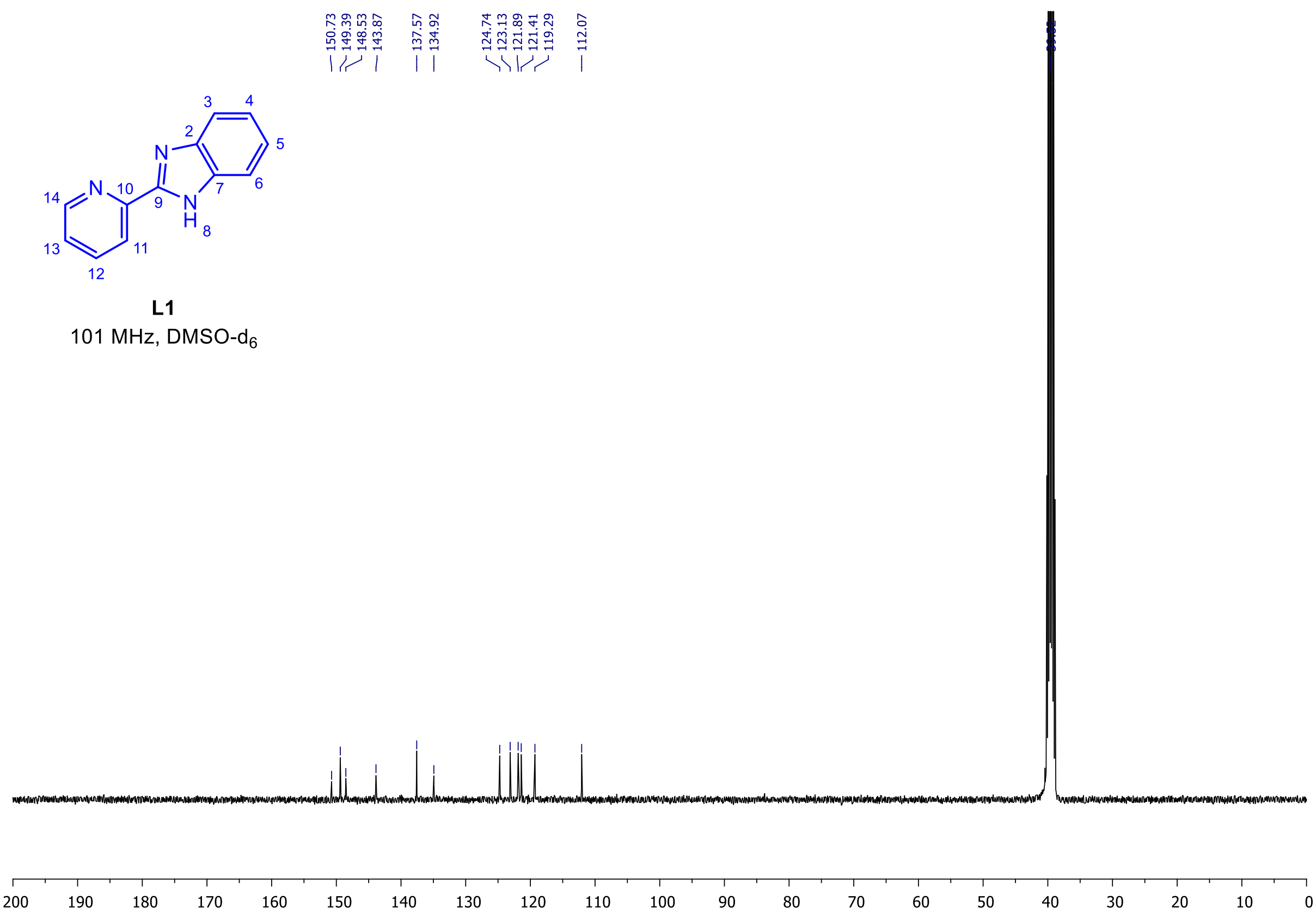
150.73
149.39
148.53
143.87
137.57
134.92
124.74
123.13
121.89
121.41
119.29
112.07
L1
101 MHz, DMSO-d6
200
190
180
170
160
150
140
130
120
110
100
90
80
70
60
50
40
30
20
10
0

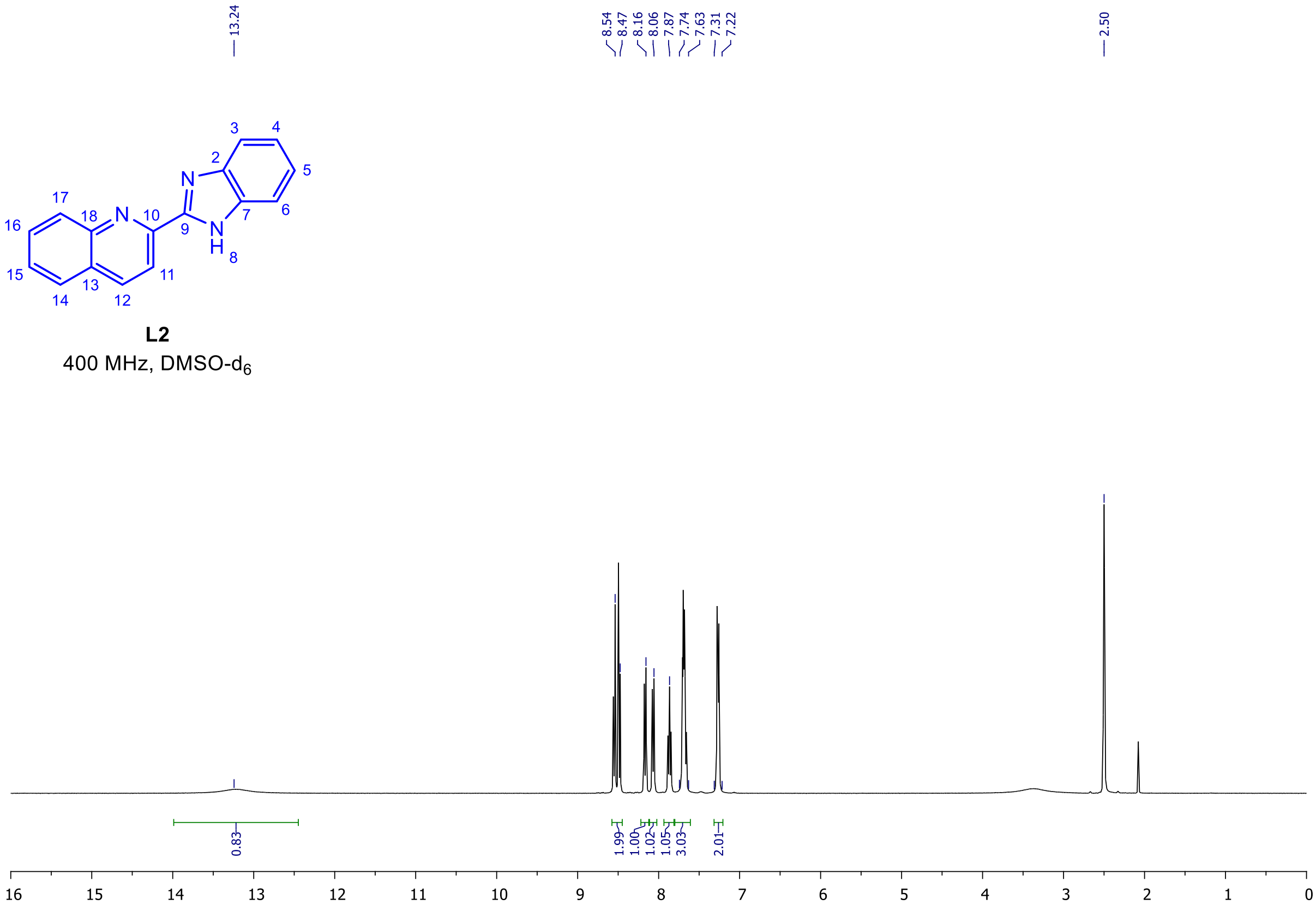

L2
400 MHz, DMSO-d_6

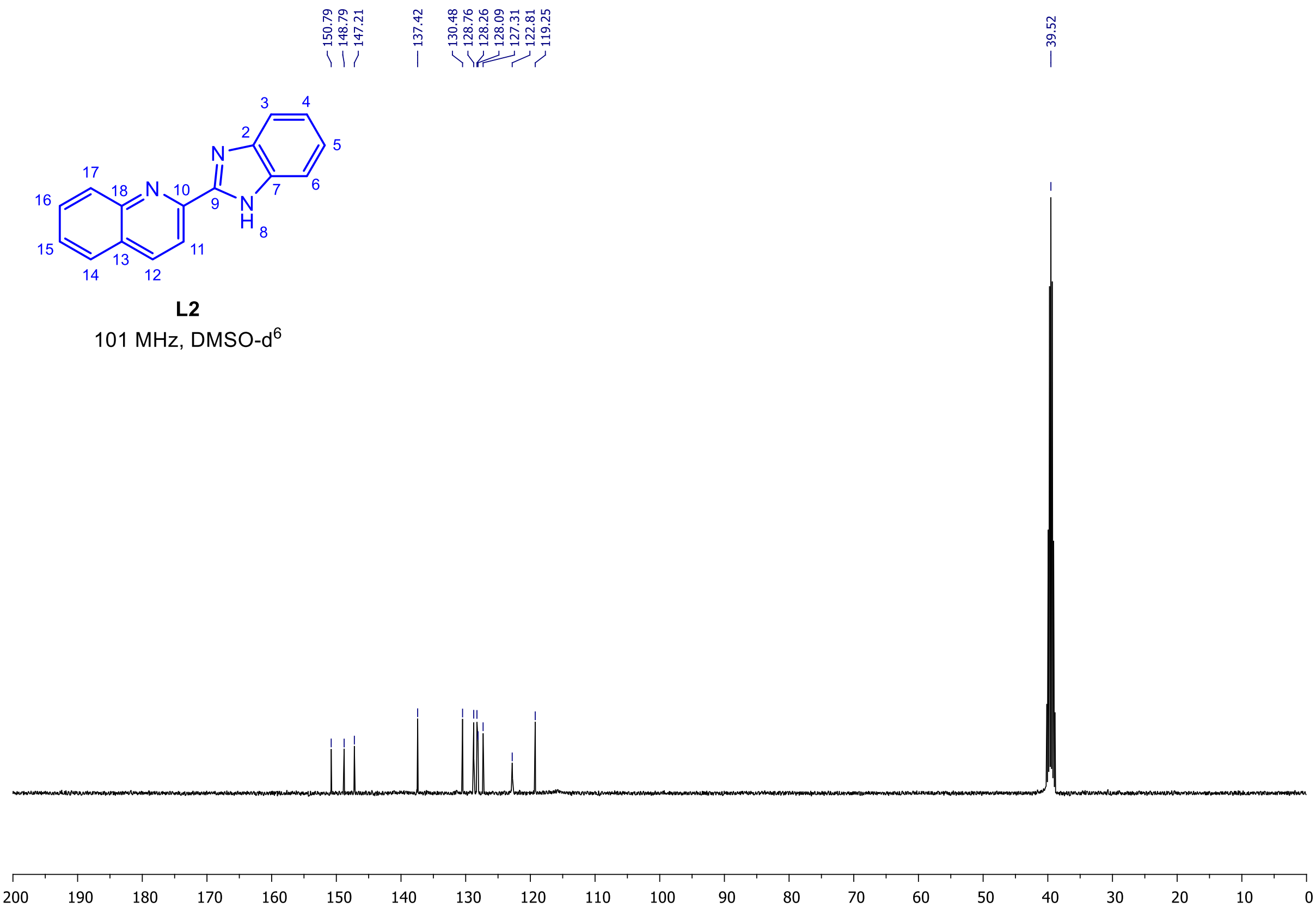

L2
101 MHz, DMSO-d^6

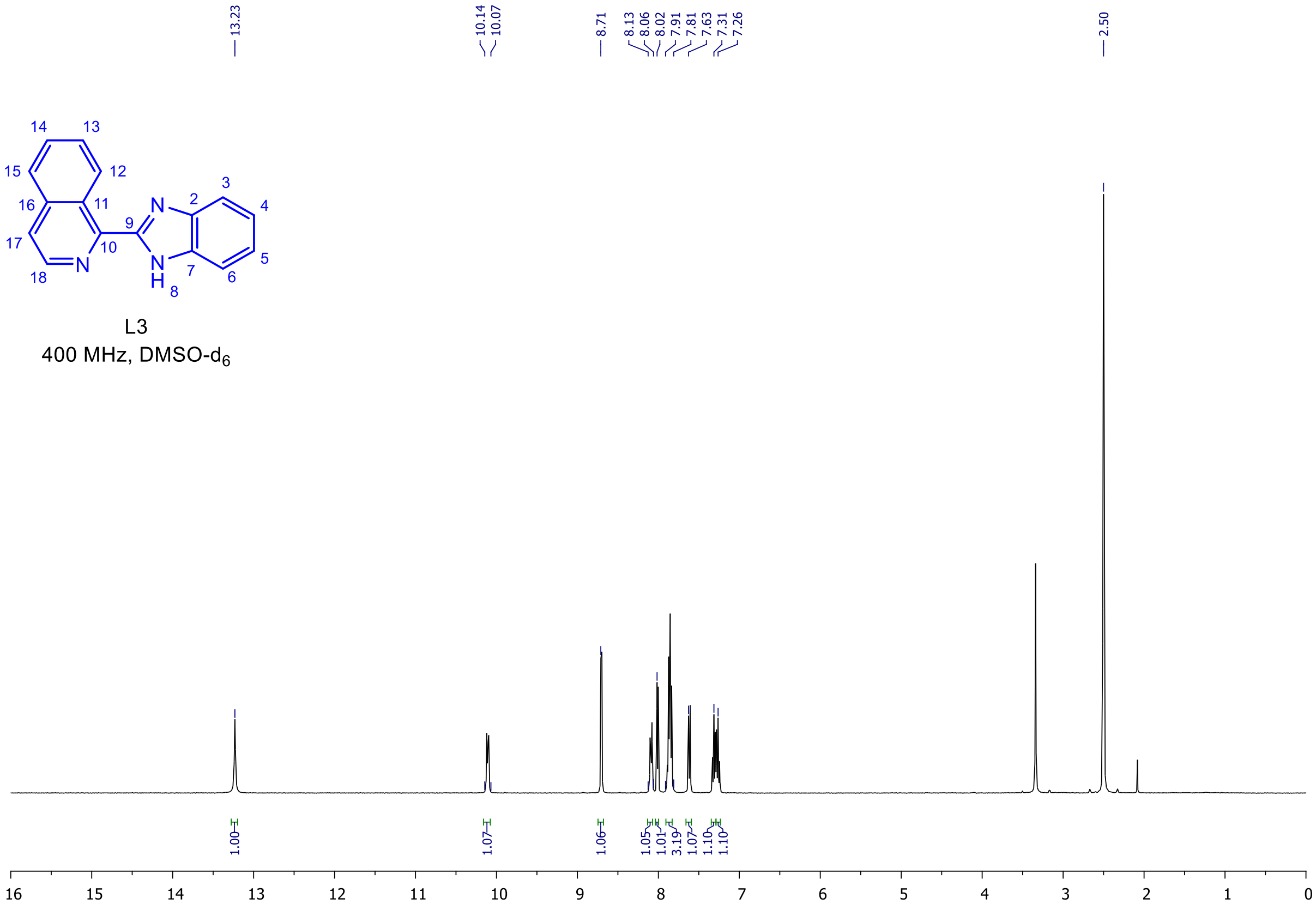
13.23
10.14
10.07
8.71
8.13
8.06
8.02
7.91
7.81
7.63
7.31
7.26
2.50
1.00
1.07
1.06
1.05
1.01
3.19
1.07
1.10
1.10
L3
400 MHz, DMSO-d6

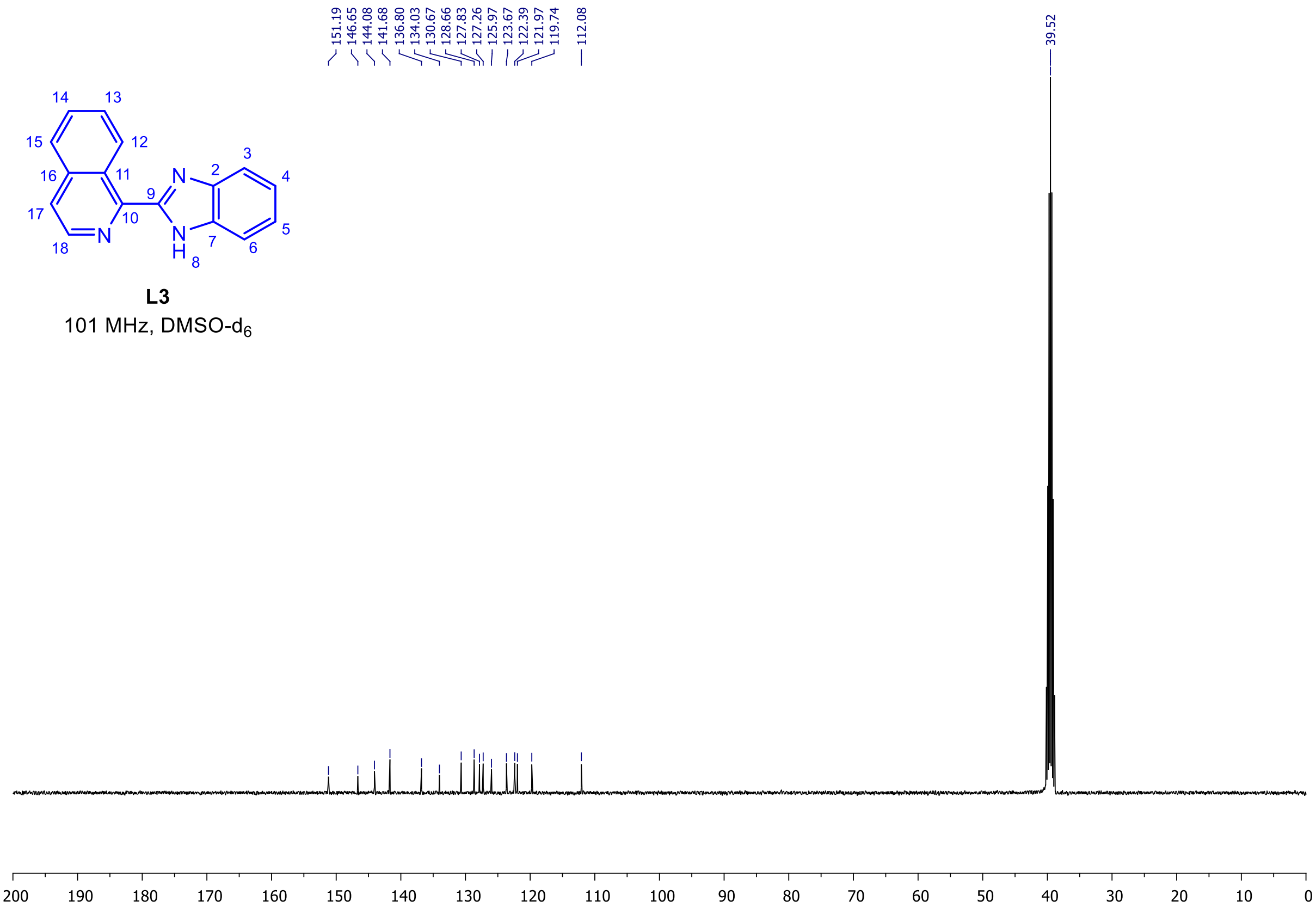
151.19
146.65
144.08
141.68
136.80
134.03
130.67
128.66
127.83
127.26
125.97
123.67
122.39
121.97
119.74
112.08
39.52
L3
101 MHz, DMSO-d6

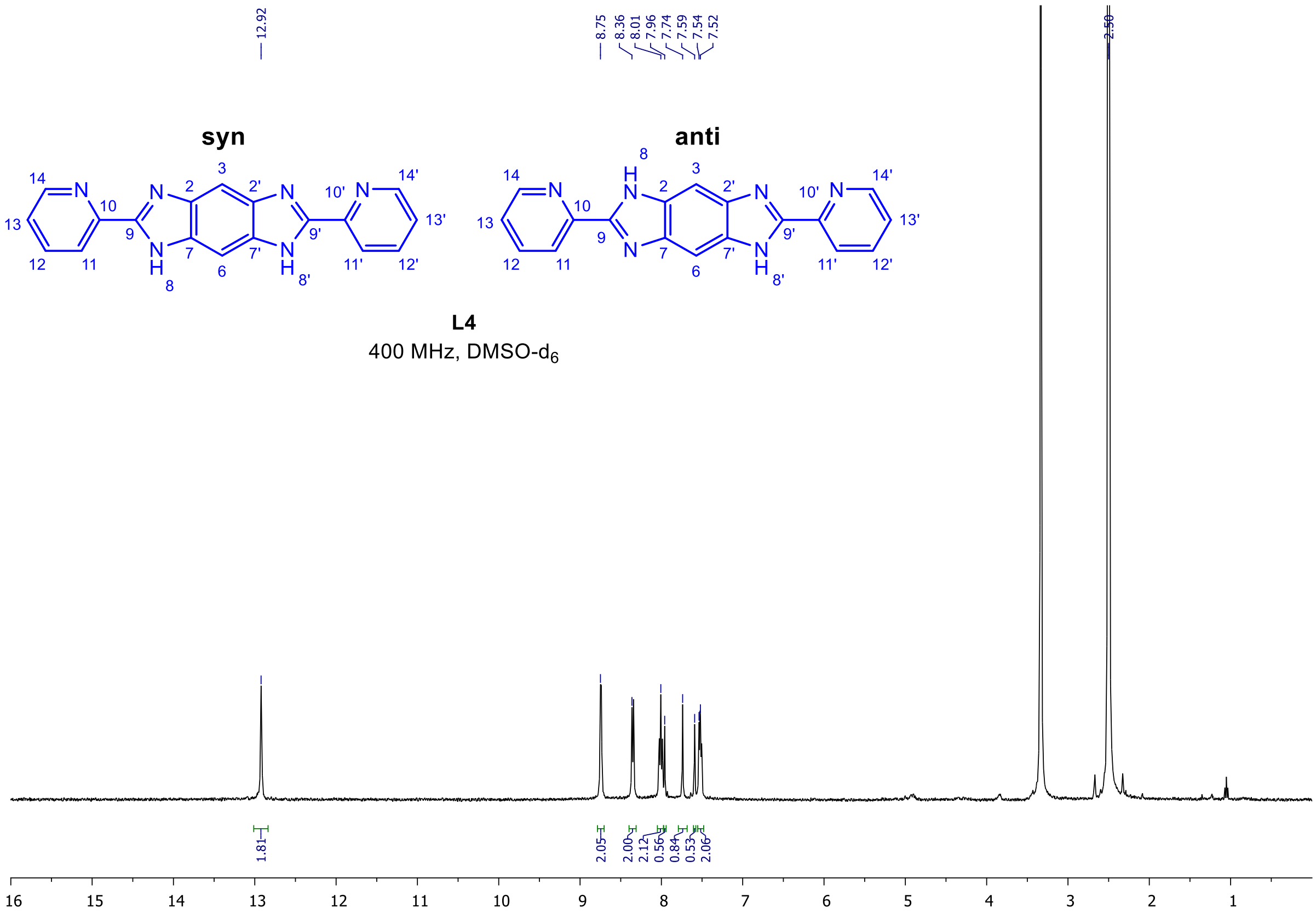
12.92
8.75
8.36
8.01
7.96
7.74
7.59
7.54
7.52
2.50
syn
anti
L4
400 MHz, DMSO-d6
1.81
2.05
2.00
2.12
0.56
0.84
0.53
2.06
16
15
14
13
12
11
10
9
8
7
6
5
4
3
2
1

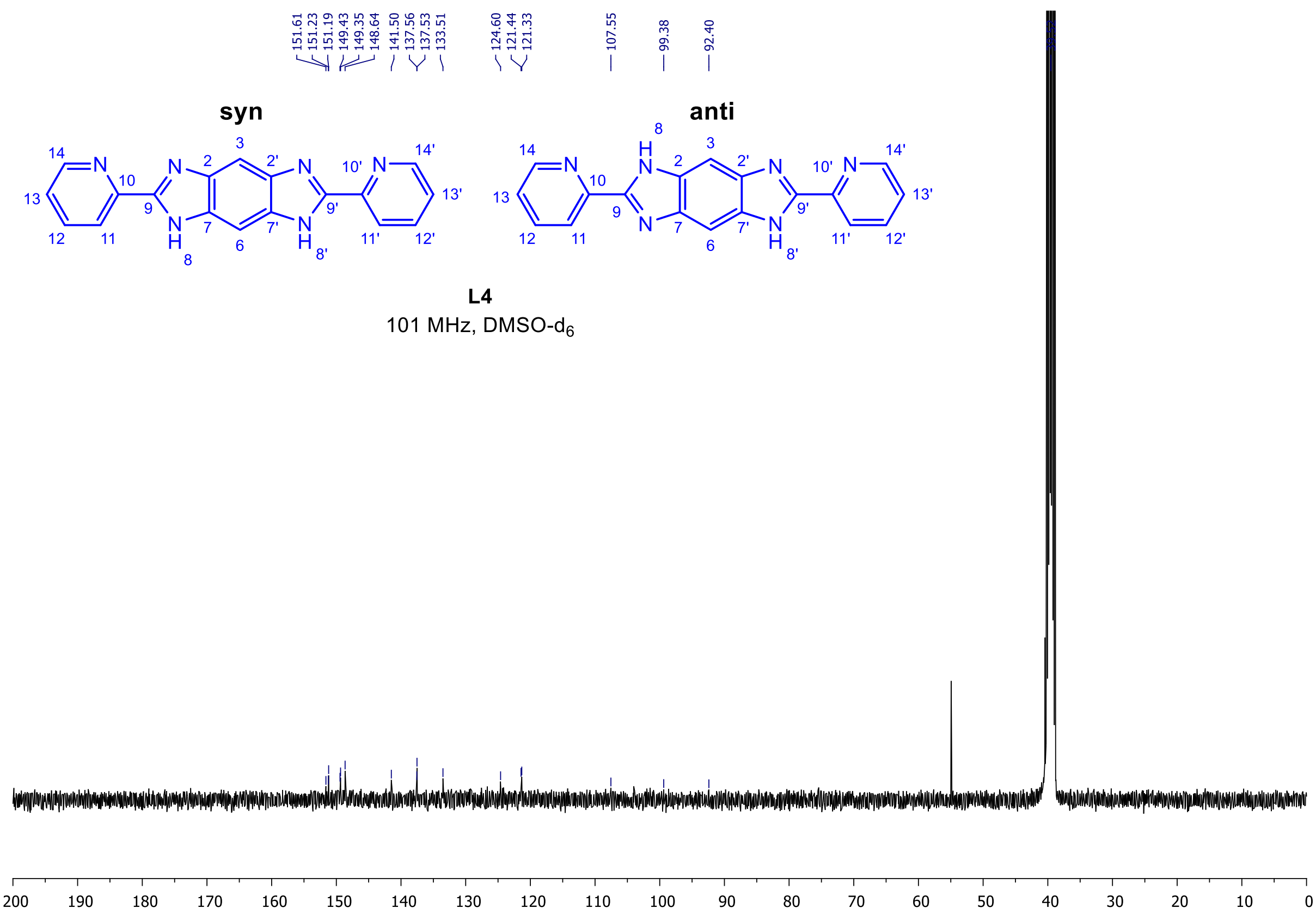
151.61
151.23
151.19
149.43
149.35
148.64
141.50
137.56
137.53
133.51
124.60
121.44
121.33
107.55
99.38
92.40
syn
anti
L4
101 MHz, DMSO-d6
200
190
180
170
160
150
140
130
120
110
100
90
80
70
60
50
40
30
20
10
0

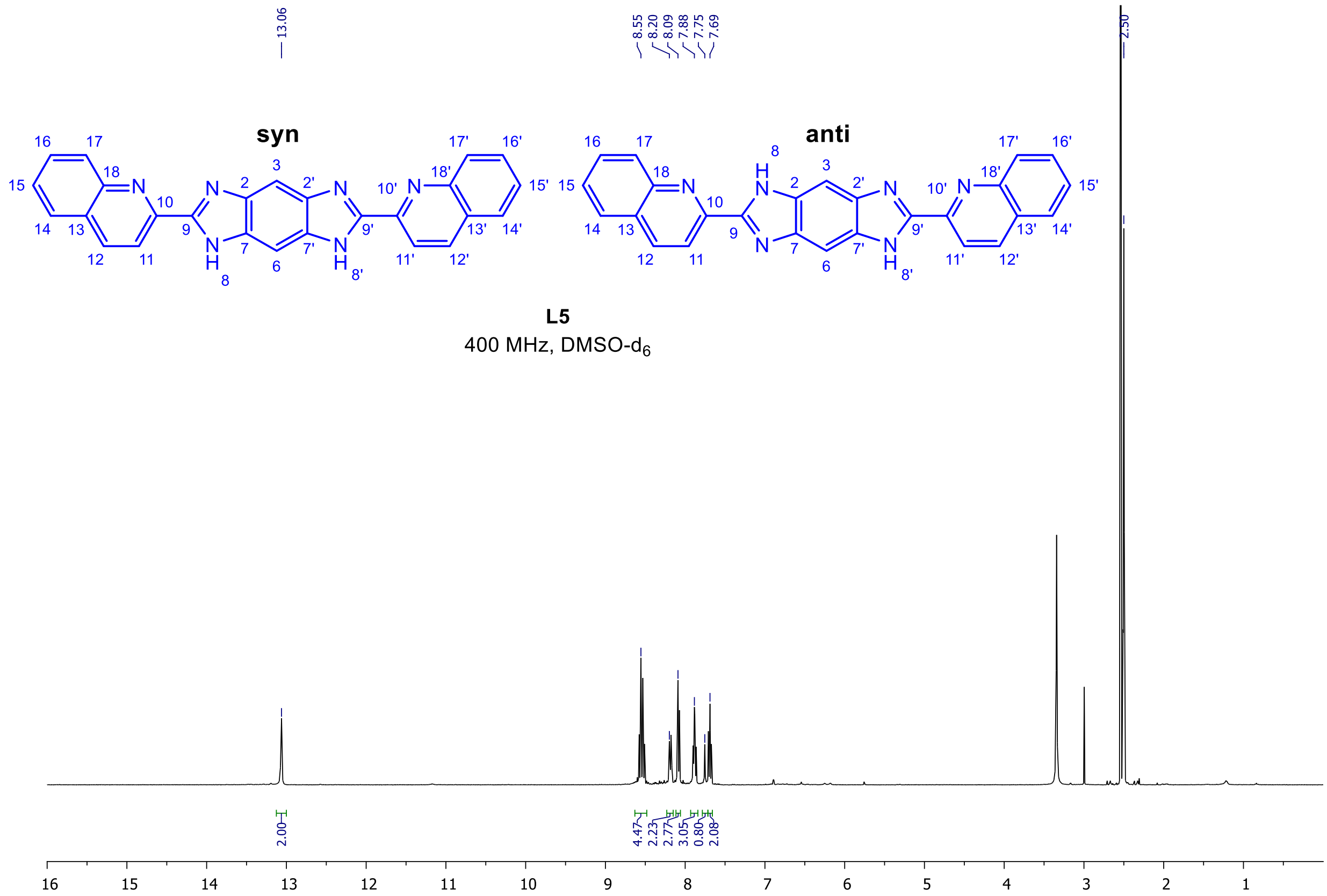
syn
anti
L5
400 MHz, DMSO-d$_6$
13.06
8.55
8.20
8.09
7.88
7.75
7.69
2.50
2.00
4.47
2.23
2.77
3.05
0.80
2.08
16 15 14 13 12 11 10 9 8 7 6 5 4 3 2 1

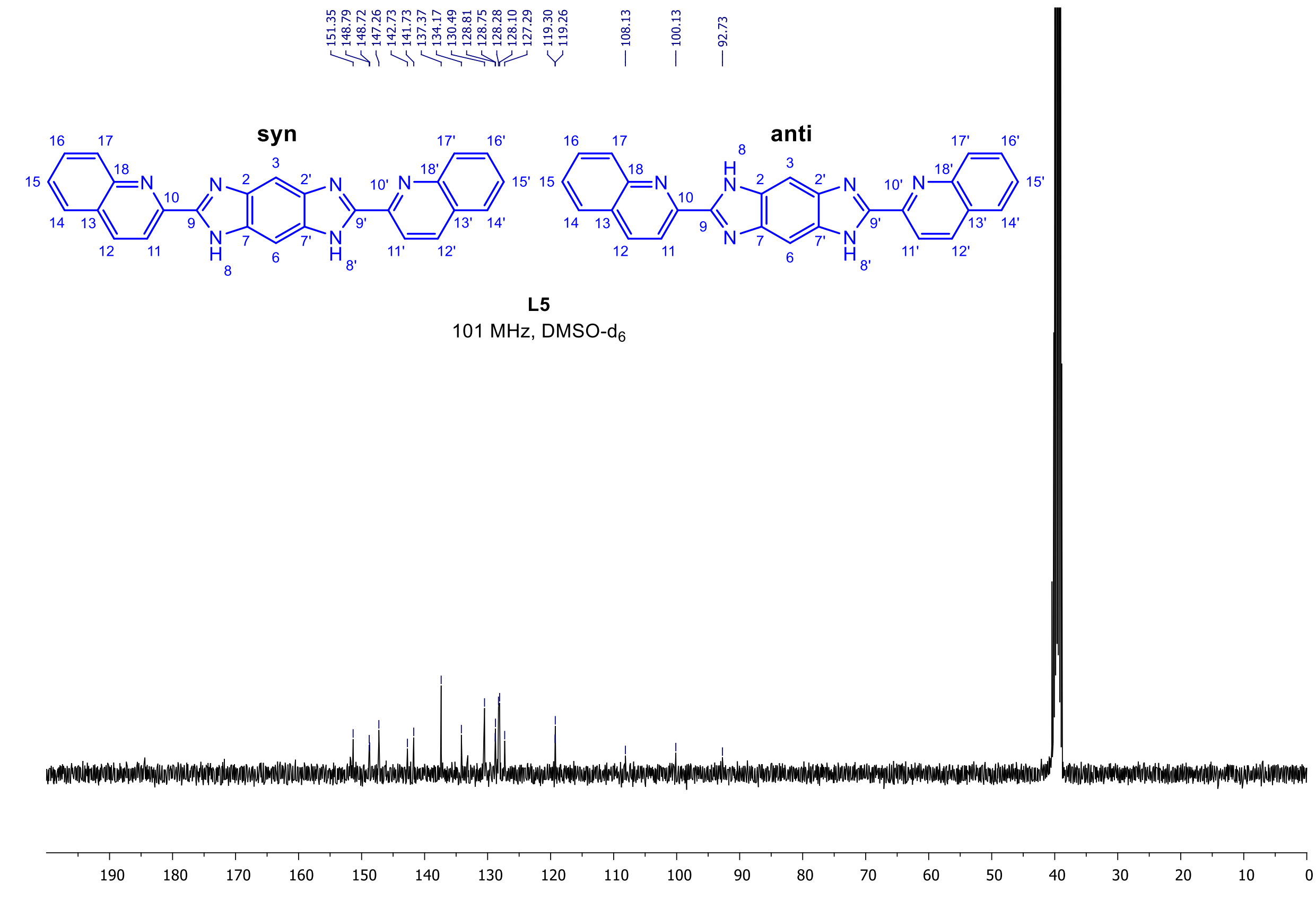
syn
anti
L5
101 MHz, DMSO-d$_6$
151.35
148.79
148.72
147.26
142.73
141.73
137.37
134.17
130.49
128.81
128.75
128.28
128.10
127.29
119.30
119.26
108.13
100.13
92.73
190 180 170 160 150 140 130 120 110 100 90 80 70 60 50 40 30 20 10 0

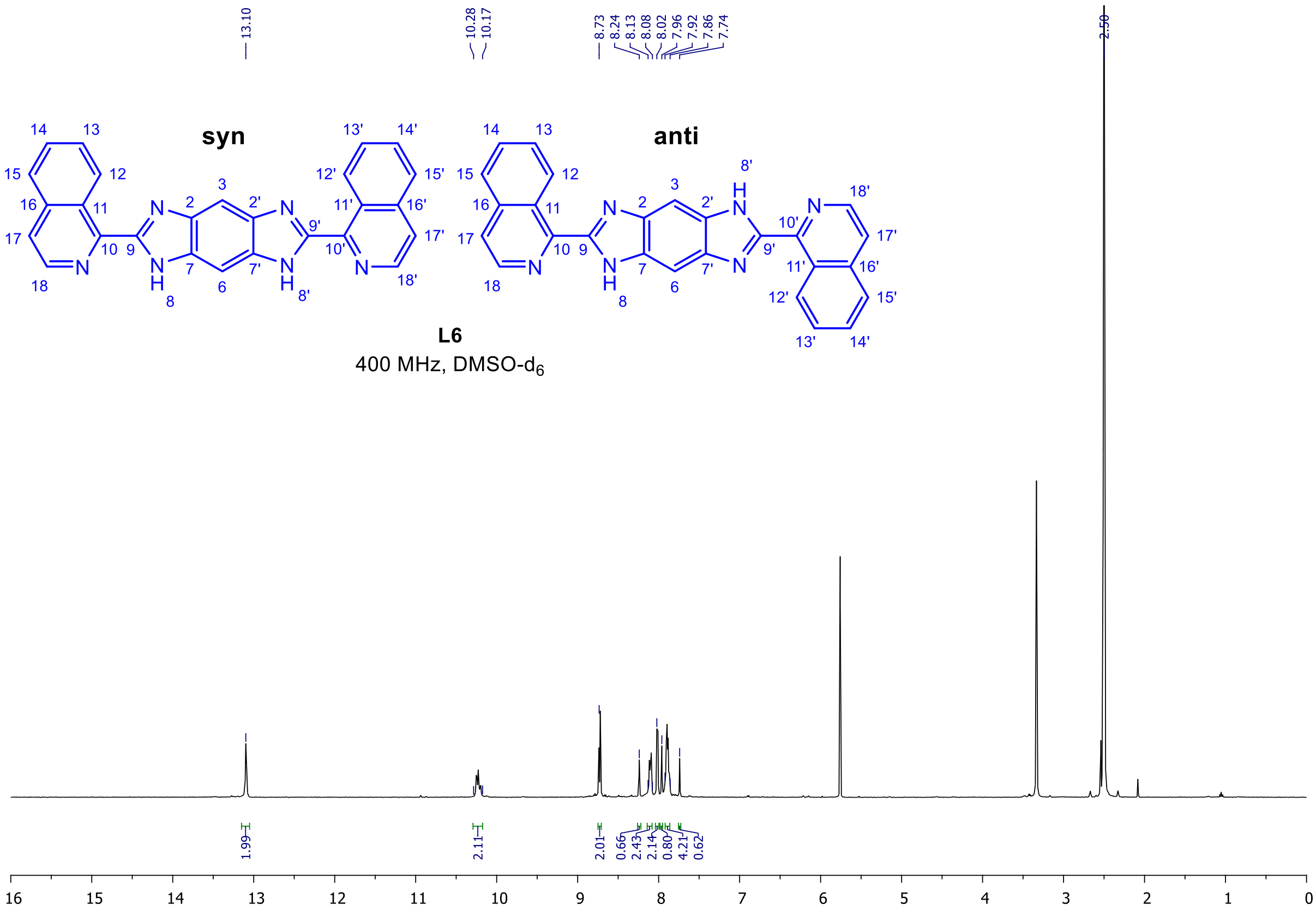
13.10
10.28
10.17
8.73
8.24
8.13
8.08
8.02
7.96
7.92
7.86
7.74
2.50
syn
anti
L6
400 MHz, DMSO-d6
1.99
2.11
2.01
0.66
2.43
2.14
0.80
4.21
0.62
16 15 14 13 12 11 10 9 8 7 6 5 4 3 2 1 0

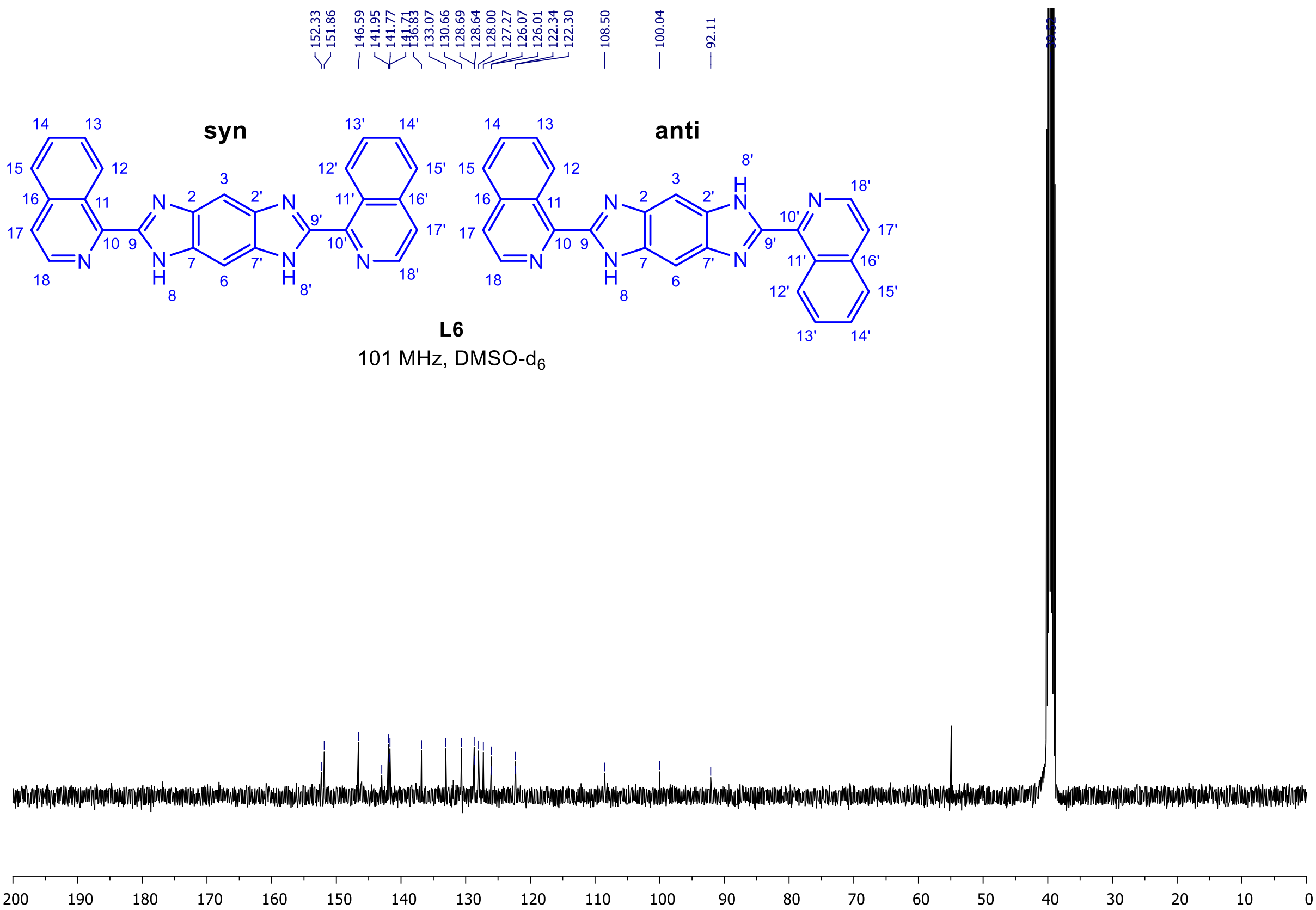
152.33
151.86
146.59
141.95
141.77
141.71
136.83
133.07
130.66
128.69
128.64
128.00
127.27
126.07
126.01
122.34
122.30
108.50
100.04
92.11
syn
anti
L6
101 MHz, DMSO-d6
200 190 180 170 160 150 140 130 120 110 100 90 80 70 60 50 40 30 20 10 0

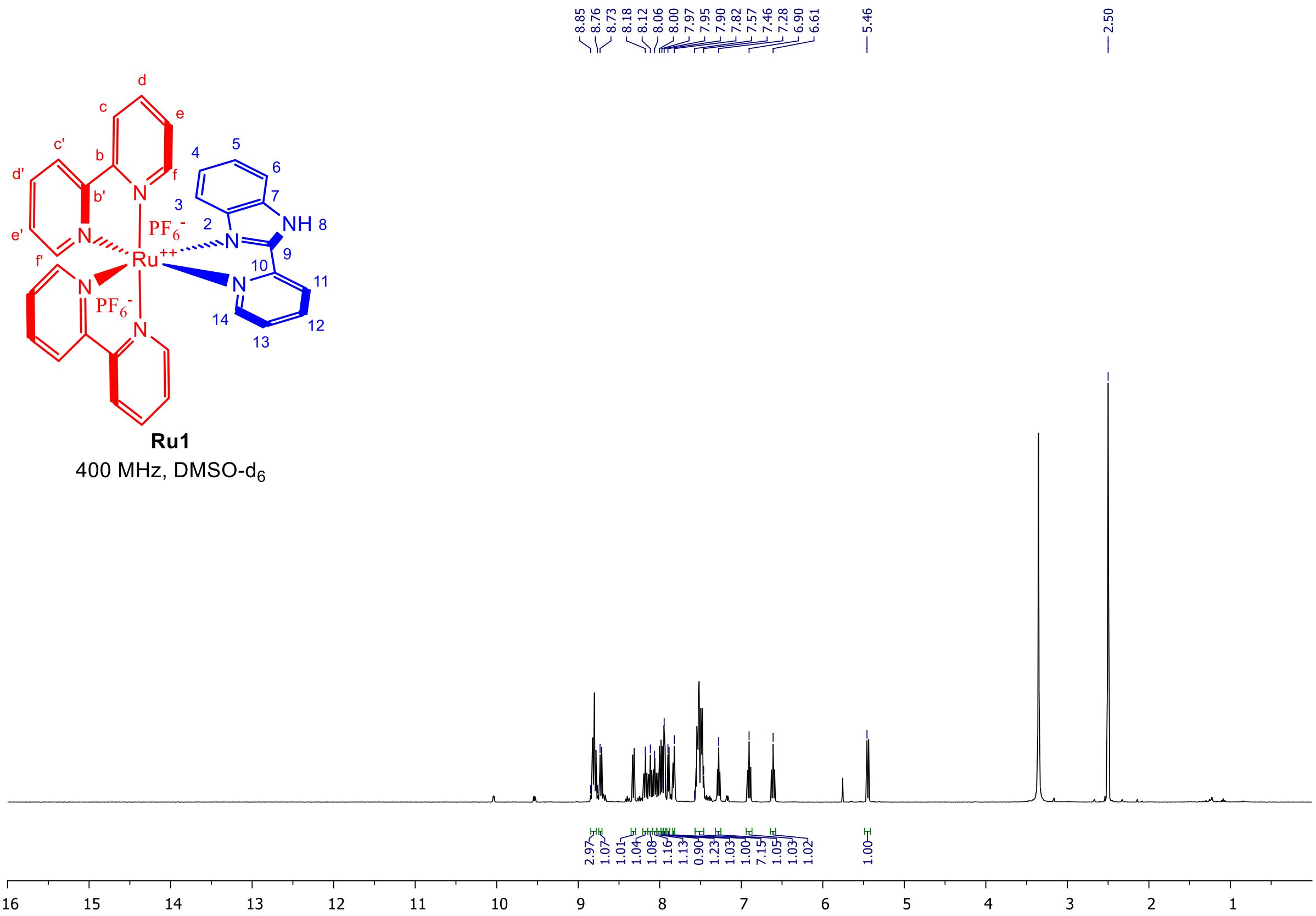
Ru1
400 MHz, DMSO-d6
8.85
8.76
8.73
8.18
8.12
8.06
8.00
7.97
7.95
7.90
7.82
7.57
7.46
7.28
6.90
6.61
5.46
2.50
2.97
1.07
1.01
1.04
1.08
1.16
1.13
0.90
1.23
1.03
1.00
7.15
1.05
1.03
1.02
1.00

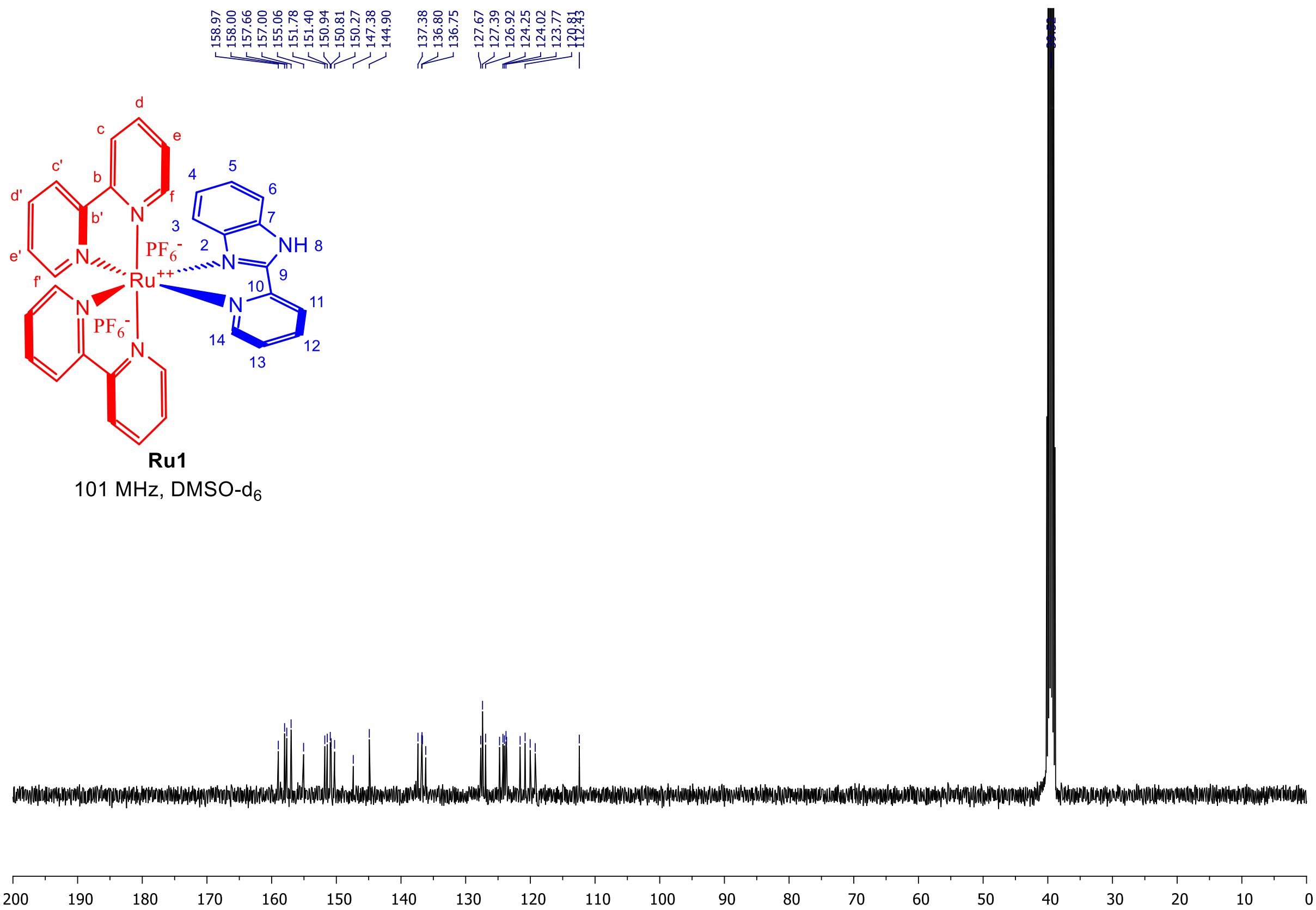
Ru1
101 MHz, DMSO-d6
158.97
158.00
157.66
157.00
155.06
151.78
151.40
150.94
150.81
150.27
147.38
144.90
137.38
136.80
136.75
127.67
127.39
126.92
124.25
124.02
123.77

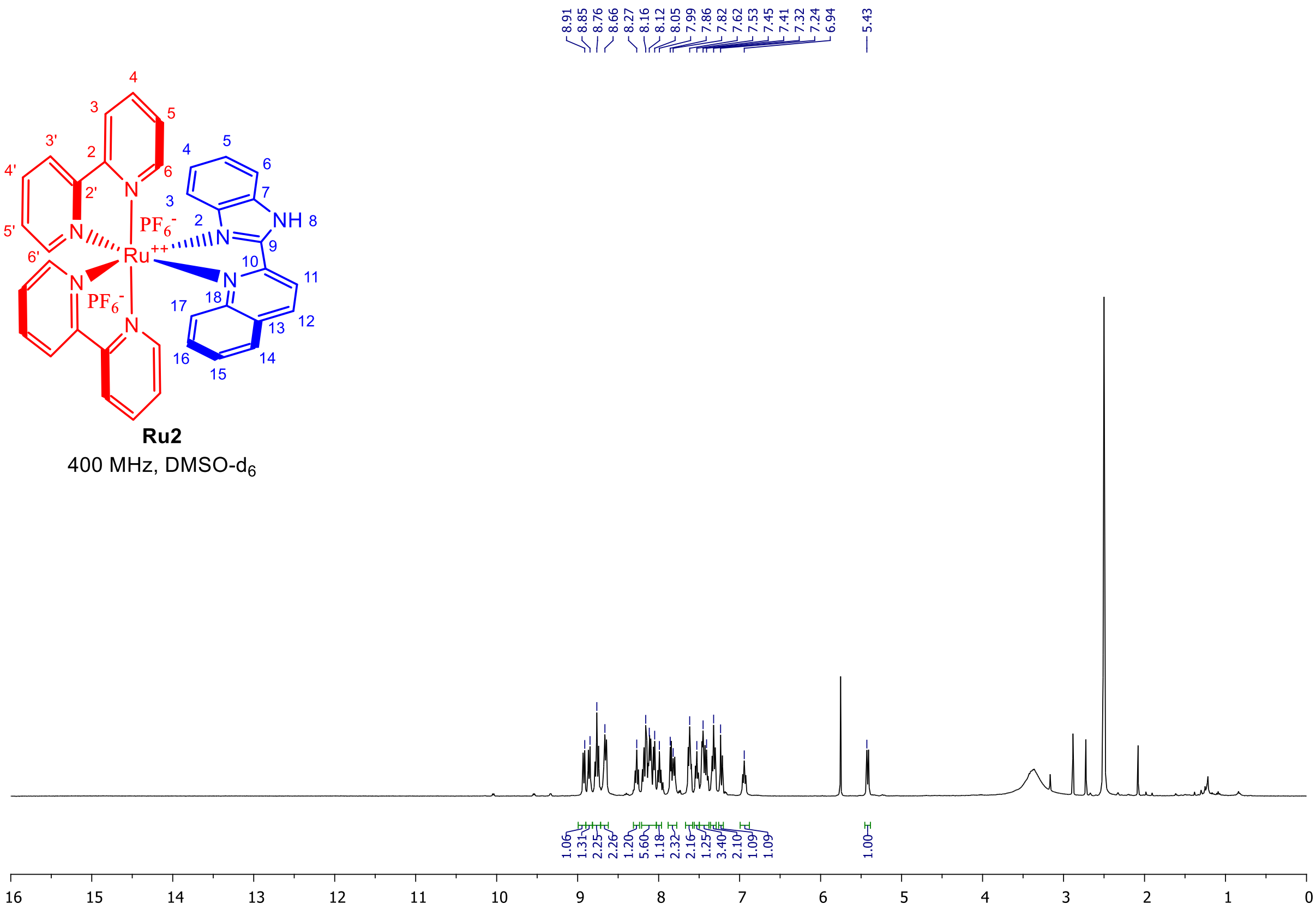
8.91
8.85
8.76
8.66
8.27
8.16
8.12
8.05
7.99
7.86
7.82
7.62
7.53
7.45
7.41
7.32
7.24
6.94
5.43
1.06
1.31
2.25
2.26
1.20
5.60
1.18
2.32
2.16
1.25
3.40
2.10
1.09
1.09
1.00
16 15 14 13 12 11 10 9 8 7 6 5 4 3 2 1 0
Ru2
400 MHz, DMSO-d6

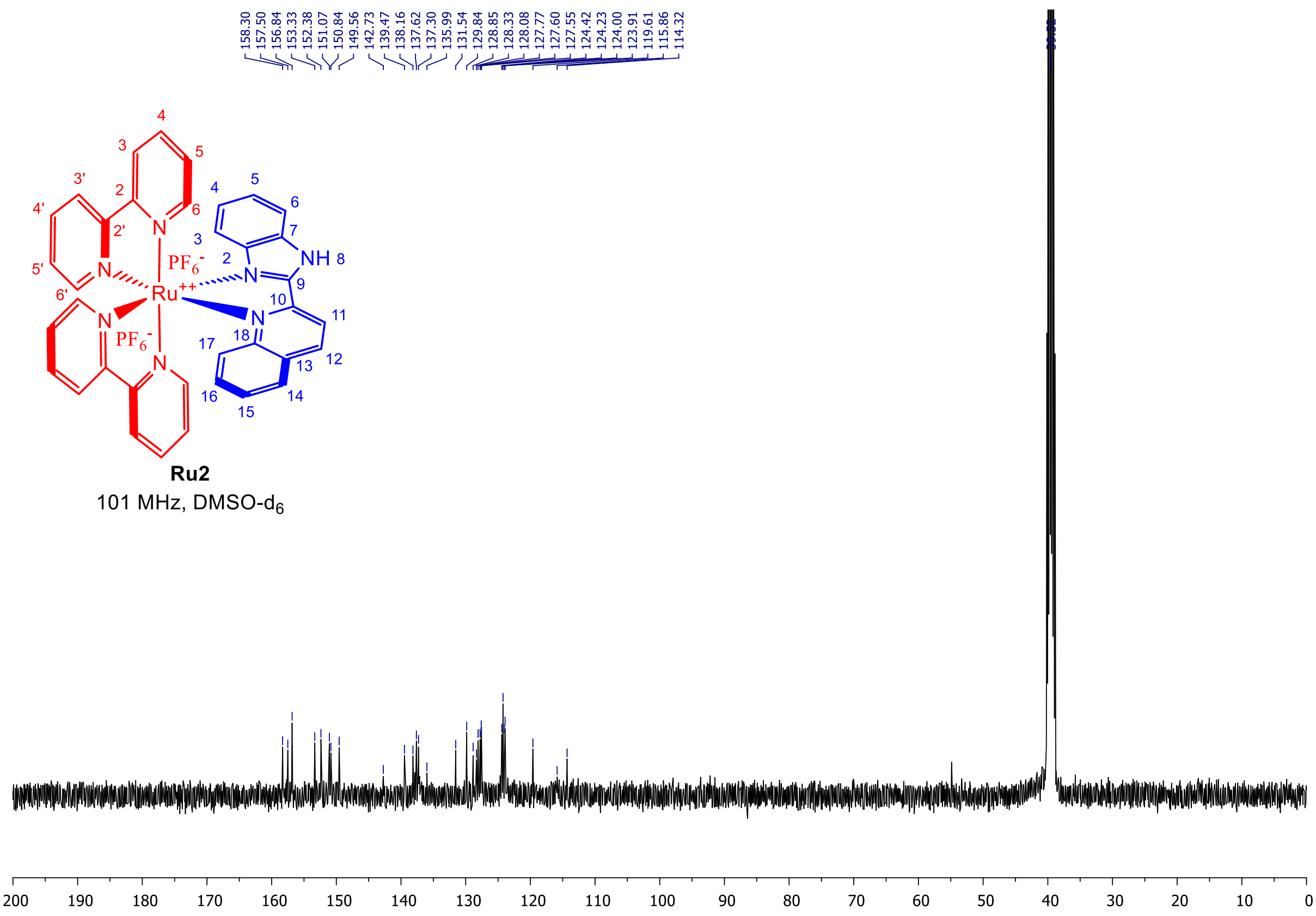
158.30
157.50
156.84
153.33
152.38
151.07
150.84
149.56
142.73
139.47
138.16
137.62
137.30
135.99
131.54
129.84
128.85
128.33
128.08
127.77
127.60
127.55
124.42
124.23
124.00
123.91
119.61
115.86
114.32
200 190 180 170 160 150 140 130 120 110 100 90 80 70 60 50 40 30 20 10 0
Ru2
101 MHz, DMSO-d6

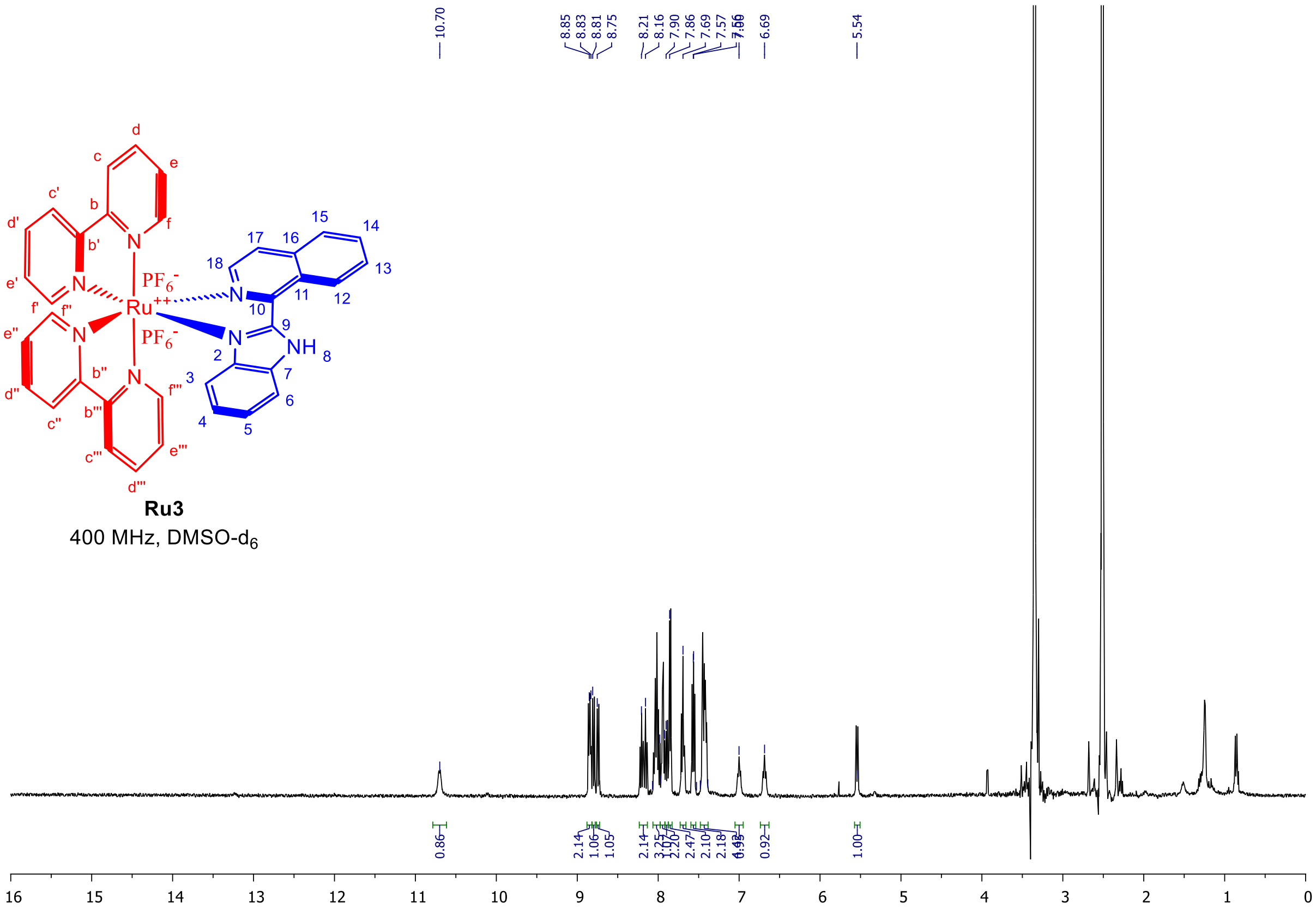
10.70
8.85
8.83
8.81
8.75
8.21
8.16
7.90
7.86
7.69
7.57
6.69
5.54
Ru3
400 MHz, DMSO-d6
0.86
2.14
1.06
1.05
1.00
16 15 14 13 12 11 10 9 8 7 6 5 4 3 2 1 0

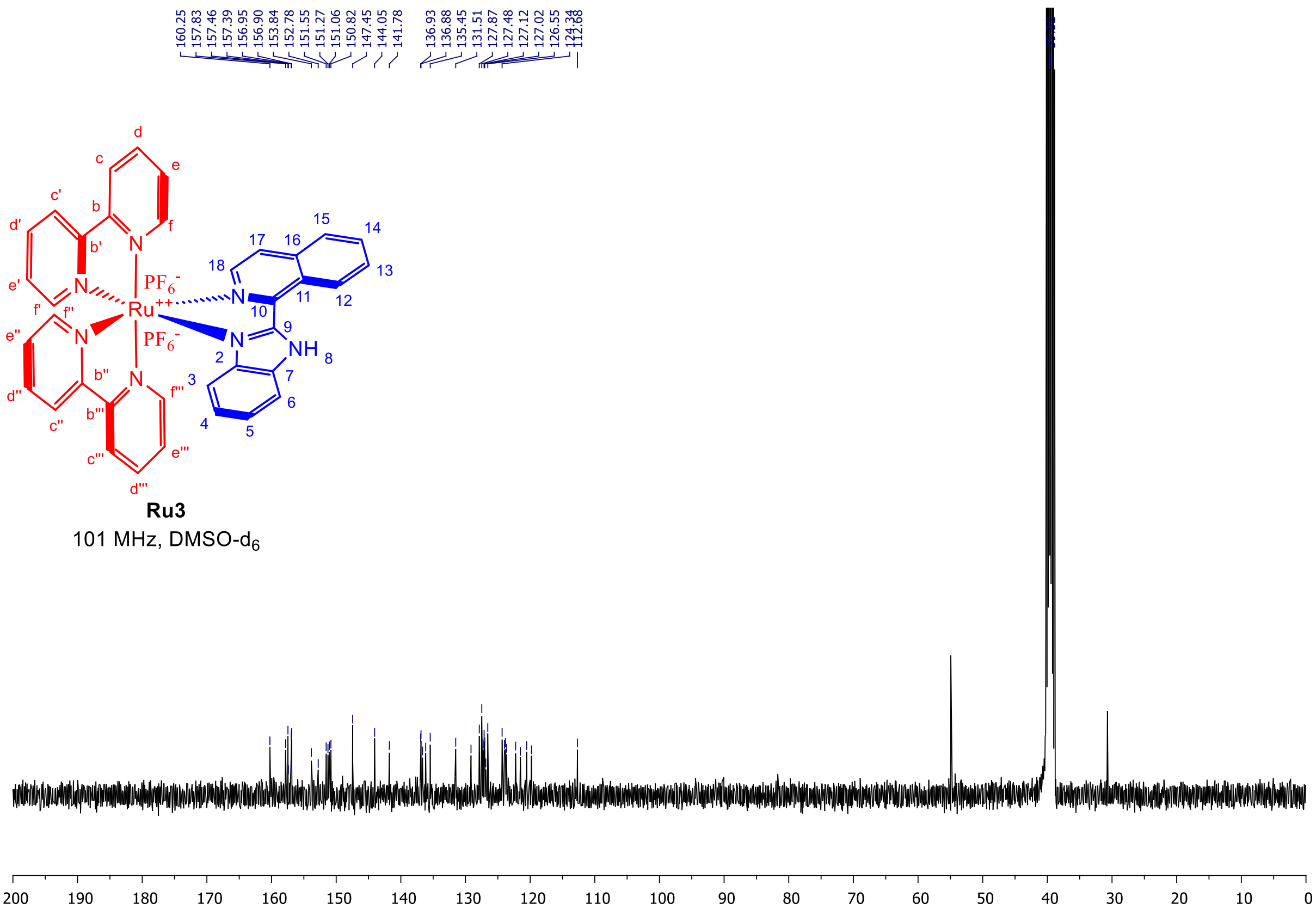
160.25
157.83
157.46
157.39
156.95
156.90
153.84
152.78
151.55
151.27
151.06
150.82
147.45
144.05
141.78
136.93
136.88
135.45
131.51
127.87
127.48
127.12
127.02
126.55
Ru3
101 MHz, DMSO-d6
200 190 180 170 160 150 140 130 120 110 100 90 80 70 60 50 40 30 20 10 0

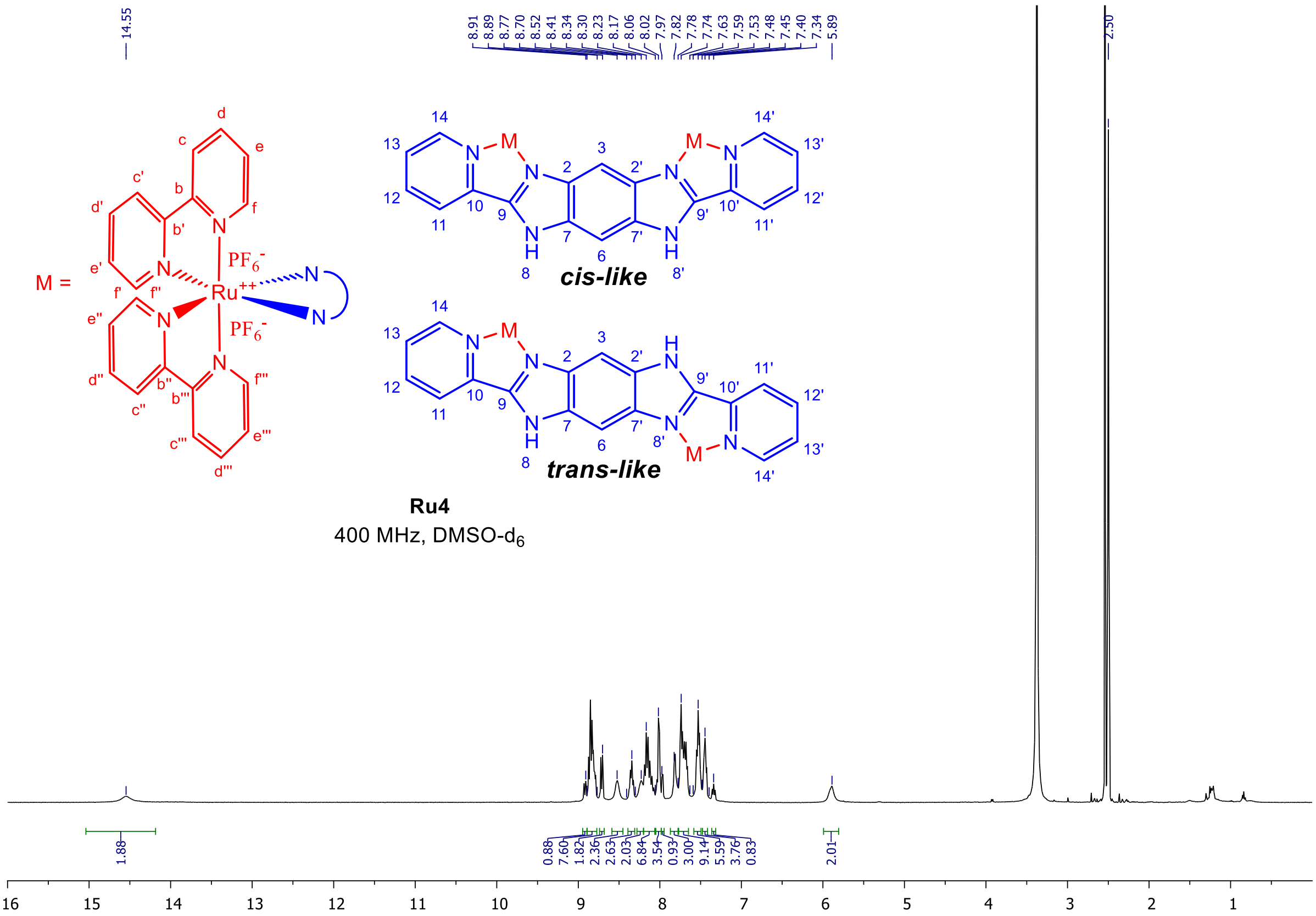
14.55
8.91 8.89 8.77 8.70 8.52 8.41 8.34 8.30 8.23 8.17 8.06 8.02 7.97 7.82 7.78 7.74 7.63 7.59 7.53 7.48 7.45 7.40 7.34 5.89
2.50
M =
PF6-
Ru++
cis-like
trans-like
Ru4
400 MHz, DMSO-d6
1.88
0.88 7.60 1.82 2.36 2.63 2.03 6.84 3.54 0.93 3.00 9.14 5.59 3.76 0.83
2.01
16 15 14 13 12 11 10 9 8 7 6 5 4 3 2 1

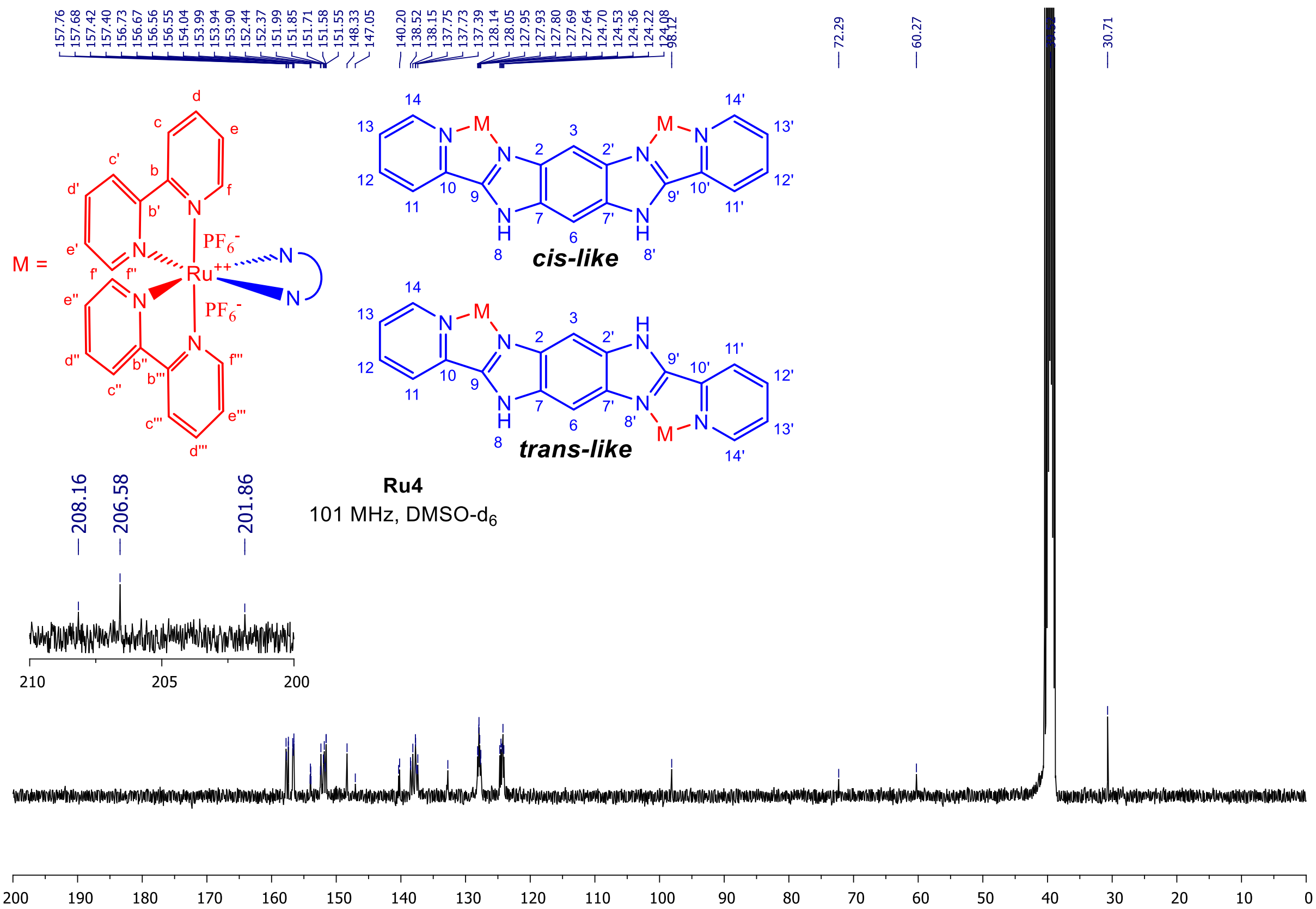
157.76 157.68 157.42 157.40 156.73 156.67 156.56 156.55 154.04 153.99 153.94 153.90 152.44 152.37 151.99 151.85 151.71 151.58 151.55 148.33 147.05
140.20 138.52 138.15 137.75 137.73 137.39 128.14 128.05 127.95 127.93 127.80 127.69 127.64 124.70 124.53 124.36 124.22 124.08 98.12
72.29
60.27
30.71
M =
PF6-
Ru++
cis-like
trans-like
Ru4
101 MHz, DMSO-d6
208.16
206.58
201.86
210 205 200
200 190 180 170 160 150 140 130 120 110 100 90 80 70 60 50 40 30 20 10 0

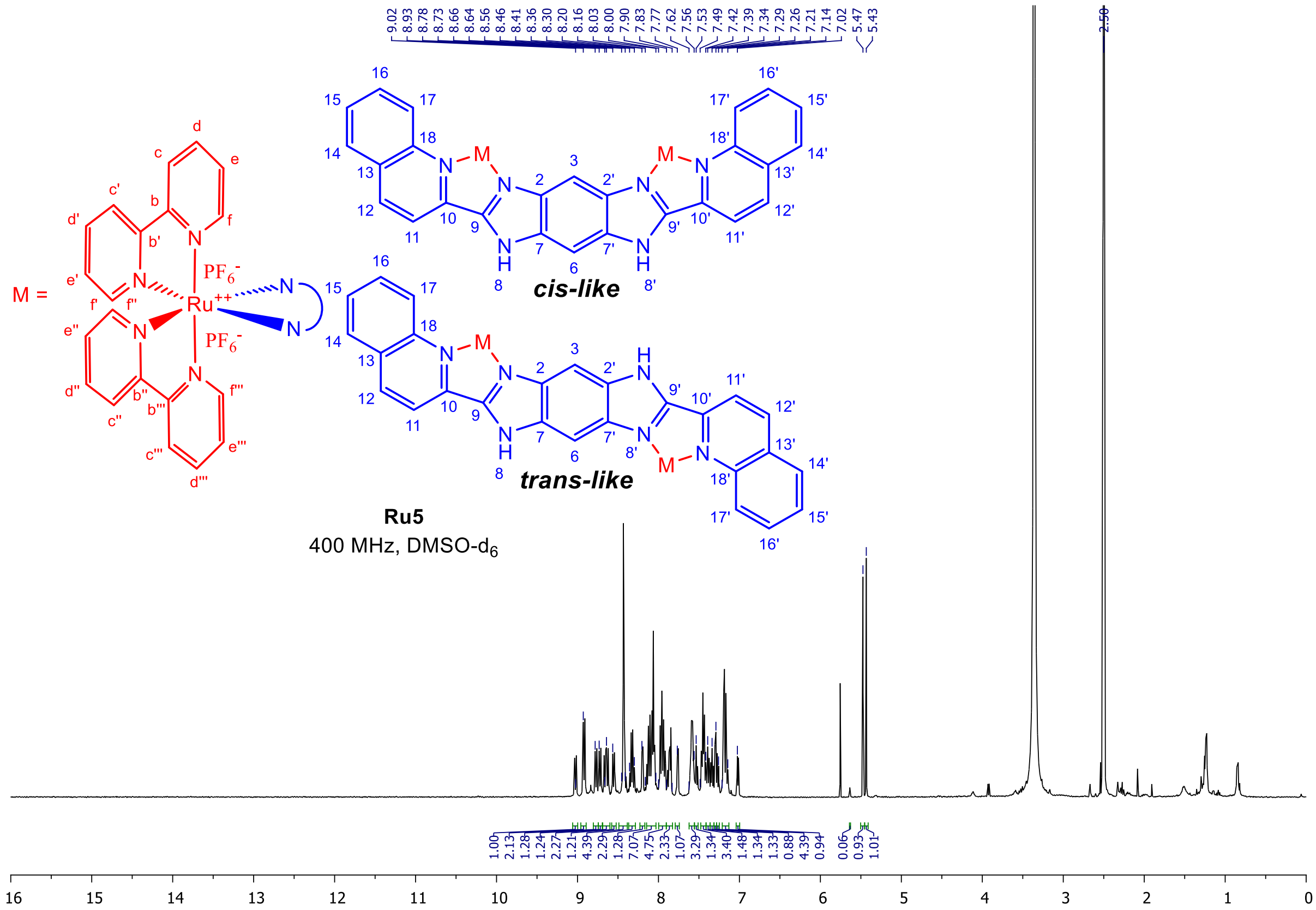
9.02 8.93 8.78 8.73 8.66 8.64 8.56 8.46 8.41 8.36 8.30 8.20 8.16 8.03 8.00 7.90 7.83 7.77 7.62 7.56 7.53 7.49 7.42 7.39 7.34 7.29 7.26 7.21 7.14 7.02 5.47 5.43
2.50
M =
cis-like
trans-like
Ru5
400 MHz, DMSO-d6
1.00 2.13 1.28 1.24 2.27 1.21 4.39 2.29 1.28 7.07 4.75 2.33 1.07 3.29 1.34 3.40 1.48 1.34 1.33 0.88 4.39 0.94 0.06 0.93 1.01
16 15 14 13 12 11 10 9 8 7 6 5 4 3 2 1 0

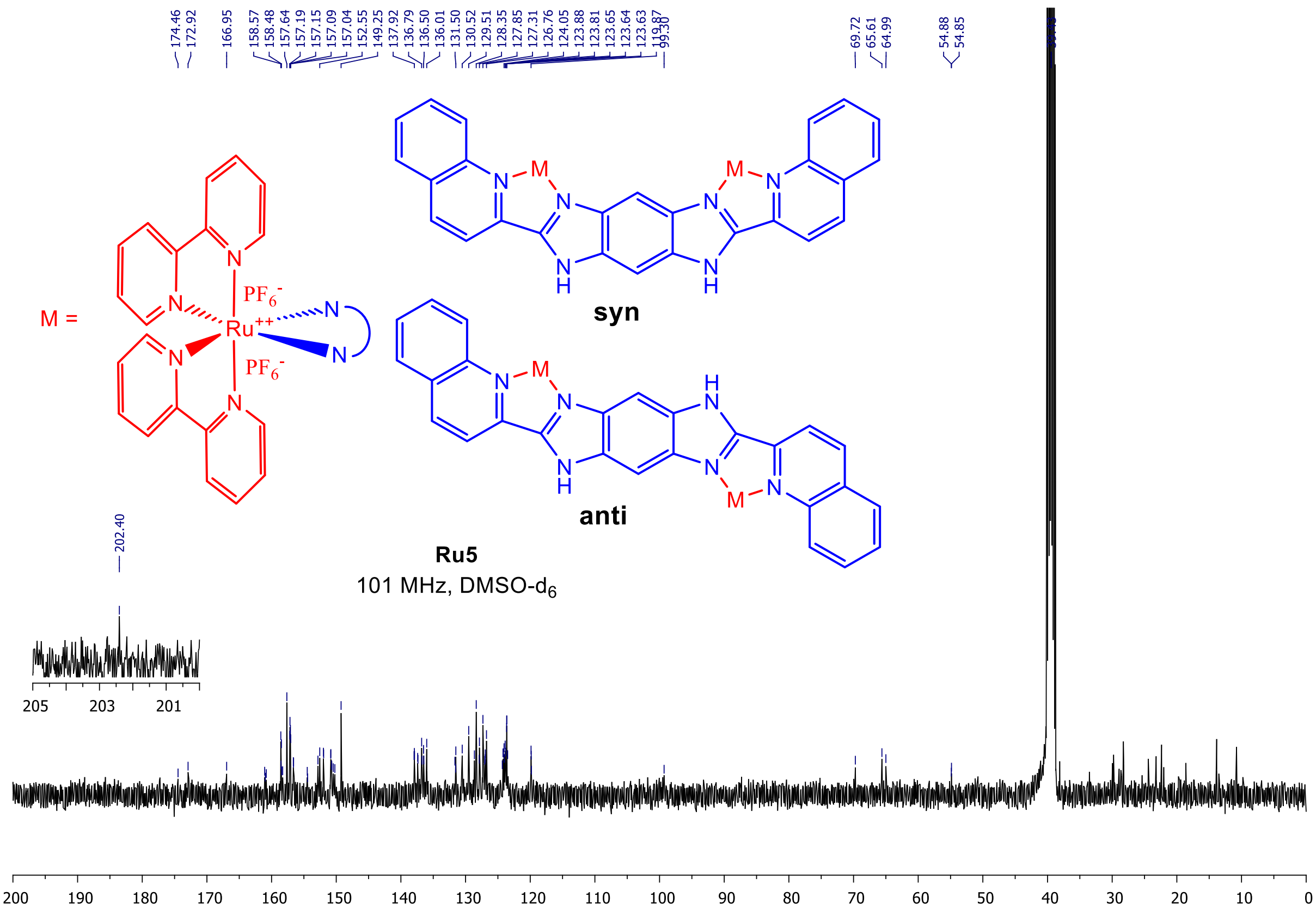
174.46 172.92 166.95 158.57 158.48 157.64 157.19 157.15 157.09 157.04 152.55 149.25 137.92 136.79 136.50 136.01 131.50 130.52 129.51 128.35 127.85 127.31 126.76 124.05 123.88 123.81 123.65 123.64 123.63 119.87 99.30
69.72 65.61 64.99 54.88 54.85
M =
syn
anti
Ru5
101 MHz, DMSO-d6
202.40
205 203 201
200 190 180 170 160 150 140 130 120 110 100 90 80 70 60 50 40 30 20 10 0

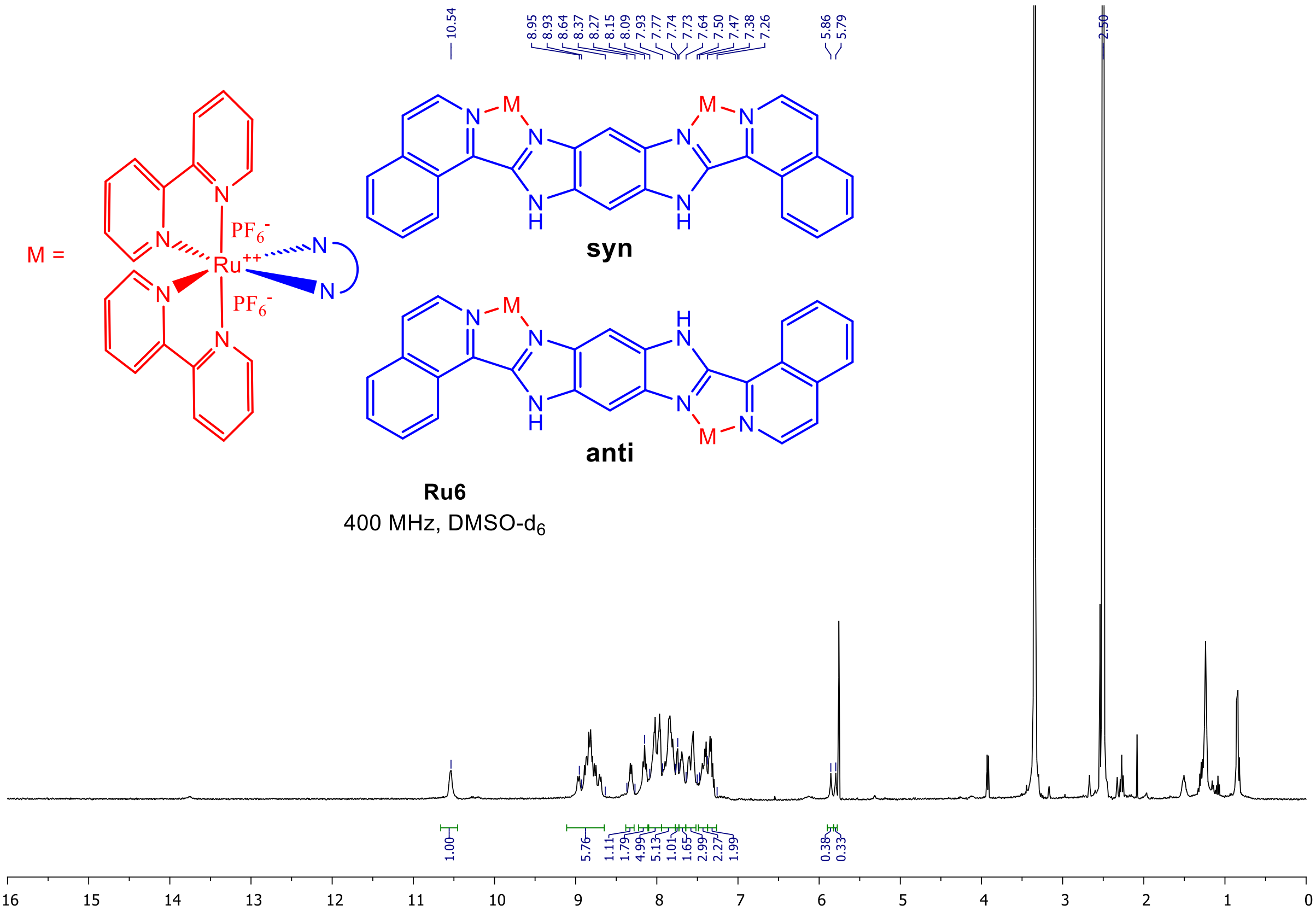
M =
syn
anti
Ru6
400 MHz, DMSO-d6
